# Supplementary material for: Horse Y chromosome assembly displays unique evolutionary features and putative stallion fertility genes
Source: Nat Commun. 2018 Jul 27;9:2945. doi: 10.1038/s41467-018-05290-6 (PMC6063916; doi:10.1038/s41467-018-05290-6)
Supplement: Supplementary file 1 — Supplementary Information [file 41467_2018_5290_MOESM1_ESM.pdf]

Horse Y chromosome assembly displays unique evolutionary features and putative stallion fertility genes

Janečka et al.

• Supplementary Note 1: BAC tiling path of horse MSY

A BAC contig map of the horse MSY (Supplementary Fig. 1) was constructed by sequence tagged site (STS)-content analysis, chromosome walking, and fluorescence *in situ* hybridization (FISH) using the methods described below. This BAC contig map was the basis of the sequencing of the MSY.

BAC library screening. We used known MSY markers [1, 2] and newly generated STSs-from BAC end sequences (BES) to design primers for PCR (Supplementary Data 4). The sequences were masked for repeats with RepeatMasker (http://www.repeatmasker.org/) and the primers were designed with the Primer3 software [3]. The primers were optimized on male and female horse genomic DNA and used to screen by PCR the CHORI-241 (http://bacpac.chori.org/equine241.htm) BAC library. This genomic library is constructed from a male Thoroughbred horse, *Bravo* (Cornell University). If no clones were found in CHORI-241, the markers were additionally screened in TAMU (L. C. Skow, unpublished) and INRA [4] male BAC libraries, constructed from a male Arabian and a male Selle Français, respectively. The BAC DNA was isolated with Plasmid Midi Kit (Qiagen) and end sequenced for STS development using the standard T7 and SP6 or M13 primers and BigDye chemistry. Detailed information about 192 horse MSY BACs is available in Supplementary Data 5.

Chromosome preparations and Fluorescence* in situ *hybridization (FISH). Peripheral blood samples and fibroblast cultures from a male Thoroughbred horse, the DNA donor for the CHORI-241 BAC library, were used for metaphase and interphase chromosome preparations [5]. Mechanically stretched DNA fibers on poly-L-lysine coated glass slides for fiber-FISH were prepared from blood lymphocytes of the same male Thoroughbred according to established procedures [5]. Chromosome preparations of the donkey (*Equus asinus*; EAS), the quagga plains zebra (*Equus burchelli*; EBU) and Hartmann's mountain zebra (*Equus zebra hartmannae*; EZH) were obtained from blood lymphocytes or fibroblast cultures [5].
DNA from individual BAC clones was labeled with biotin-16-dUTP and/or digoxigenin-11-dUTP by nick translation using Biotin- or DIG-Nick Translation Mix (Roche). The labeled probes were hybridized individually or in combinations of 2 or 3 probes to metaphase/interphase chromosomes and DNA fibers. Metaphase FISH was carried out with all BACs to confirm their Y chromosome origin. Interphase and fiber FISH experiments were conducted with selected clones to verify clone order, clone overlaps, copy numbers, and determine the size of gaps between MSY contigs. A minimum of 10 metaphase spreads, 30 interphase cells or ~30 DNA fiber hybridization images were captured and analyzed per each experiment using a Zeiss Axioplan2 fluorescent microscope equipped with Isis v 5.2 (MetaSystems GmbH) software.

The BAC contig map. The BACs were arranged into contigs by STS content mapping which was carried out by PCR with all STS primers on all BAC clones. The final contig map was assembled through stepwise chromosome walking into the gaps. The order and orientation of contigs, and the size of gaps between contigs were determined by interphase and/or fiber-FISH. The tiling path of the horse MSY (Supplementary Fig. 1) comprised 192 partially overlapping BAC clones of which 139 originated from the CHORI-241 BAC library (http://bacpac.chori.org/equine2) (Thoroughbred), 41 clones from the TAMU library (Arabian; L. Skow, unpublished), and 12 clones from the INRA library ([4]; Selle Français), thus representing 3 different Y chromosomes.

The tiling path clones were arranged into 4 BAC contigs leaving 3 small gaps for which no clones were found due to repetitive sequences. The order and overlaps of the BACs were supported by 265 linearly ordered genes, ESTs and STS markers (Supplementary Fig. 1 and Supplementary Data 4). A region of contig I contained BACs with multiple copies in MSY (Supplementary Fig. 1, blue shade) and their tiling path was arranged provisionally based on a set of selected markers. The orientation of individual contigs in relation to each other was confirmed by dual-color interphase FISH. Proximally, the BAC tiling path extended into Y chromosome heterochromatin (HC) and distally into the pseudoautosomal region (PAR), thus spanning over the entire eMSY. Y-specificity of all BACs was confirmed by FISH to male metaphase chromosomes (Supplementary Fig. 2). The majority of clones hybridized exclusively to the eMSY with a cytogenetic location at Yq14-q15 [1, 6], whereas the clones spanning the pseudoautosomal boundary (PAB) hybridized to both Yqter and Xpter. A few eMSY clones shared sequence similarity with autosomal regions: clone 79.4H1 in contig Ia mapped by FISH to MSY and chr16; clone 139C20 in multi copy region Ic to MSY and chr3q; clones 115I17 and 169C6 at the proximal end of contig II to MSY and chr18. The two most proximal clones in MSY tiling path, 54A8 and 69E11, mapped by FISH all over the Y chromosome heterochromatin, as well as in the facultative heterochromatin in chrXq17-q21. A region in the proximal part of contig I (Supplementary Fig. 1, green shade) shared sequence similarity with the pseudoautosomal region (PAR). Clones from this region gave three FISH signals: two in the Y chromosome – in MSY and the PAR-Y, and one in the PAR-X. Copy numbers of the sequences contained in BACs were estimated by FISH in interphase chromosomes. The majority of BACs were single copy, while all 49 clones in contig Ib showed multiple hybridization signals (Supplementary Fig. 2). FISH analysis indicated that the multi copy sequences were repeated moderately from about 2 to 20 hybridization signals per interphase. The tiling path of horse MSY was interrupted by three gaps (Supplementary Figures 1 and 3). GAP2 and GAP3 were flanked by repetitive sequences that predominantly comprised of long interspersed nuclear elements (LINEs) and long terminal repeats (LTRs) as revealed by BES analysis (Supplementary Data 4). GAP1 was proximally flanked by repeats. However, clones 115I17 and 169C6 distal to GAP1 mapped by FISH to both MSY and chr18. Chromosome walking using the 169C6-T7 and 115I17-T7 sequences identified over 10 new BAC clones, which mapped to either chr18 or chr20. All new STS markers derived from the end sequences of the new clones shared sequence similarity only with autosomes – an indication that chromosome walking had stepped off from MSY. The size of the gaps was estimated by interphase and fiber-FISH using combinations of MSY-specific clones around the gaps, *viz.*, 127N19 and 91.4G10 for GAP1; 132K10 and 205D10 for GAP2, and 34A23 and 504H13 for GAP3 (Supplementary Fig. 3). GAP1 and GAP2 were the largest with a clear distance between the flanking clones in interphase nuclei, whereas clones flanking GAP3 overlapped in interphase. We did not observe clone overlaps for any of the gaps by fiber-FISH, which suggests that all three gaps exceeded 500 kb. At this distance, DNA fibers tend to break and clones further apart cannot be visualized together [5]. Based on FISH results, we estimated GAP1 and GAP2 to be approximately 1 Mb each, and GAP3 about 500-600 kb. Altogether, the gaps in horse MSY tiling path counted for less than 3 Mb. The actual size of gaps might be even smaller because FISH analysis with large insert clones involved blocking the hybridization of repetitive sequences due to which the visible hybridization signals are smaller than the actual sequence contained in the BAC.

• **Supplementary Note 2: Horse MSY sequencing and assembly**

The tiling path for sequencing. On average, the overlapping BACs covered horse MSY 4-5 fold. The coverage was the highest (up to 15 fold) in the multi copy region of contig Ib and in repetitive regions around GAP2 (6 fold), and the lowest in single copy regions (2-3 fold) (Supplementary Fig. 1). Lessons from humans show that due to the complex organization of the mammalian Y chromosome, sequencing of the MSY must have higher redundancy than that needed for autosomal and X-linked regions. For example, to sequence the 23 Mb human MSY, 220 highly redundant BAC clones were used [7]. Therefore, to sequence horse MSY, we selected a tiling path of 94 BAC clones (Supplementary Fig. 1 and Supplementary Data 5) with 2-3 fold redundancy in single copy regions, and including 43 out of 49 clones in the multi copy region of contig Ib.

eMSY assembly. The horse MSY draft assembly was 9,497,449 base-pairs (bp) with mean contig N50 of 147,563 bp and maximum contig length of 288,832 bp. The N50 of scaffolds was 299,624 bp, maximum scaffold length 554,834 bp, and maximum super-scaffold length 2.2 Mb (Supplementary Table 1). For the multi-copy region, the N50 of contigs was 67,945 and maximum contig length was 169,861 bp. In the multi copy region, individual sequenced BACs were assembled into nearly complete contigs or scaffolds. For example, maximum contig length for clones 165E24 and 17D15 were 103,017 bp and 149,715 bp, respectively, with N50 scaffold size of 150,349 bp. The sequence content of BACs was compared in MAUVE and BACs that represented unique regions were retained in the assembly. Sequences of individual multi copy BACs were concatenated into 3.56 Mb of sequence.

• Supplementary Note 3: Horse MSY sequence annotation

Repeat content. The eMSY GC content and the content of interspersed repeats was analyzed with RepeatMasker (http://www.repeatmasker.org/). We used default settings and DNA source as "*mammal other than below*", i.e., non-primate, non-rodent, non-carnivore, non-ungulate. The analysis of 9,497,449 bp of eMSY assembled sequence revealed GC level of 40.10 %, which is comparable to human (45.4%) [7], chimpanzee (40.6%) [8], mouse (39.3%) [9], and rat (39.3%) Y chromosomes, and masked altogether 5,173,258 bp (54.47 %) of which 49.43% comprised interspersed repeats. Details of eMSY repeat content are presented in Supplementary Table 2.

Intra-chromosomal sequence identity. We used custom Perl codes, analogous to those applied to the human MSY [7, 10], to analyze the horse MSY sequence for regions with 100% intra-chromosomal sequence similarities. The script used BLAST (http://blast.wustl.edu) to compare sequence segments with word sizes ranging from 20 bp to 1000 bp, each with a step of 20% of the word size. We generated triangular dot plots for hits with 100% identity for 18 different word sizes: from 20 bp to 100 bp with 10 bp intervals and from 100 bp to 1000 bp with 100 bp intervals. Four most informative dot plots are presented in Supplementary Fig. 4. It appeared that the word (motif) size range from 50 bp to 500 bp was the best to highlight the accumulation of 100% identical sequences specifically in the eMSY ampliconic region in the interval between 1 Mb and 4 Mb in the sequence map.

Testis RNASeq and transcriptome assembly. We sought to comprehensively annotate the MSY with testis-expressed transcripts. There is a strong evidence from our previous studies in horses [2] and from studies of other eutherian Y chromosomes [11] that the majority of MSY genes are expressed in testis - some exclusively, others broadly in multiple tissues but always with the inclusion of testis. To date, the only known MSY gene that is not expressed in testis is *AMELY*, which expression is limited to tooth [11]. Therefore, in this study, we focused on testis-expressed genes, particularly as one of the major applications of the annotated reference sequence in stallions is male fertility.

Testis samples were procured during scheduled castration (Animal Use Protocol IACUC 2012-0250 Ref#000147) from two normal mature stallions (H383 – a 3 years-old American Quarter Horse and H452 – a 4 years-old Thoroughbred), two normal mature donkeys (EAS13 – 3 years-old Miniature donkey and EAS18 – a 8 years-old Minature donkey), and two normal mature mules (H357 – a 4 years-old and H358 – a 3 years-old) (Supplementary Table 4).

We extracted high quality (RIN>9.6) RNA using PureLink RNA Mini Kit (Ambion), converted RNA into cDNA, prepared 2 x 100 bp PE TruSeq libraries (Illumina), and sequenced the samples on HiSeq (Illumina) platform. We obtained, on average, 80 million PE reads per sample (Supplementary Table 4). The obtained RNAseq data was applied for eMSY annotation as follows: i) we individually assembled testis transcriptomes of the two horses using Trinity [12] and mapped those to *eMSYv3* annotated assembly, and ii) we mapped RNAseq raw reads from horses, donkeys and mules to the entire horse genome. The latter comprised of the reference assembly EquCab2 (https://www.ncbi.nlm.nih.gov/projects/mapview/stats/BuildStats.cgi?taxid=9796&build=2&ver=2) and our *eMSYv3* annotated assembly (Supplementary Data 2 and 8).

Gene models. We applied the following strategies to annotate the MSY:

1) We analyzed all published eMSY gene sequences [2] and STS markers (Supplementary Data 4) by BLAST (https://blast.ncbi.nlm.nih.gov/Blast.cgi) against the *eMSYv3* assembly. The BLAST alignment locations were inspected and used to construct the assembly and annotate the corresponding sequences.

2) We downloaded all known mammalian MSY mRNA transcripts from Ensembl (http://www.ensembl.org/index.html?redirect=no) and NCBI (https://www.ncbi.nlm.nih.gov/). Where available, the horse sequences were used. If not available, we used either human, or other mammalian orthologous sequences and BLAST analyzed against *eMSYv3* assembly. Each of the hits was inspected and rigorous criteria were used to verify their presence in eMSY: i) there had to be at least 70% sequence similarity; ii) presence of at least 75% of the exons; iii) highly repetitive and fragmentary hits were excluded.

3) Discovery and annotation of novel genes, i.e., those not reported in the horse MSY gene catalogue [2]. First, by mapping RNAseq transcripts to the *eMSYv3* assembly, we identified those that may be transcribed from the Y but were not known before. Next, we BLAST analyzed these sequences against NCBI and Ensembl protein databases to identify potential novel Y genes. Sequences of the genes with high sequence similarity to known proteins were downloaded and BLAST analyzed against the eMSY assembly to refine intron and exon boundaries. If this hit was of high quality, the gene was added to the eMSY annotation.

Assembly and gene model validation. The assembly and gene models were validated by PCR. We extracted sequences for all new and selected known Y genes and designed 88 sets of primers to validate these sequences in the horse Y (Supplementary Data 11). PCR reactions were carried out with DNA templates from normal male and female horses and the results were visualized on a standard 2% agarose gel. We evaluated the results for the presence or absence of amplification, and for the expected product size. In addition, we designed 29 sets of primers to screen horse tissues and examine transcriptional profiles of eMSY genes by reverse transcriptase PCR (RT-PCR), and verify their expression in testes. Where possible, the RT-PCR primers were designed from adjacent exons, spanning the intron (see below *Horse MSY expression analysis*; Supplementary Fig. 5 and Supplementary Data 11). The RT-PCR experiments also contributed to validation of exon-intron structure, exon-intron boundaries and eMSY gene models in general.

• Supplementary Note 4: Transcriptional profiling of horse MSY genes

Reverse transcriptase PCR (RT-PCR). We used the published information about the transcriptional profiles of the previously known horse MSY genes/transcripts [2] and carried out reverse transcriptase PCRs (RT-PCR) on a panel of adult tissues for all newly identified genes (Supplementary Fig. 5a). Transcriptional profiles of select ampliconic genes were also analyzed in a panel of fetal of one 50 days-old male fetus (Supplementary Fig. 5b). The small scope of RT-PCR in fetal tissues was due to limited availability of these tissues. All RT-PCR experiments were carried out in duplicate, and in case of discordant results, additional experiments were conducted until consistent results were obtained.

Testis RNAseq. In addition, we generated RNAseq data for adult horse, donkey and mule testis. While the primary goal of testis RNAseq was to facilitate eMSY annotation (see above *Horse MSY sequence annotation*; Supplementary Data 8 and Supplementary Table 4), the data also complemented RT-PCR results, and provided preliminary comparative information about eMSY gene expression in the testis of the horse, the donkey and their sterile hybrid, the mule.

Equus caballus FASTA reference genome for EquCab2 and GTF gene model version 87 were downloaded from Ensembl (ftp.ensembl.org/pub/release-87/fasta/equus_caballus/dna/, ftp.ensembl.org/pub/release-87/gtf/equus_caballus). Reference chromosomes were concatenated into a single FASTA file, with addition of the newly assembled *eMSYv3* contig, and indexed using STAR genomeGenerate (10.1093/bioinformatics/bts635). The gene model was converted to GFF3 format and modified to include the addition of MSY annotations determined in this study (for GFF3 lines added, see Supplementary Data 12).

Reads from the six TruSeq libraries (H383, H452, EAS13, EAS18, H357 and H358; Supplementary Table 4) were aligned to EquCab2 with STAR two-stage aligner using non-stringent mapping parameters (outFilterMismatchNoverLmax 0.05) to allow divergent read mapping in across species. Initial read mapping utilized the modified GFF3 as the sjdbGTFfile and an sjdbOverhang of 99 and the --sjdbGTFtagExonParentTranscript flag as parent to accommodate the GFF and added data. The generated high confidence collapsed splice junctions file SJ.out.tab was then used as the sjdbFileChrStartEnd input to re-generate the EquCab2 genome, followed by a second alignment with previously used parameters. Reads that map to multiple locations would normally be ignored in subsequent read counting. However, due to the high-homology paralogs associated with many MSY genes, those read mappings were modified post-alignment by altering their NH:i:value to be 1 and their MAPQ value to be 255, and the 0x100 bit in the second SAM column FLAG as unset. Read counting was performed using the Python framework HTSeq (10.1093/bioinformatics/btu638) on an exon level in union mode with idattr as parent and the modified GFF3 as the gene model. A list of read coverage per gene per-individual is available in Supplementary Data 1. Summary information about the expression profiles of eMSY genes in the horse, based on RT-PCR, cDNA selection and testis RNAseq, is presented in Supplementary Data 6.

Discrepancies between RT-PCR and testis RNAseq. Overall, the RT-PCR results of this and our previous study [2] were in agreement with horse testis RNAseq results (Supplementary Data 6). Two genes, *STSY* and *SYPY*, gave consistent negative results with both approaches, suggesting likely pseudogenization. However, several genes that were originally discovered by testis cDNA selection [2] (thus, by definition transcribed in testis) and with unambiguous expression in testis by

RT-PCR, showed low or very low testis expression by RNAseq read alignment (Supplementary Data 6, Supplementary Data 8 and Supplementary Fig. 6). The discrepancy was most pronounced for *ANOS1Y* , *ARSFY*, *ETY4*, *OR8J2Y*, *OR8K3Y*, *TIGD1Y*, and *YIR2* with zero RNAseq reads aligned to eMSY, though RT-PCR indicated testis-limited (*ANOS1Y*) or broad expression (Supplementary Fig. 5a). Likewise, RNASeq read alignment was very low for *SHROOM2Y* (5.5) and for all 9 copies of *CUL4BY* (1.5 -4.0), while RT-PCR indicated that *SHROOM2Y* is broadly expressed and *CUL4BY* is predominantly expressed in both adult testis [2] and fetal gonads (Supplementary Fig. 5a,b).

Since the RT-PCR results were consistent between 2 or more separate experiments, and because several of these genes were discovered by testis cDNA selection, we infer that the RNAseq/RT-PCR discrepancy is likely due to technical reasons. In general, RNAseq coverage across transcripts is influenced by biases introduced during various steps, such as random hexamer priming and cDNA synthesis [13], ligation, amplification, and sequencing [14]. It must be noted that the RNAseq protocol used in this study did not involve PCR amplification, so we likely missed low abundance transcripts. These transcripts, however, were recorded by RT-PCR. Also, due to repetitive elements, high homology between paralogs, incomplete genome reference sequence, and inaccuracies in transcript annotation, quantification of RNAseq requires consideration of read mapping uncertainty, which may bias transcript abundance estimation [15]. This is partially influenced by the reality that *de novo* construction of transcriptomes are often not sufficient to detect certain transcripts and/or cover their entire length [16]. Further, the TruSeq library method used involves poly(A)$^{+}$ enrichment, which is highly sensitive to transcript degradation, and may bias against representation of partially degraded transcripts [17]. Lastly, discrepancies for ampliconic genes, such as *CUL4BY*, *ETY4*, *TIGD1Y*, *YIR2*, are influenced by errors in the assembly of these complex sequences. As noted above, the assembly of eMSY ampliconic regions remained tentative and is a subject for re-sequencing and –assembly in the future.

• Supplementary Note 5: MSY testis transcripts in horse, donkey and mule

Inclusion of donkey and mule testis RNASeq served two purposes. Firstly, we aimed to refine comparative information between horse and donkey MSY gene content and expression profiles. Secondly, because the mule is a sterile horse-donkey interspecific hybrid with impaired spermatogenesis, genes differentially expressed in the mule testis may reveal those critical for normal spermatogenesis in the horse and donkey.

Horse-donkey comparison. In 2011 [2] we showed that of the 37 horse MSY genes known at that time, 29 genes were present (according to PCR on male and female genomic DNA) in donkey MSY. Thus, the MSYs of the two species are rather similar in gene content which was confirmed and refined in this study by comparative testis RNAseq analysis (Supplementary Data 8 and Supplementary Fig. 6). We also confirmed and refined main differences between the two MSYs. As already observed in 2011 [2], donkey does not carry male specific *ETSTY7* sequences (alias *ZNF33b*) as indicated by the same PCR amplicon pattern of these sequences in male and female donkeys [2]. Expression analysis by RT-PCR in donkey testis in the prior study indicated no or very low-level transcription. Here we clarify and refine these observations and show by FISH that *ETSTY7* is restricted to the X chromosome in donkeys (Supplementary Note 6 and Supplementary Fig.7**)** with low-level transcription in tests as revealed by RNAseq. Another clearly different transcript between the donkey and horse MSYs was *ETSTY2.* Though *ETSTY2* sequences are present in MSY in both species [2], it is not expressed in donkey testis but is among highly abundant MSY transcripts in the horse (Supplementary Fig. 6). In addition, we noted absence or very low abundance of *SH3TC1Y* transcripts in the donkey and moderate abundance in the horse. Other than these 3 examples, there was surprisingly high concordance between the abundance of horse and donkey MSY transcripts in testis as measured by RNAseq alignment to horse eMSY assembly. Though, we anticipate that a more refined comparative analysis will reveal more structural and functional differences between the two MSYs.

Horse-donkey comparison with the mule. Mule is a sterile hybrid of a male donkey and female horse, and carries the Y chromosome of the donkey. While we observed overall similar alignment patterns for donkey and mule testis RNAseq reads, some transcripts showed dysregulation in the mule. Of these, the only clearly downregulated transcript in the mule was *HSFY*, while in horse and donkey *HSFY* transcripts were of medium abundance. More curiously, several known eutherian MSY genes, such as *TSPY*, *UBA1Y*, *RBMY*, *KDM5DY*, *DDX3Y*, *UTY*, *SRY*, *TXLNGY*, *TBL1Y* and equid specific transcript *ETY7*, showed clear upregulation in mule testis (Supplementary Fig. 6 and Supplementary Data 8). Genome-wide analysis of 29,371 transcripts (Supplementary Data 1) showed that 839 differed by greater than 30x read depth in both donkey and horse when compared to mule. Of these, 268 have no Ensembl annotation. Of the remaining 571 transcripts, 543 were functionally mappable using GO enrichment with PANTHER 13.1 (10.1093/nar/gks1118). The overrepresentation test with GO Ontology database built 2018-02-02 produced 36 over-represented biological processes at FDR < 0.05, of which at least 30 are directly involved in male fertility. These observations are of potential significance for the discovery of MSY and other genes critical for stallion fertility. However, our current knowledge about the functions of MSY genes in horses/equids is too shallow for drawing any conclusion about functional implications of these observations. For any further speculations,

focused and thorough functional annotation of the horse Y chromosome is needed either as a separate project or as a part of the equine FAANG (Functional Annotation of Animal Genomes) initiative.

• Supplementary Note 6: Horizontal transfer

Discovery and validation. Initially, the eMSY ampliconic transcript *ETSTY7* sequences were analyzed by BLAST and the results showed high similarity to the genome assembly scaffolds of equine intestinal parasite *Parascaris equorum*, in particular to scaffolds PEQ_contig0000339 (LM491555; 88%; 0.0) and PEQ-contig0002468 (LM465228; 95%; 6e-17). Because GenBank has only a single source of genome assembly for *Parascaris* spp. (due to ambiguities in the systematics of these parasites [18], we prefer not to refer to a specific species) in GenBank, there was no comparison for bioinformatics check for possible contamination of the parasite sequence with the host DNA. Therefore, we conducted a series of rigorous wet lab experiments to verify that the similarity between horse MSY and *Parascaris* spp. indicates true horizontal transfer (HT) and is not a result of contamination.

<u>*Parasite specimen*</u> were collected from affected horses as described elsewhere [18]. We used the following *Parascaris* material (Supplementary Table 6): 5 different adult individuals, which were isolated from the small intestine of one male and one female young horse; two individuals of L4-stage larva; two sets of eggs isolated from the uterus of two different female worms; dissected body wall, intestine and gonads obtained and pooled from 3 different worms. It is important to note that before freezing, adult worm A3 was incubated *in vitro* for 48h, thus reducing chance of contamination with host DNA.

<u>*Parasite DNA isolation:*</u> DNA was isolated from eggs, L4, dissected adult organs and whole worms. The tissue was homogenized in a mortar by crushing with a pestel in a small amount of liquid nitrogen and cell lysis solution (Qiagen). The material was incubated in cell lysis solution with 10 mg/mL Proteinase K at 57 ^{o}C overnight. The DNA was isolated with standard phenol-chloroform method using heavy 2.0 mL phase lock gel tubes (5Prime) for the separation of aqueous and organic layers. The DNA was precipitated with isopropanol, washed with 70% ethanol and resuspended in ddH_2O. This was followed by a column-based clean-up using Qiagen DNeasy Blood & Tissue kit and the manufacturer's protocol.

<u>*DNA quality evaluation.*</u> *Parascaris* DNA was quantified by NanoDrop spectrophotometer and a 1 uL aliquote was checked on a 1% agarose gel. As a rule and regardless of the source, *Parascaris* DNA was fragmented compared to the high molecular weight gDNA we isolated from horses. This was an important consideration while attempting to amplify large (>600 bp) PCR products.

<u>*Primers and validation by PCR*</u> was done with three types of primers (Supplementary Data 11**)** as follows:

1. ***Horse-parasite shared sequences.*** Three sets of primers were designed from sequences that showed similarity between *Parascaris* and horse. Primers ***ETSTY7-3ex3*** were designed from eMSY transcript *ETSTY7* copy 3 exon3. These primers had 3 mismatches with *Parascaris* contig0000339 (LM491555.1). Primers ***PEQ339.1*** and ***PEQ339.4*** were designed from *Parascaris* contig0000339 and had 1 and 0 mismatches with *ETSTY7* copy 3, respectively. The 3 primer-sets were used to show that the corresponding sequences are truly present in both the parasite and horse/equid genomes (Supplementary Fig. 8a).

2. ***Horse-specific sequences.*** Next, we used primers for known horse-specific multicopy sequences, such as mitochondrial DNA (mtDNA; 4 primer sets), autosomal copy number variable regions [19] (6 primer sets) and multicopy *TSPY* from eMSY. PCR experiments with these primers on multiple *Parascaris* gDNA templates and horse controls resulted in horse amplicons

only (Fig. 6 and Supplementary Fig. 8b), indicating that the adult parasites, larvae and eggs are not contaminated with host DNA.

3. *Parasite-specific sequences.* Two sets of primers, ***PEQ001.1*** and ***PEQ001.2***, were designed from *Parascaris* spp. genomic sequences (PEQ_scaffold0000001; LM462759) that shared no similarity with eMSY or any other part of the horse genome. We tested these primers on multiple parasite gDNA templates, horse gDNA and horse BAC DNA, and observed amplification in parasite only (Fig. 6 and Supplementary Fig. 8c). The results indicate that horse gDNA or BAC clones are not contaminated with parasite sequences.

Based on these PCR analyses we do not find evidence about horse-to-parasite or parasite-to-horse DNA contamination, thus supporting the presence of true HT between the species.

Sanger sequencing: ETSTY7-3ex3 primers were used to amplify PCR products from DNA isolated from 6 different *Parascaris* spp samples (Supplementary Table 6): two adult individuals, A0.4 and A1, originating from different hosts; adult gonads and body wall; eggs E0.1 and L4 larval stage. The PCR products were purified from unincorporated nucleotides and polymerase through PCR clean up spin columns (Qiagen), and sequenced with forward and reverse primers using BigDye chemistry. The sequencing products were resolved in an ABI 3730 sequencer. The *Parascaris* spp. sequences were aligned with *ETSTY7*-3 exon 3 using NCBI BLAST 2 sequences and showed 92-96% identity between the two species. The sequences were used to construct the *ETSTY7* phylogenetic tree in Fig. 6.

***ETSTY7* sequence distribution in equids.** We investigated the presence and distribution of *ETSTY7* sequences in the genomes of equids (donkey, Plains zebra and Hartmann's mountain zebra) and Perissodactyls (rhinoceros) by FISH on metaphase chromosome (Fig. 6 and Supplementary Fig. 7**)**. The hybridization probe was a biotin-labeled 612 bp PCR product of primers *ETSTY7*-copy3, exon3 F: 5'-ACACCTCGGCCTAGAGAACA-3'; R: 5'-TGACTGAAGCAGTGGTGAGG-3' (Supplementary Data 11). Note that due to the resolution limits of FISH [5], small PCR products are typically not suitable for obtaining detectable hybridization signals. However, FISH with *ETSTY7* PCR product resulted in clear and consistent hybridizations in all studied equids (Supplementary Fig. 7). The results suggest that *ETSTY7* sequences are present in multiple copies in all equids genomes and that horizontal transfer with *Parascaris* spp. dates back to more than 5 MYR, preceding the divergence of modern equids [20]. On the other hand, *ETSTY7* showed distinct distribution patterns in the four equid genomes: Y and X in the horse; X only in the donkey; both sex chromosomes and subtelomeres of multiple autosomes in the two zebra species. The findings suggest a dynamic nature of these sequences in equid genomes, though further studies are needed to understand their functions. In contrast, no hybridization with *ETSTY7* was detected in the rhino (Supplementary Fig. 7). This may indicate that the sequences are too diverged and/or with too few copies for detection by FISH, or that *ETSTY7*-homologous sequences are not present in other Perissodactyls. To test the latter, we conducted PCR with *ETSTY7*-3 ex 3 primers using gDNA of equids, Perissodactyls (rhino, tapir) and a random selection of mammals from diverged eutherian orders. *ETSTY7* was amplified in horses and equids only, and not in rhino, tapir or other mammalian species (Supplementary Fig. 8).

Supplementary Table 1. Metrics of horse MSY sequence assembly

Section of the map	Length (base pairs, bp)	Start (bp)	End (bp)	N50 contigs (bp)	Max Contig (bp)	N50 Scaffold (bp)	Max Scaffold (bp)
Contig Ia (single copy)	1,122,312	1	1,122,312	15,461	93,459	203,697	372,289
Contig Ib (multi copy)	3,558,619	1,122,313	4,680,932	84,850	149,715	180,613	250,326
Contig Ic (single copy)	2,039,080	4,680,933	6,720,013	76,475	130,524	538,439	787,619
Contig II (single copy)	1,307,465	6,720,014	8,027,479	147,563	288,832	299,624	554,834
Contig III (single copy)	779,909	8,027,480	8,807,388	147,563	288,832	299,624	554,834
Contig IV (single copy)	690,064	8,807,389	9,362,219	147,563	288,832	299,624	554,834
**TOTAL**	**9,497,449**						

Supplementary Table 2. Horse MSY repeat content

Repeat content of the 9,497,449 bp horse MSY sequence as revealed by RepeatMasker: http://www.repeatmasker.org/.

Repeat class	Repeat sub-type	Number of elements	Length, bp	% of sequence
SINEs		2,334	454,155	4.78
	MIRs	628	82,664	0.87
LINEs		4,354	3,251,245	34.23
	LINE1	3,740	3,122,723	32.88
	LINE2	485	99,589	1.05
	L3/CR1	69	12,296	0.13
	RTE	55	15,775	0.17
LTR elements		1,641	792,419	8.34
	ERVL	331	177,863	1.87
	ERVL-MaLRs	411	158,016	1.66
	ERV_classI	671	404,210	4.26
	ERV_classII	209	48,569	0.51
DNA elements		888	194,939	2.05
	hAT-Charlie	540	100,870	1.06
	TcMar-Tigger	199	68,973	0.73
Unclassified		14	2,249	0.02
**Total interspersed repeats**			**4,695,007**	**49.43**
Small RNA		1,619	346,963	3.65
Satellites		665	410,951	4.33
Simple repeats		1,127	63,080	0.66
Low complexity		166	8,000	0.08

Supplementary Table 3. Divergence times and phylogenetic topologies of eMSY genes

Evolutionary data on individual eMSY genes showing divergence time with 95% confidence intervals (CI), percent sequence divergence from gametolog/paralog, and phylogenetic pattern; genes that tested positive for gene conversion are denoted "GC" in the topology column.

Gene symbol	Divergence; MYA (95% CI)	% divergence	Topology
		**X-Y ancestral gametologs**	
AMELY	61.5 (55.7-69.2)	8.3	broadly polyphyletic, GC
ANOS1Y	49.2 (28.7-63.4)	5.6	polyphyletic, 2 events, GC
AP1S2Y	134.5 (103.6-171.4)	9.1	monophyletic
BCORY	116.5 (106.3-129.4)	17.9	monophyletic
CUL4BY	142.6 (128.7-155.5)	34.0	monophyletic
DDX3Y	133.7 (117.4-149.3)	9.7	monophyletic
EIF1AY	129.1 (117.1-142.6)	9.9	monophyletic
EIF2S3Y	119.0 (105.1-136.8)	10.6	monophyletic
HSFY[1]	~180	n.a.	monophyletic
KDM5D	133.7 (119.2-147.3)	16.5	monophyletic
NLGN4Y	4.4 (4.1-4.5)	3.9	polyphyletic, 2 events, GC
OFD1Y	4.3 (4.0-4.5)	3.1	broadly polyphyletic
RBMY[1]	~180	n.a	monophyletic
SHROOM2Y	37.5 (25.9-45.2)	8.8	monophyletic
SRY[1]	~180	n.a.	monophyletic
STSY	32.6 (19.8-43.5)	9.0	polyphyletic, 2 events, GC
SYPY	115.7 (107.5-140.6)	41.9	monophyletic
TAB3Y	123.5 (107.5-140.6)	35.5	monophyletic
TBL1Y	35.1 (24.5-47.8)	4.9	broadly polyphyletic, GC
TMSB4Y	4.5 (4.4-4.5)	11.3	polyphyletic, 2 events, GC
TSPY[1]	~180	n.a.	monophyletic
TXLNGY	114.0 (102.0-132.0)	15.7	monophyletic
UBA1Y	137.9 (116.8-159.7)	14.7	monophyletic
USP9Y	137.5 (122.2-154.6)	12.2	monophyletic
UTY	128.4 (112.9-145.2)	10.1	monophyletic
WWC3Y	56.9 (39.4-69.0)	16.9	monophyletic
ZFY	126.9 (113.3-143.3)	17.2	monophyletic, GC
ZRSR2Y	53.0 (27.1-64.8)	16.6	polyphyletic, 2 events, GC
		**PAR transposed**	
ARSFY	2.3 (1.3-3.4)	0.9	monophyletic, GC
ARSHY	4.3 (4.0-4.5)	1.8	monophyletic, GC
		**Autosomal transposed**	
ATP6V0CY	23.9 (14.9-34.7)	4.6	monophyletic
EIF3CY	4.0 (3.4-4.4)	0.9	monophyletic, GC
HSPA1LY	20.1 (11.2-29.8)	3.3	monophyletic
HTRA3Y	4.1 (4.1-4.5)	2.2	monophyletic
MYL9Y	3.8 (1.6-4.5)	0.7	monophyletic, GC
OR8J2Y	65.6 (55.4-75.6)	22.1	monophyletic
OR8K3Y	90.6 (83.0-95.7)	20.1	monophyletic
RPS3AY	23.2 (13.6-34.5)	3.3	monophyletic
SH3TC1Y	3.3 (2.9-3.7)	1.7	monophyletic, GC
TIGD1Y	86.4 (83.8-97.9)	28.5	monophyletic
XKR3Y	9.2 (5.2-16.2)	2.6	polyphyletic, 2 events, GC

[1]available sequences of X and Y paralogs are not sufficiently alignable for divergence time analysis and therefore approximate dates are provided based on Bellott et al. 2014 [11].

Supplementary Table 4: Horse, donkey and mule testis RNAseq statistics

	Sample ID					
	**EAS13**	**EAS18**	**H357**	**H358**	**H383**	**H452**
**Species**	donkey	donkey	mule	mule	horse	horse
**Breed**	Miniature	Miniature	n/a	n/a	American Quarter Horse	Thoroughbred
**Age, years**	3	8	4	3	3	4
**Read Length**	100	100	100	100	100	100
**Ends**	2	2	2	2	2	2
**Total Reads**	87,795,658	87,008,992	75,190,048	74,825,766	78,698,836	83,136,092
**Index %**	15	14.86	12.85	12.79	13.44	14.2
**Passed Filter Reads (PF)**	82,320,998	81,241,658	70,374,154	69,608,268	73,410,388	78,206,040
**Yield (Mb)**	8232	8124	7037	6961	7341	7821
**%PF**	93.76	93.37	93.6	93.03	93.28	94.07
**%Q30 (PF)**	93.5	93.05	93.38	93.05	92.59	93.35
**Mean QScore (PF)**	36.21	36.04	36.21	36.13	35.9	36.17
**%Perfect Index Match**	99.36	99.13	98.94	99.37	98.8	95.58

Supplementary Table 5: Evolutionary strata and divergence estimates of horse gametologs

Divergence estimates of synonymous sites (Ks) between equine gametologs; CI – confidence interval.

			**95% CI**	
**Gene ID**	**Stratum**	**Ks**	**Lower**	**Upper**
HSFY	1	1.968	0.570	3.365
SRY	1	1.811	0.000	7.457
RBMY1	1	0.478	0.135	0.821
RBMY2	1	0.478	0.135	0.821
CUL4BY	1	0.667	0.375	0.959
TSPY	2/3	0.731	0.000	9.725
KDM5D	2/3	0.461	0.395	0.528
SYPY	2/3	0.594	0.067	1.121
UBA1Y	2/3	0.298	0.242	0.354
UTY	2/3	0.243	0.203	0.282
DDX3Y	2/3	0.342	0.254	0.430
USP9Y	2/3	0.317	0.000	0.724
BCORY	2/3	0.403	0.354	0.451
TAB3Y	2/3	0.362	0.092	0.632
ZFY	2/3	0.163	0.119	0.206
EIF2S3Y	2/3	0.320	0.203	0.436
EIF1AY	2/3	0.300	0.116	0.484
TXLNGY	2/3	0.306	0.223	0.388
AP1S2Y	2/3	0.290	0.128	0.452
ZRSR2Y	2/3	0.202	0.103	0.300
OFD1Y	2/3	0.016	0.008	0.025
TMSB4Y	2/3	0.272	0.046	0.498
AMELY	2/3	0.130	0.049	0.212
WWC3Y	2/3	0.261	0.208	0.315
SHROOM2Y	4	0.094	0.037	0.151
TBL1Y	4	0.099	0.069	0.128
ANOS1Y	4	0.188	0.107	0.268
STSY	4	0.141	0.104	0.178
NLGN4Y	4	0.124	0.092	0.156

Supplementary Table 6: Details of the *Parascaris* samples

The *Parascaris* samples were used to validate putative horizontal transfer by PCR (see Fig. 6 and Supplementary Fig. 8 for more details).

**ID**	**Develop. stage**	**Processing before DNA isolation**	**Worm sex**	**Horse (host) ID**	**Horse (host) sex**
A0.1	adult	flash frozen in LN2	male	#260-15	female
A0.3	adult	flash frozen in LN2	male	#260-15	female
A0.4	adult	flash frozen in LN2	male	#260-15	female
A1	adult	Transported to the lab in a sub-section of small intestine in a 37°C water bath; removed from intestine and flash frozen in LN2	male	#17-C39	male
A1.1	adult	Same as above; 2nd DNA isolation	male	#17-C39	male
A3	adult	Collected and removed from small intestine; transport in RPMI 1680 in a 37°C water bath; cultivated in RPMI 1680 at 37°C for 48h; snap frozen in LN2	male	#17-C39	male
L4.0	L4 larva	flash frozen in LN2	n/a	n/a	n/a
L4.1	L4 larva	flash frozen in LN2	n/a	n/a	n/a
E0	eggs; external stage	unembryonated eggs isolated from the uterus of a dissected female worm	Female	n/a	n/a
E0.1	eggs, external stage	unembryonated eggs isolated from the uterus of a dissected female worm (a different female worm than for E0)	female	n/a	n/a
BW	body wall	dissected from 3 adult worms	males	n/a	male
I	intestine	dissected from 3 adult worms	males	n/a	male
G	gonads	dissected from 3 adult worms	males	n/a	male

Supplementary Figure 1

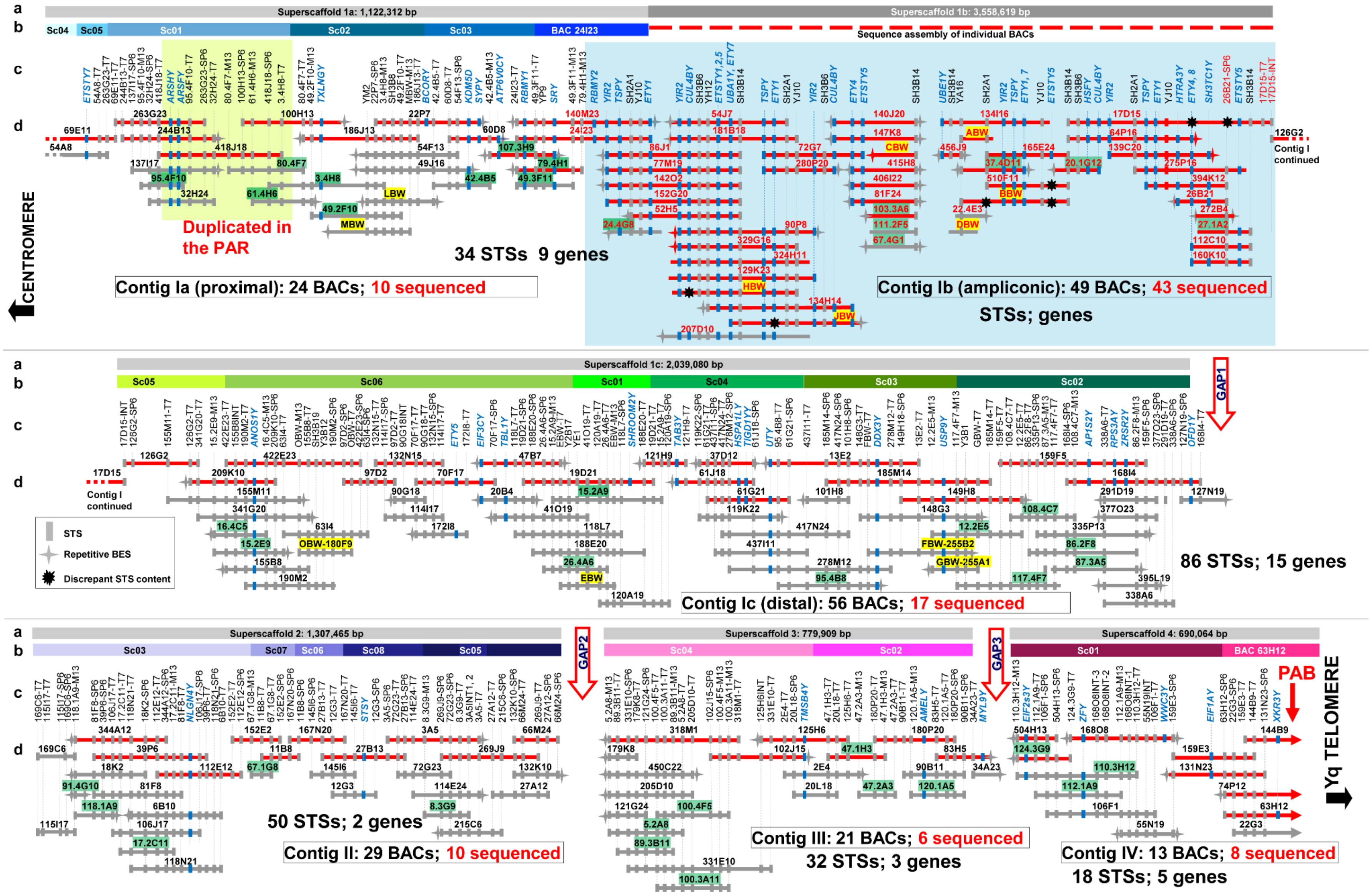

Supplementary Fig. 1: BAC tiling path map for horse MSY sequencing and assembly. a-b Horse MSY sequence assembly scaffolds and superscaffolds (details in Supplementary Table 1); **c** Linear order of 265 genes, ESTs and STS markers to support the order and overlaps of BAC clones (marker details are in Supplementary Data 4); genes are in blue bold italic font; STS markers in regular font; **d** The tiling path of 192 partially overlapping BAC clones of which 139 originated from CHORI-241 BAC library (BAC IDs in regular black or red font); 41 from TAMU BAC library (green highlight), and 12 from INRA BAC library (yellow highlight). The tiling path clones were arranged into four contigs (I, II, III, IV) leaving 3 gaps for which no clones were found. Contig I was further divided into two single-copy regions – Ia and Ic, and an ampliconic region Ib (blue shade with BAC IDs in red font). The tiling path of ampliconic BACs was arranged provisionally based on a set of selected markers. Light green shaded area in contig Ia denotes the PAR-transposed region in eMSY. Proximally, the BAC tiling path extends into Y heterochromatin and distally into the PAR, thus spanning across the entire eMSY. BAC clones are denoted with colored horizontal bars: in *red*, if sequenced, in *grey*, if not sequenced; STSs are connected with vertical dotted lines to their locations in tiling path BAC clones. The total number of BACs and the number of clones sequenced are shown below each contig.

Supplementary Figure 2

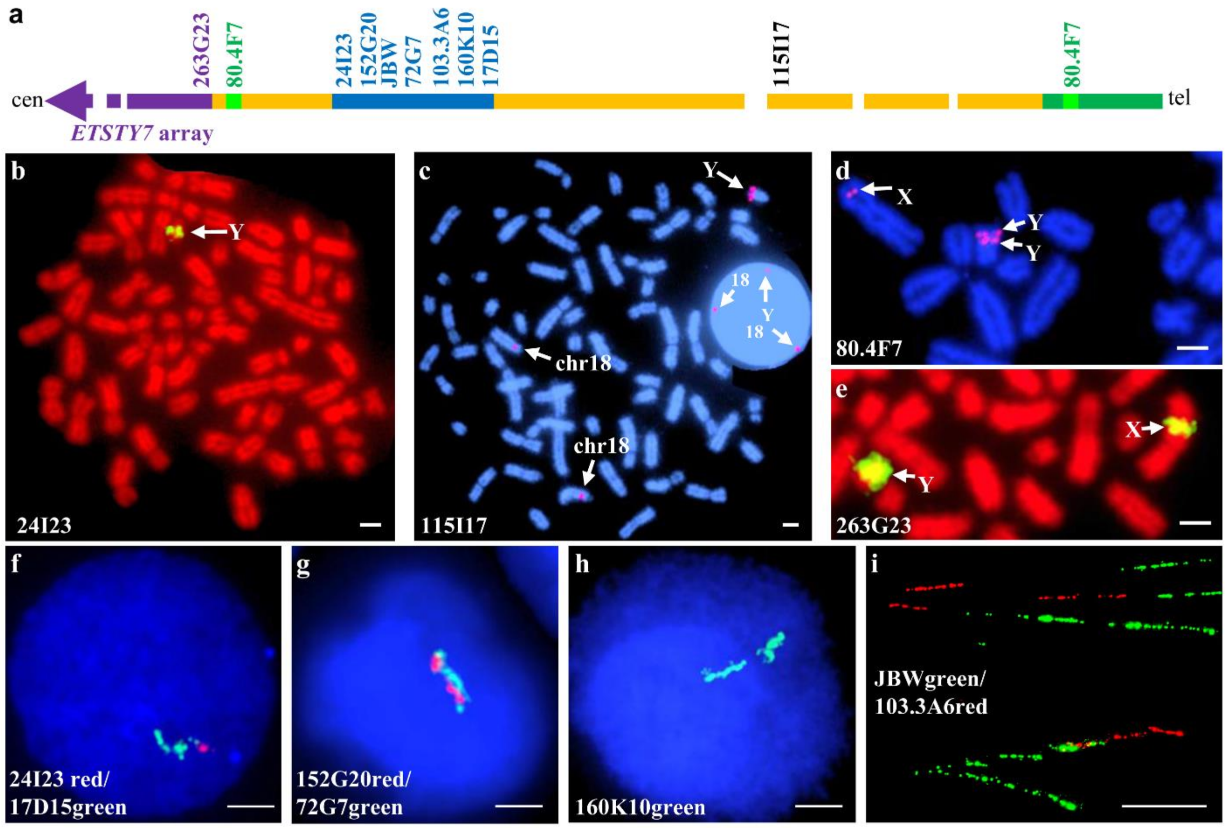

Supplementary Fig. 2: Validation of tiling path BACs by FISH. a Schematic of horse MSY BAC tiling path map showing the location of BACs corresponding to FISH images b-i; single-copy regions are shown in yellow; ampliconic regions in blue; *ETSTY7* array in purple; the pseudoautosomal region in green, and PAR-transposed region in eMSY in light green; **b-e** Examples of FISH validation: **b** metaphase showing Y specificity of BAC 24I23; **c** metaphase and interphase showing BAC 115I17 sequence shared between MSY and chr18; **d** metaphase showing PAR transposition in eMSY, and **e** massive amplification of *ETSTY7* arrays in Y and Xq17-q21 heterochromatin; **f-i** Evaluation of BAC copy numbers by FISH in interphase chromosomes and DNA fibers: **f** multi-copy BACs 24I23 (red) and 17D15 (green) flanking the main ampliconic region (see a); **g** dual-color FISH with BACs 152G20 (~ 4 copies) and BAC 72G7 (~10 copies); **h** multiple copies of BAC 160K10, and **i** Fiber-FISH illustrating proximity of ampliconic BACs JBW (green) and 103.3A6 (red); Scale bars in **b-h** 1µm; scale bar in **i** (fiber-FISH) 100 kbp.

Supplementary Figure 3

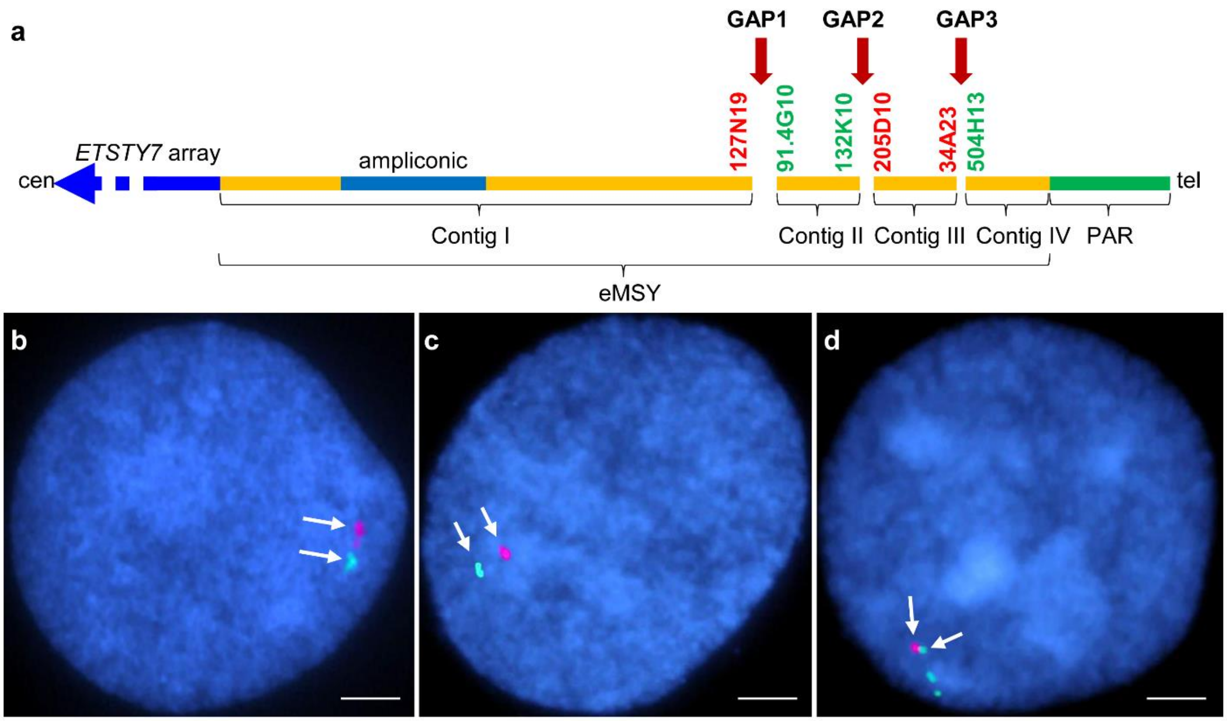

Supplementary Fig. 3: Estimation the size of gaps in eMSY tiling path by FISH. a Schematic of horse MSY BAC tiling path map showing the location of gaps and gap-flanking BACs; **b-d** Images of dual-color interphase FISH with pairs of gap-flanking BACs; **a** GAP1: 127N19 red (contig I) and 91.4G10 green (contig II); **b** GAP2: 132K10 green (contig II) and 205D10 red (contig III); **c** GAP3: 34A23 red (contig III) and 504H13 green (contig IV); Based on the analysis of at least 30 interphase cells per experiment, we estimated the size of GAP1 and GAP2 to be approximately 1 Mbp, and GAP3 to be approximately 500-600 kbp; None of the gaps could be visualized by fiber-FISH, suggesting that all were larger than 500 kbp; Scale bar 1µm.

Supplementary Figure 4

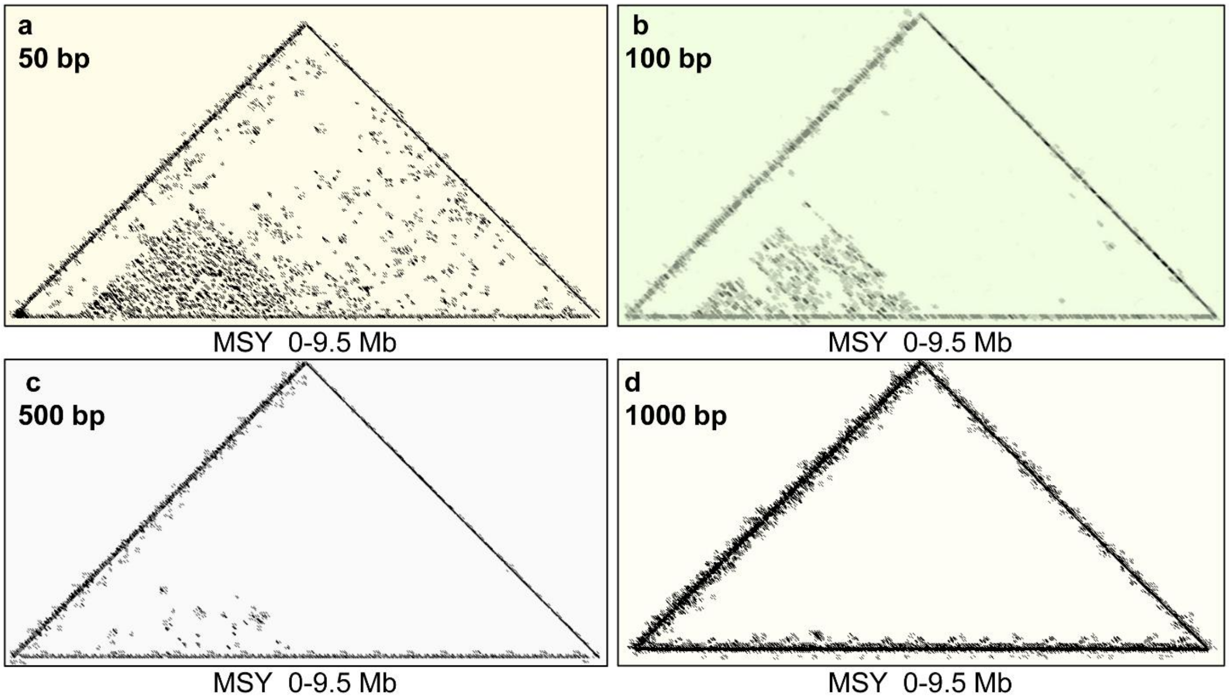

Supplementary Fig. 4: Intra-chromosomal sequence similarity. a-d Triangular dot plots showing the location of 100% identical sequences in horse MSY sequence map 0-9.5 Mbp (x-axis) using sequence motifs (word size) of 50 bp (**a**); 100 bp (**b**); 500 bp (**c**), and 1000 bp (**d**) with a step of 20% of the word size. Note the accumulation of 100% identical sequences specifically in the major ampliconic region at approximately 1-4 Mb in eMSY (seen as a pyramid shape). The decrease in identity observed as word size increases is reflective of the increase in divergence between homologous sequence blocks since the expansion of this ampliconic region.

Supplementary Figure 5

a

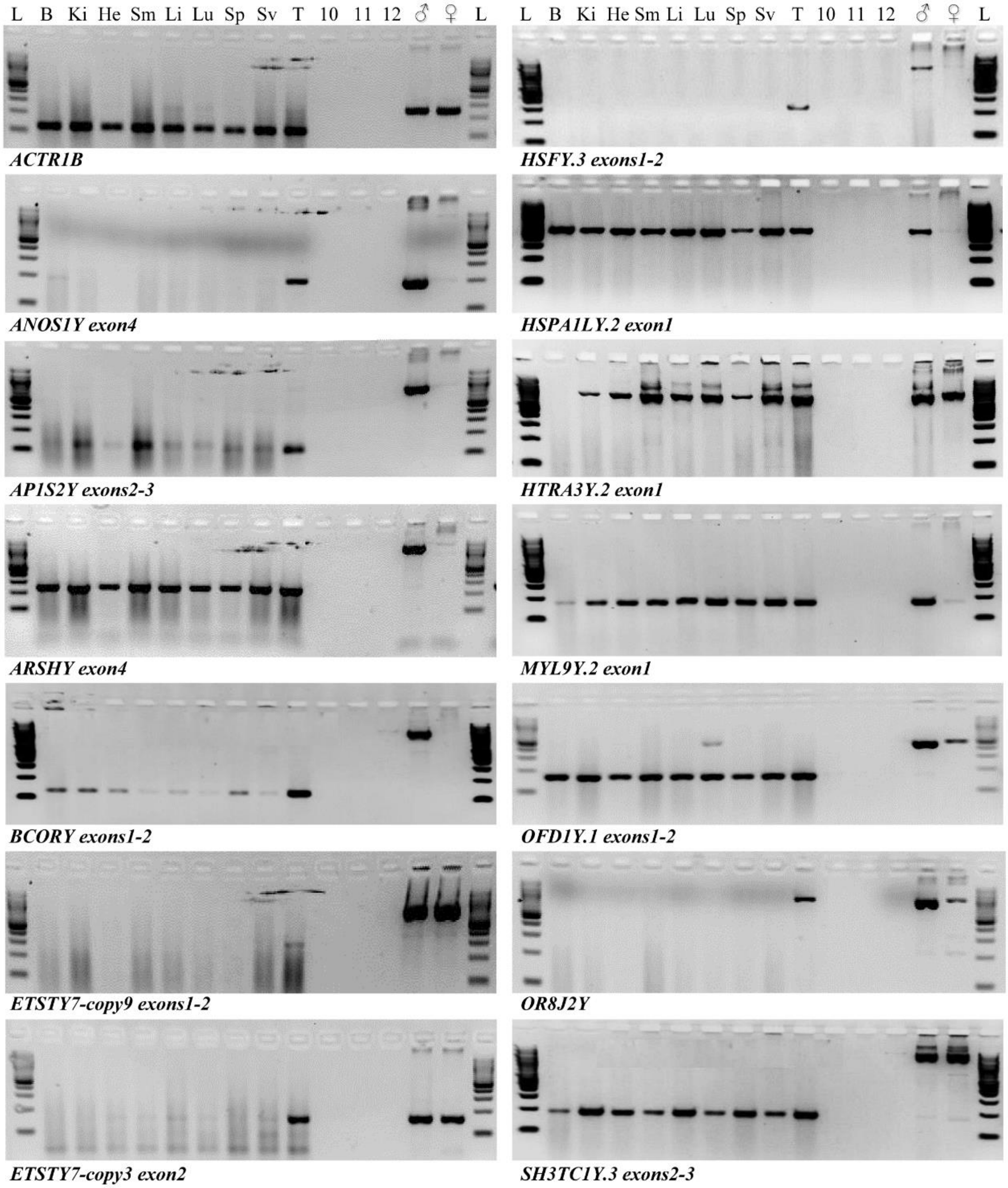

Supplementary Figure 5 continued

a continued

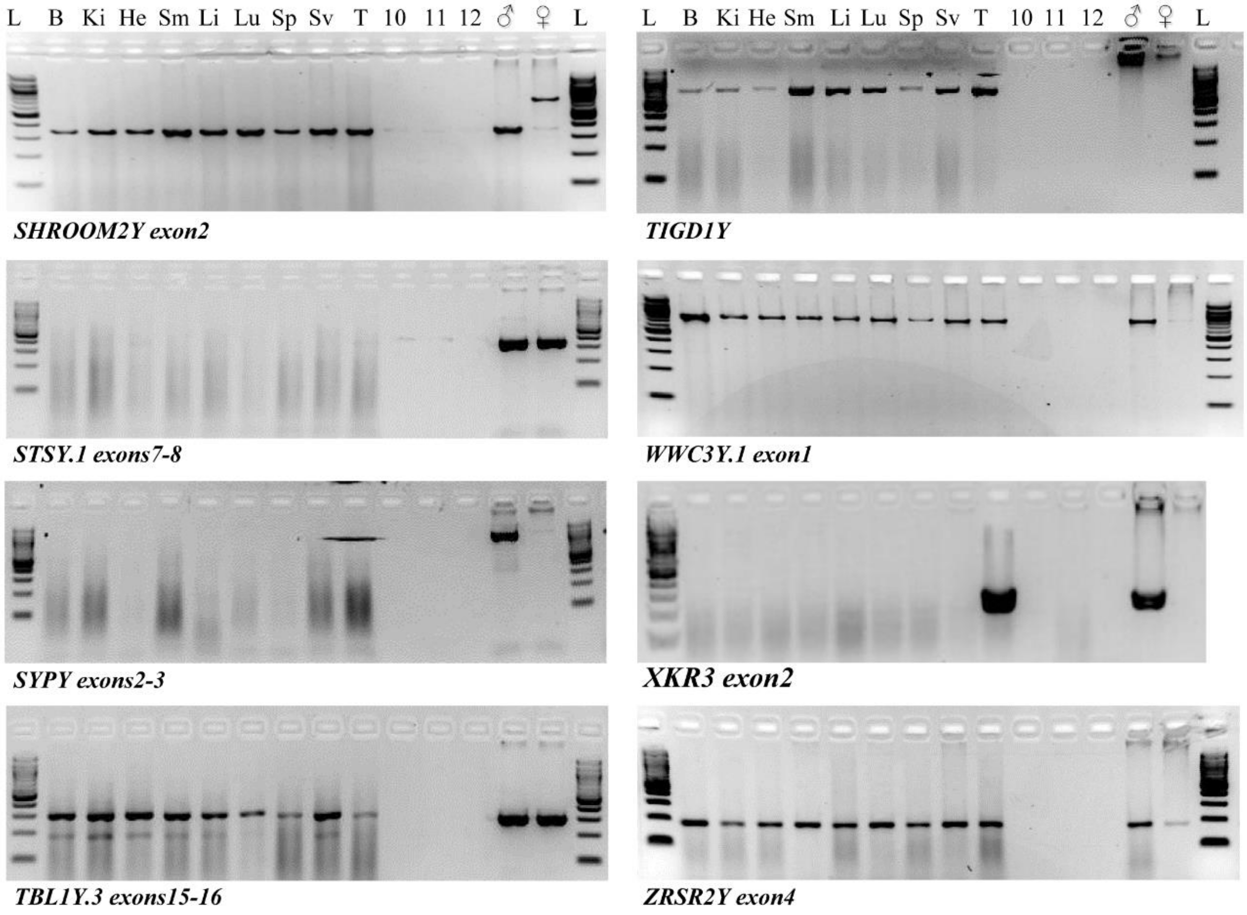

Supplementary Fig. 5: Expression profiles of eMSY genes by reverse transcriptase PCR. a In equine adult tissues: B – brain, Ki – kidney, He – heart, Sm – skeletal muscle, Li – liver, Lu – lung, Sp – spleen, Sv – seminal vesicle, T – testis, 10 – no mRNA control, 11 – no RT control, 12 – no genomic DNA control, ♂ – male genomic DNA control, ♀ – female genomic DNA control; L –100 bp ladder (NEB); shown are only results for newly discovered MSY genes (n=19) not including those published by Paria et al. 2011 [2]. An exception is equine testis-specific transcript on Y 7 (*ETSTY7*, alias *ZNF33b*) which was included to confirm that this highly ampliconic transcript is expressed exclusively in testis. The control housekeeping gene was autosomal *ACTR1B*.

Supplementary Figure 5 continued

b

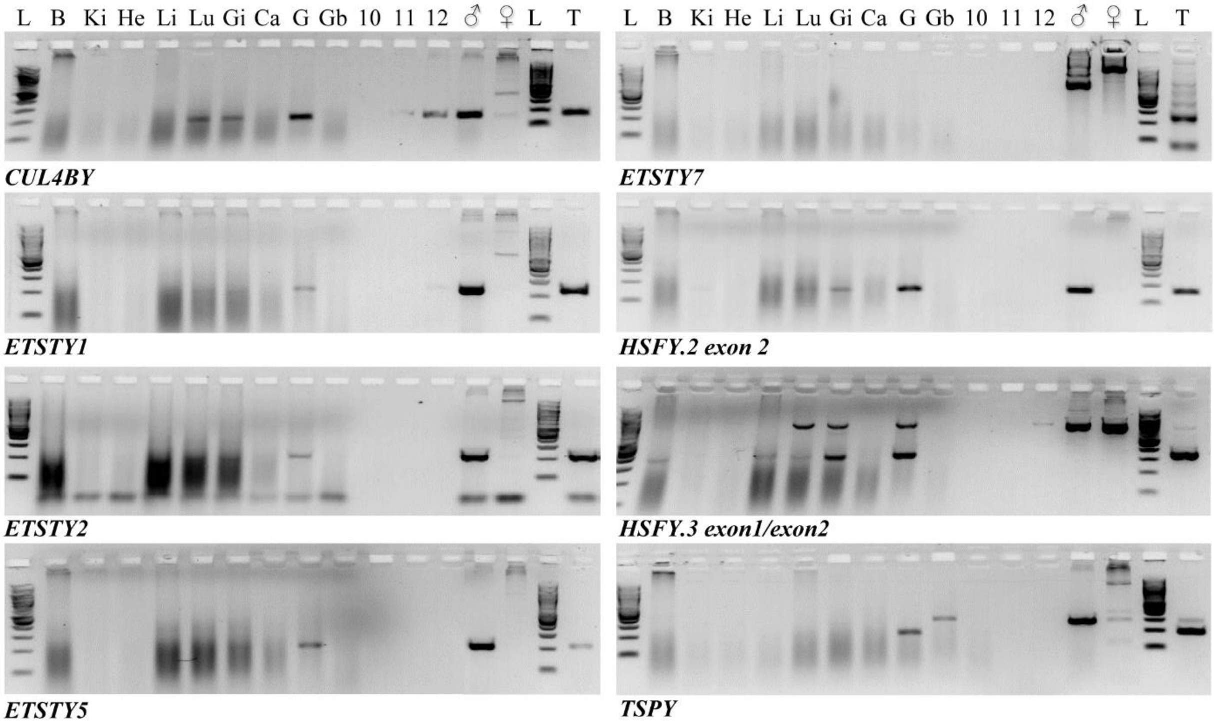

Supplementary Fig. 5: Expression profiles of eMSY genes by reverse transcriptase PCR. b In equine fetal tissues: L –100 bp ladder (NEB), B – brain, Ki – kidney, He – heart, Li – liver, Lu - lung, Gi – Gi-tract, Ca – chorionic allantois, G – gonad, Gb – gubernaculum, 10 – no RT control, 11 – no mRNA template control, 12 – no gDNA control, ♂ – male gDNA control, ♀ – female gDNA control; T – adult testis cDNA control. The figure depicts RT-PCR results with select ampliconic eMSY genes on a tissue panel of a 50 days-old male equine fetus.

Supplementary Figure 6

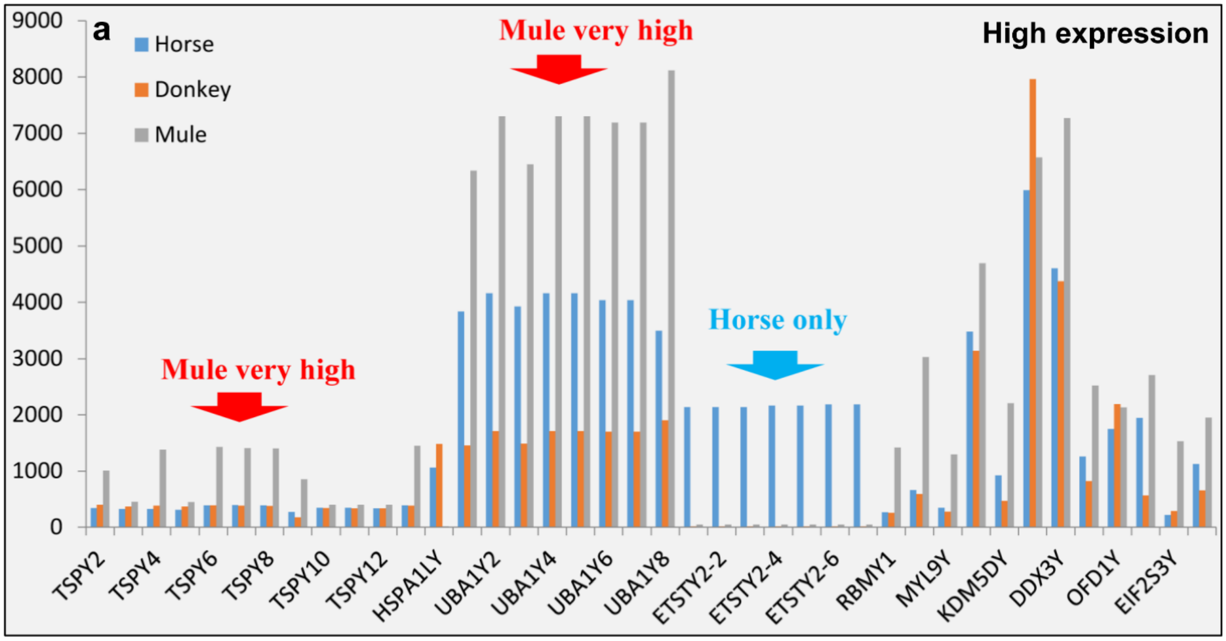

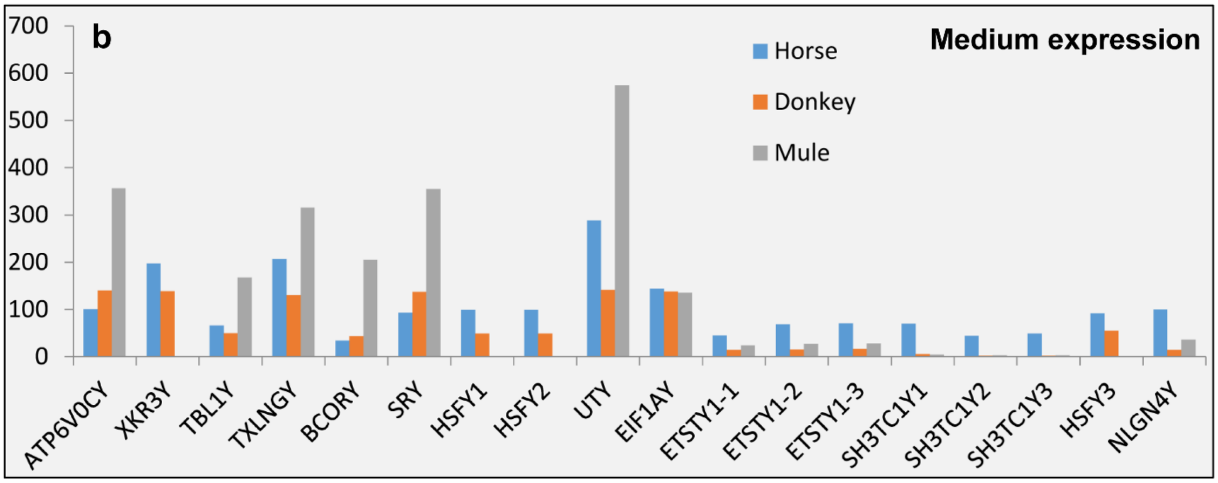

Supplementary Figure 6 continued

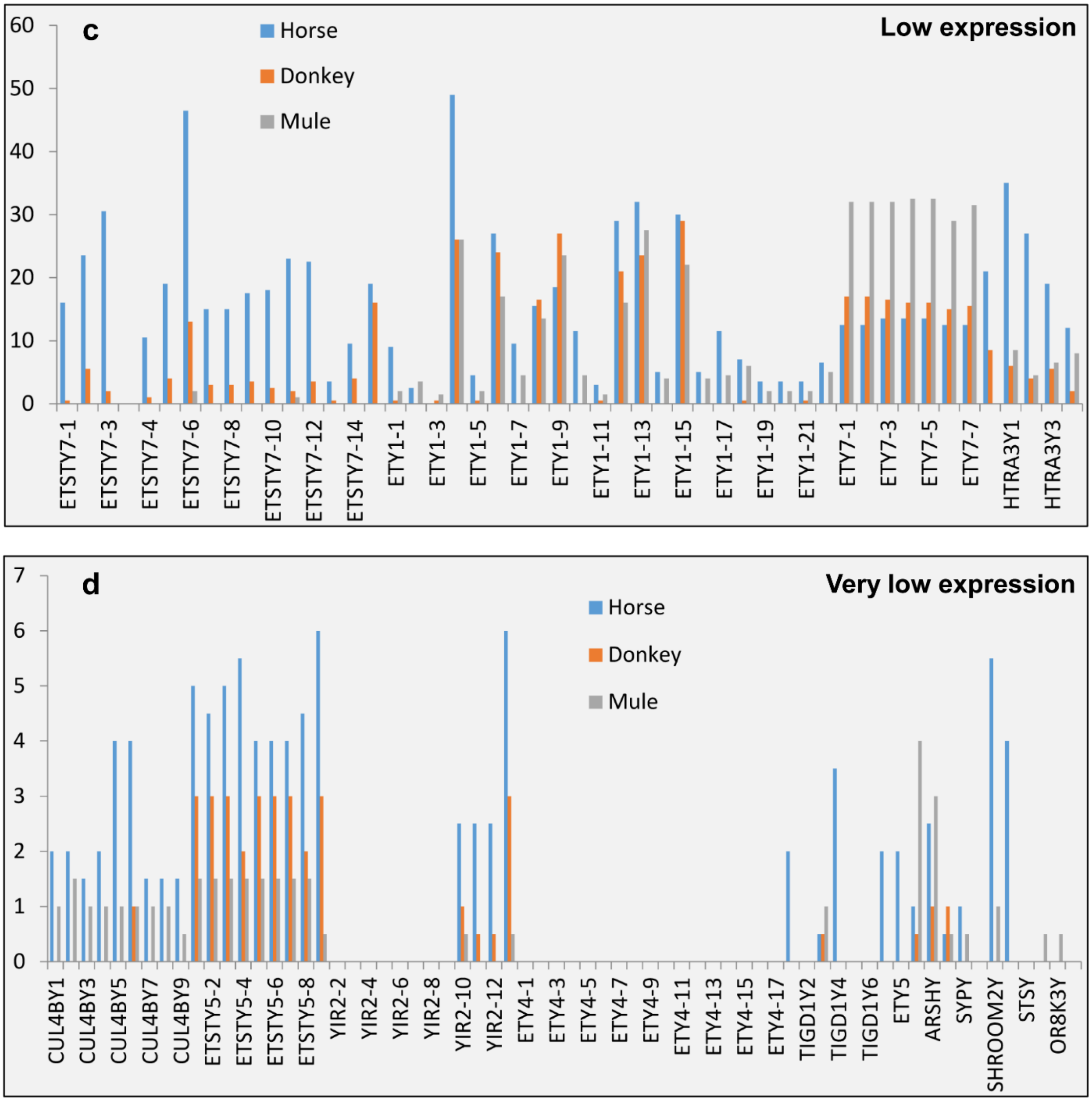

Supplementary Fig. 6: MSY gene expression in horse, donkey and mule testis. Histogram of total exonic read counts per MSY gene for the average of two horses (H383 and H452), two donkeys (EAS13 and EAS18), and two mules (H357 and H358). Due to the high homology of MSY gene families, both ambiguously and unambiguously mapped reads were counted. Comparison of read counts between horse, donkey and mule is a proxy for relative expression of MSY genes in each species or hybrid. We categorized expression into four mutually exclusive groups **a** High abundance; mapped reads > 1000; **b** Moderate abundance; mapped reads > 70; **c** Low abundance; mapped reads > 10, and **d** Very low abundance; remainder of annotated genes (see also Supplementary Data 8).

Supplementary Figure 7

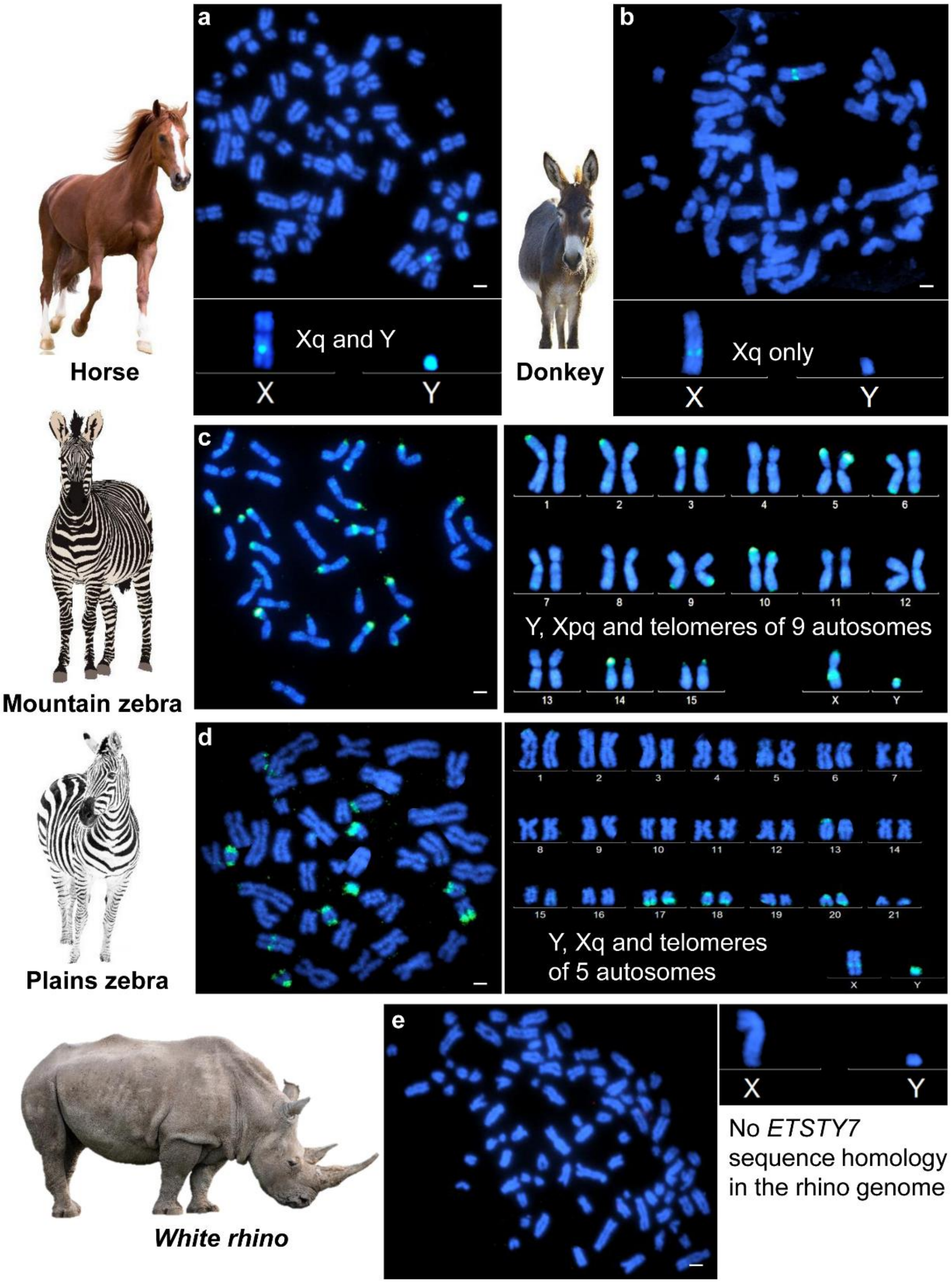

Supplementary Fig. 7: *ETSTY7* FISH analysis in equids and the rhino. For each species, we presented a metaphase image and an arranged karyogram showing chromosomal distribution of *ETSTY7* sequences in **a** horse; **b** donkey; **c** Hartmann's mountain zebra; **d** Plains zebra, and **e** White rhinoceros. *ETSTY7* arrays are present in the genomes of all studied equids, but not in the rhino. Scale bar 1µm. Animal images purchased from Bigstock https://www.bigstockphoto.com/.

Supplementary Figure 8

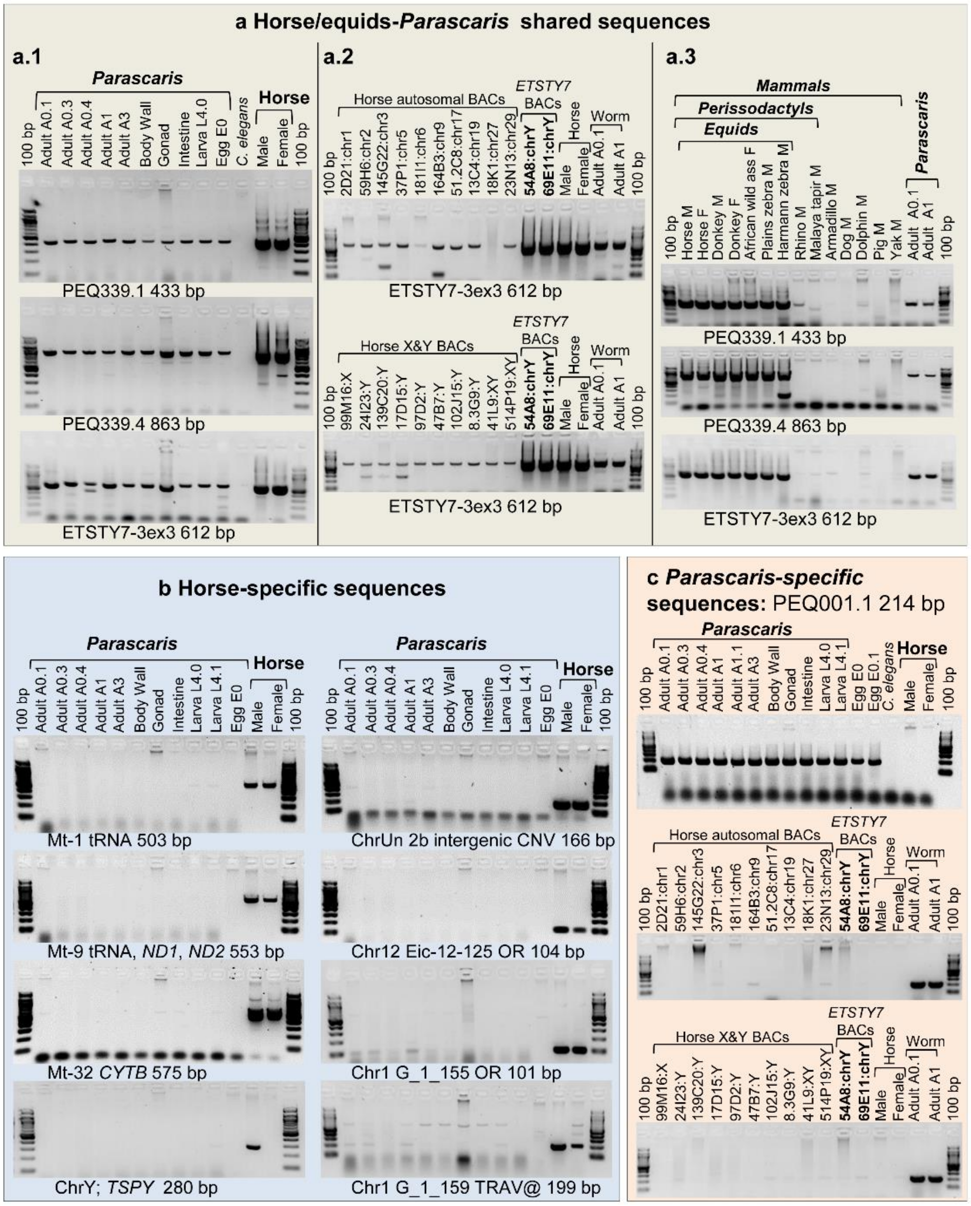

Supplementary Fig. 8. Validation of horizontal transfer. a HT validation with primers designed from horse-parasite shared sequences in horse genome (*ETSTY7*-3ex3) and *Parascaris* genome (PEQ339.1, PEQ339.4) on 3 groups of DNA templates: **a.1** - 13 *Parascaris* individuals, tissues and developmental stages, *C. elegans* as negative invertebrate control, and male and female horses; **a.2** – 10 randomly selected autosomal and 12 X and Y BACs from CHORI-241 and TAMU libraries; BACs 54A8 and 69E11 (bold font) contain 15 copies of *ETSTY7* in eMSY sequence map (Fig. 1 and Supplementary Fig. 1); **a.3** – equids, Perissodactyls and diverse mammals; **b** Tests for horse-to-parasite DNA contamination with primers designed from horse-specific multicopy sequences in mtDNA, autosomes, chrUn and eMSY; **c** Tests for parasite-to-horse DNA contamination with primers designed from *Parascaris*-specific scaffold PEQ_scaffold0000001 (LM462759) and using parasite and horse gDNA and horse BAC DNA as templates.

Supplementary Figure 9

AMELY

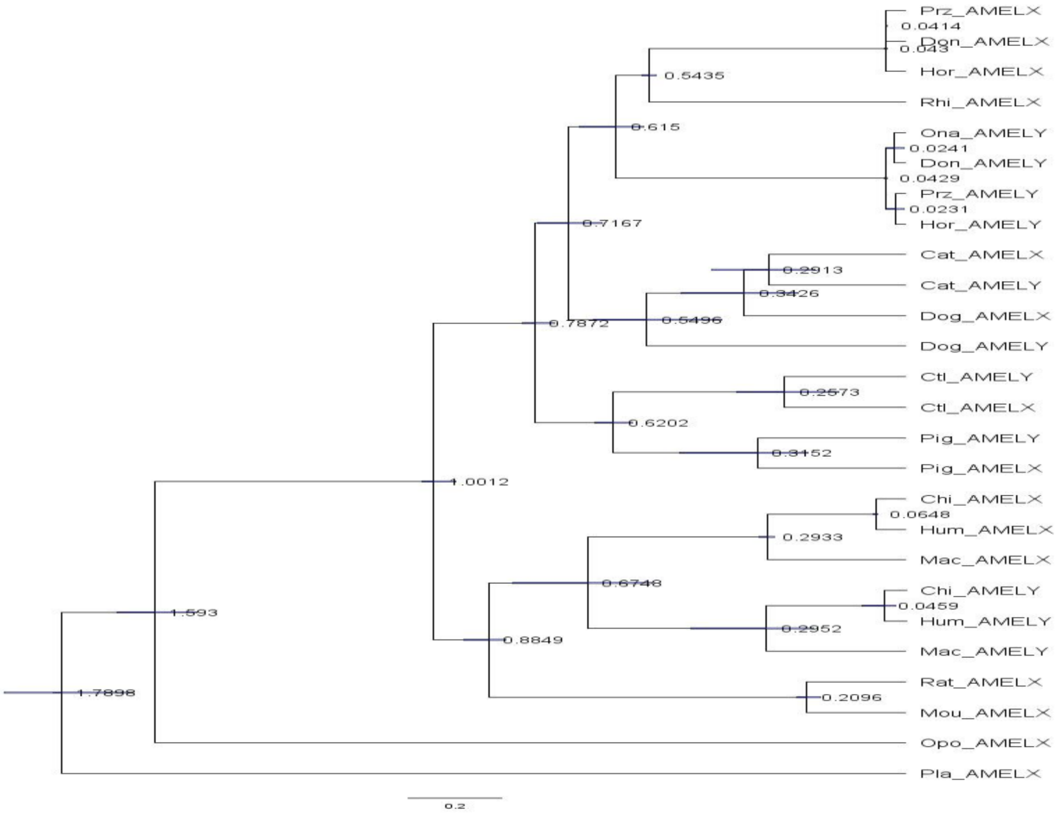

ANOS1Y

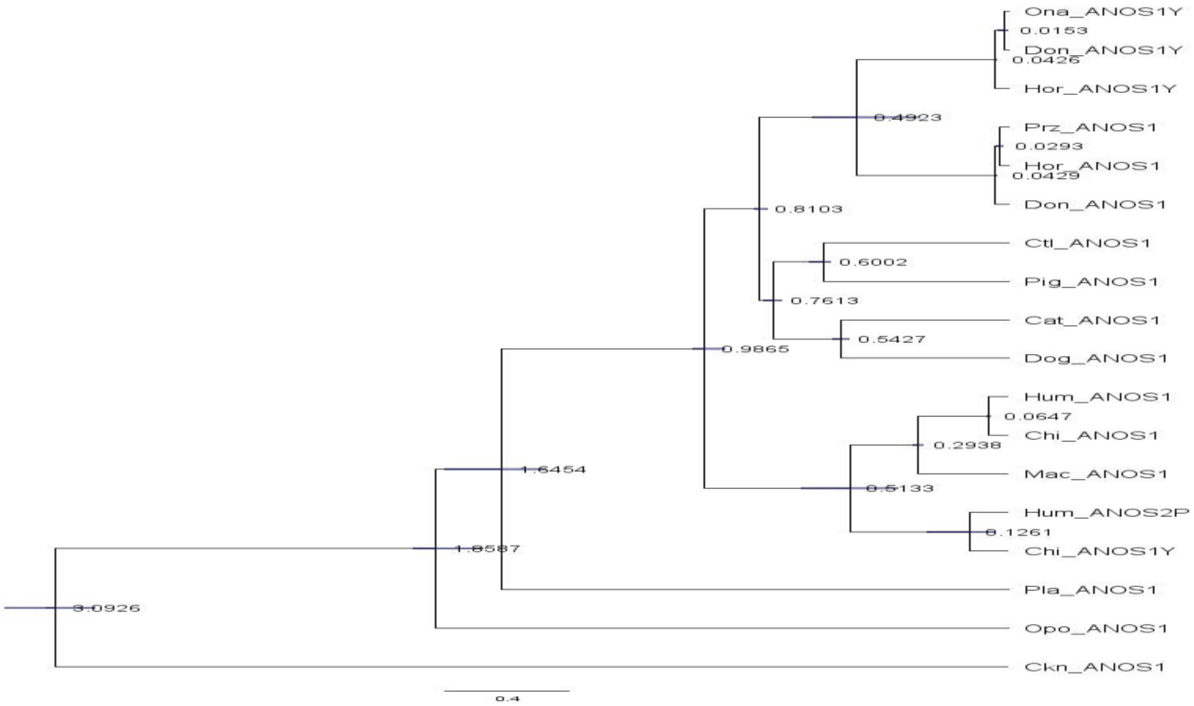

Supplementary Figure 9 continued

AP1S2

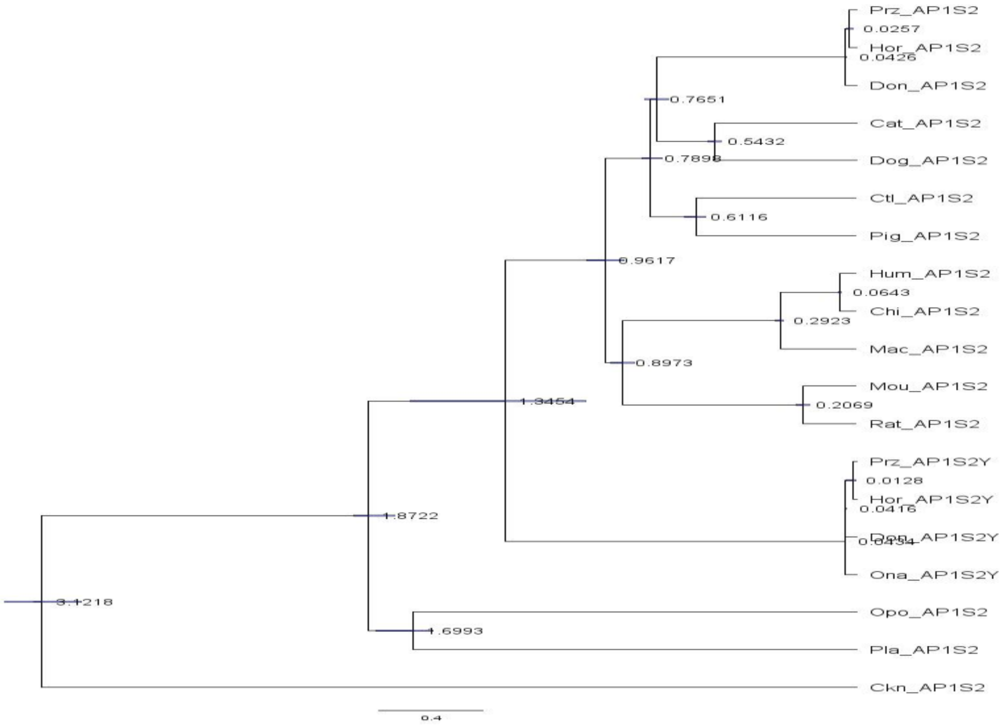

ARSFY

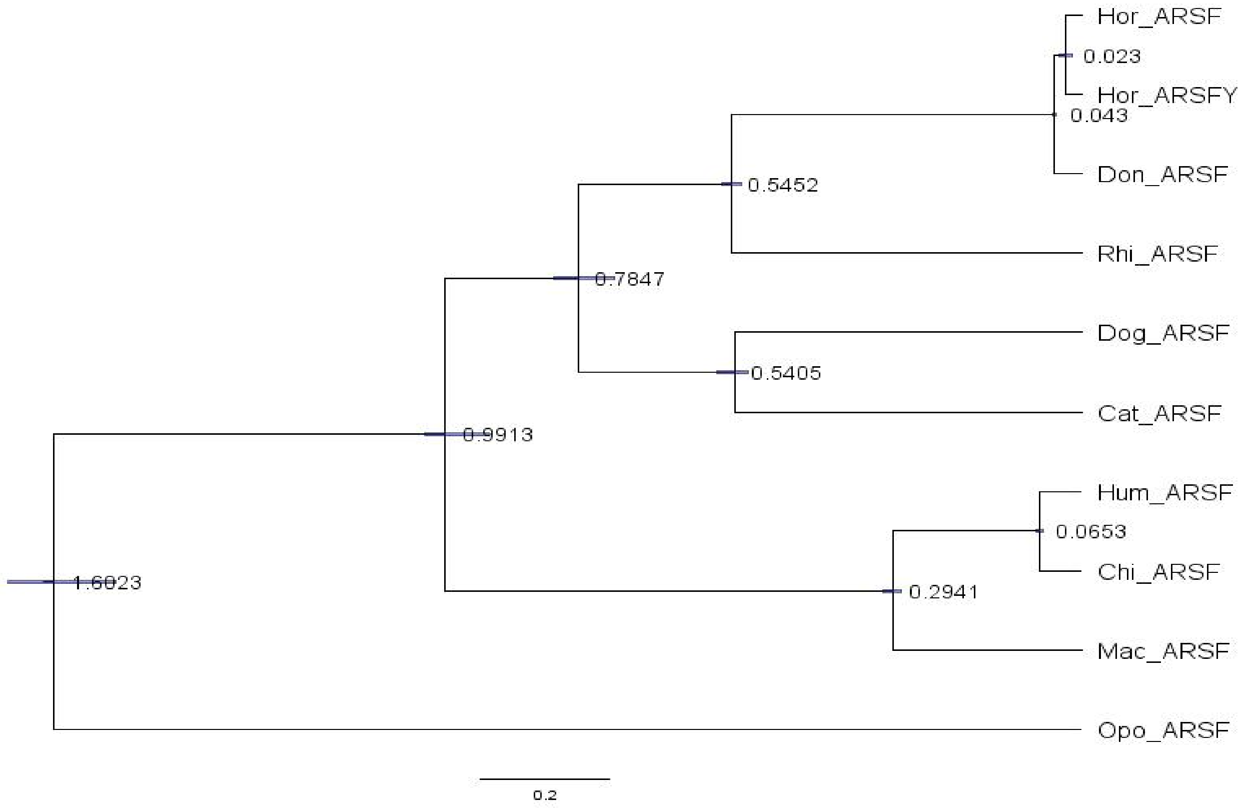

Supplementary Figure 9 continued

ARSHY

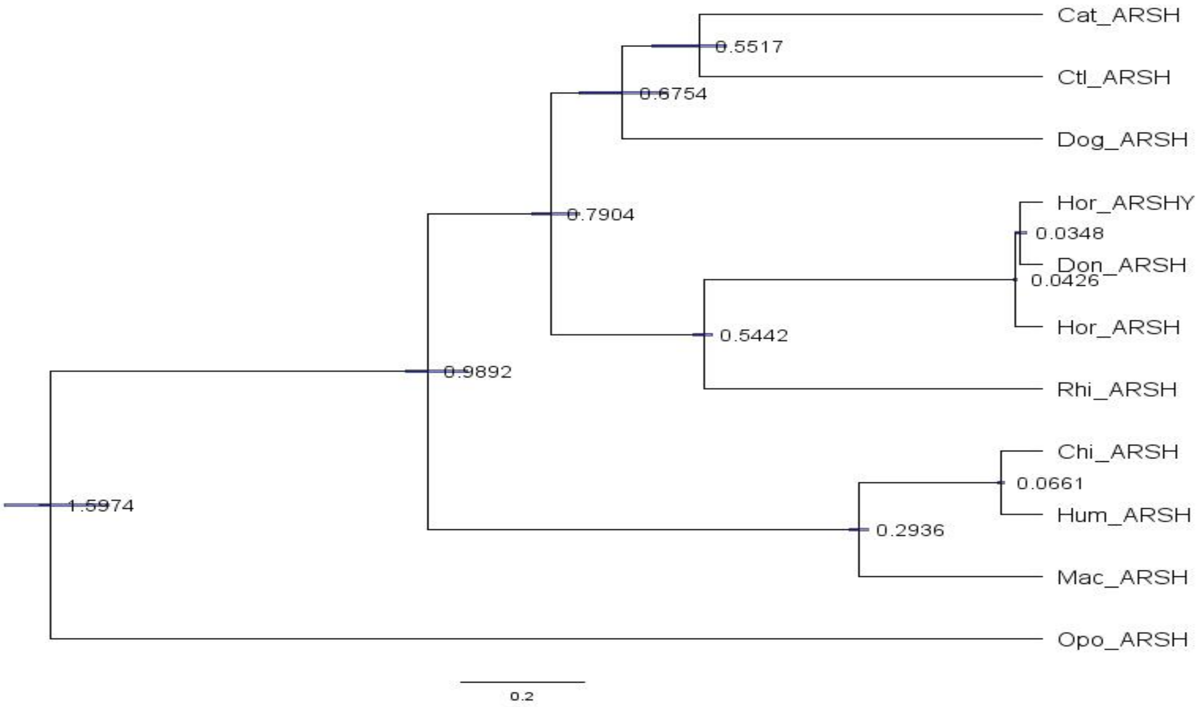

ATPV0CY

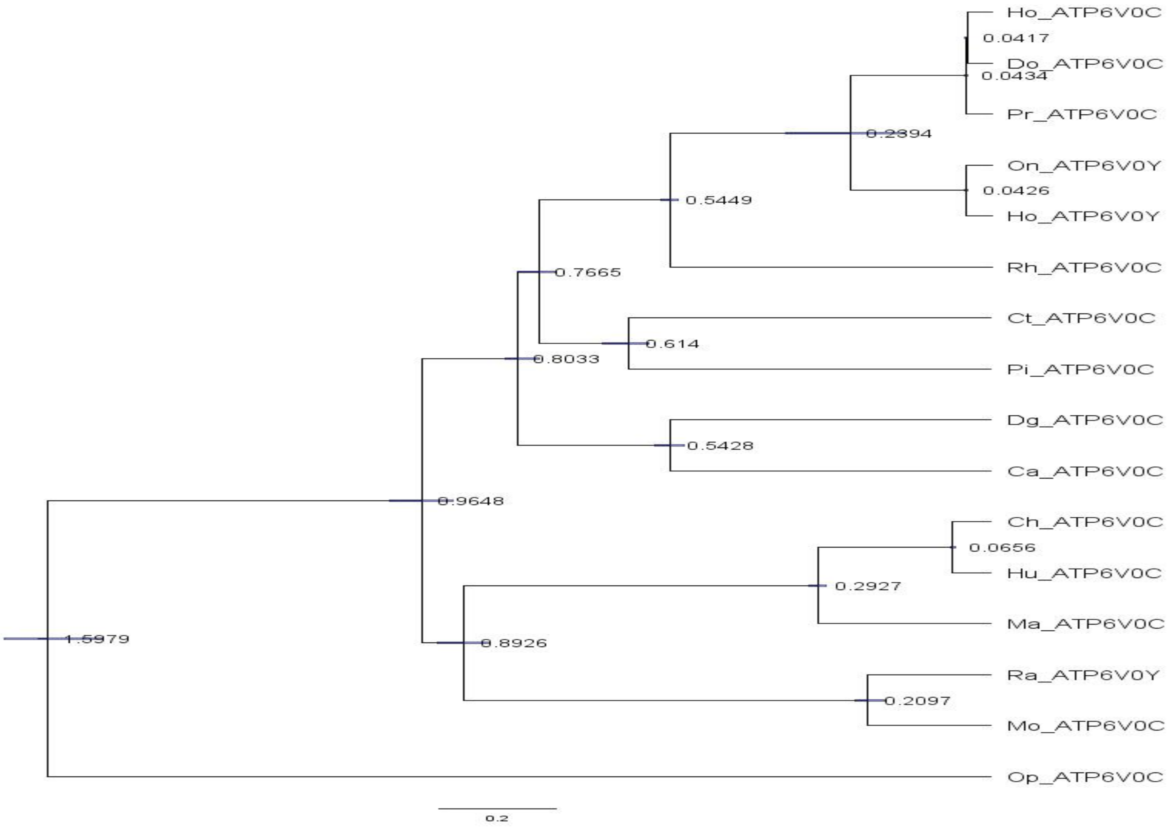

Supplementary Figure 9 continued

BCORY

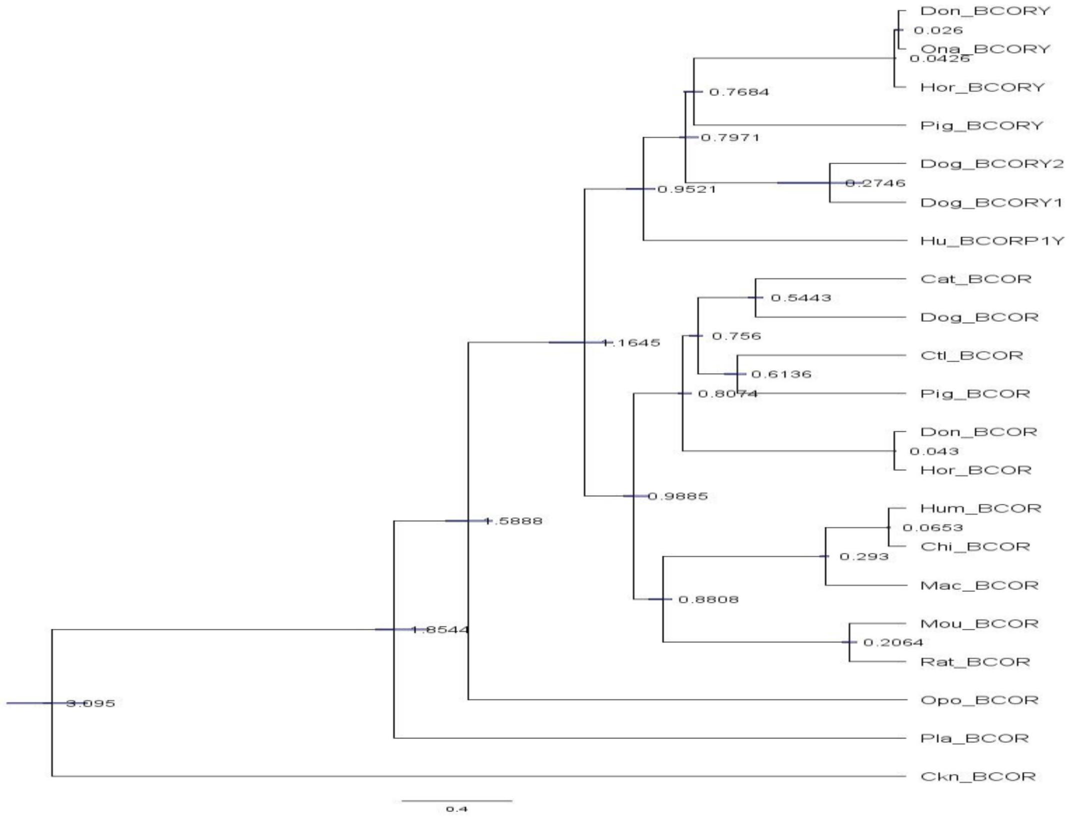

CUL4BY

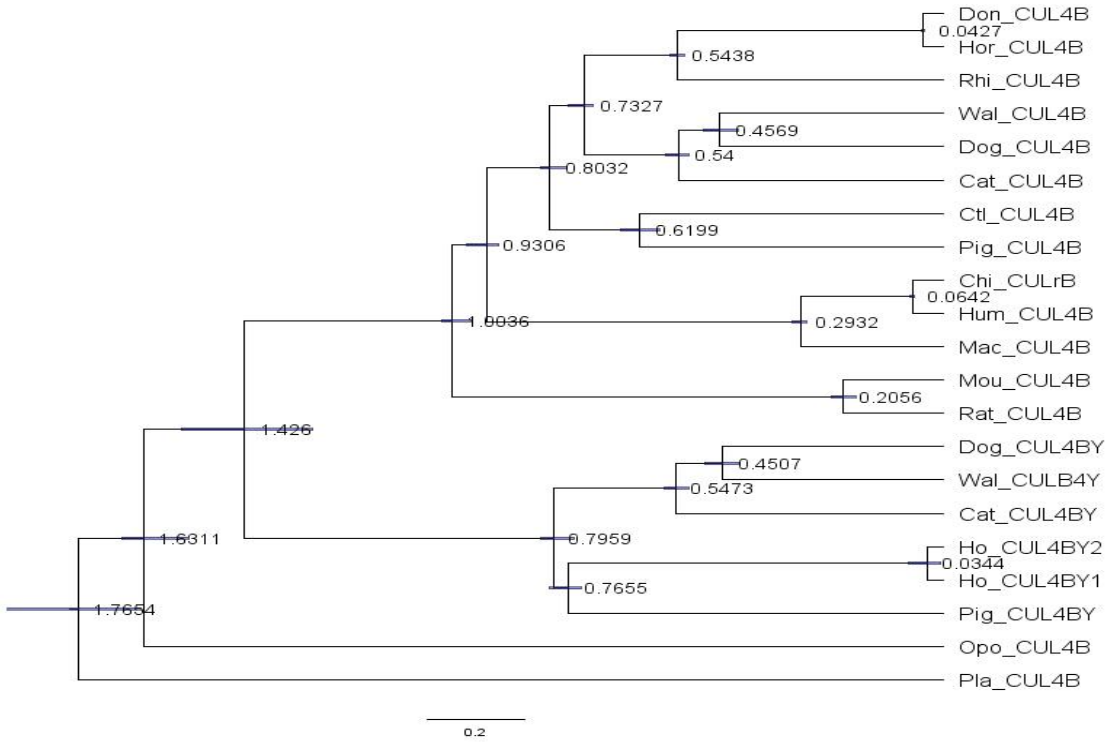

Supplementary Figure 9 continued

DDX3Y

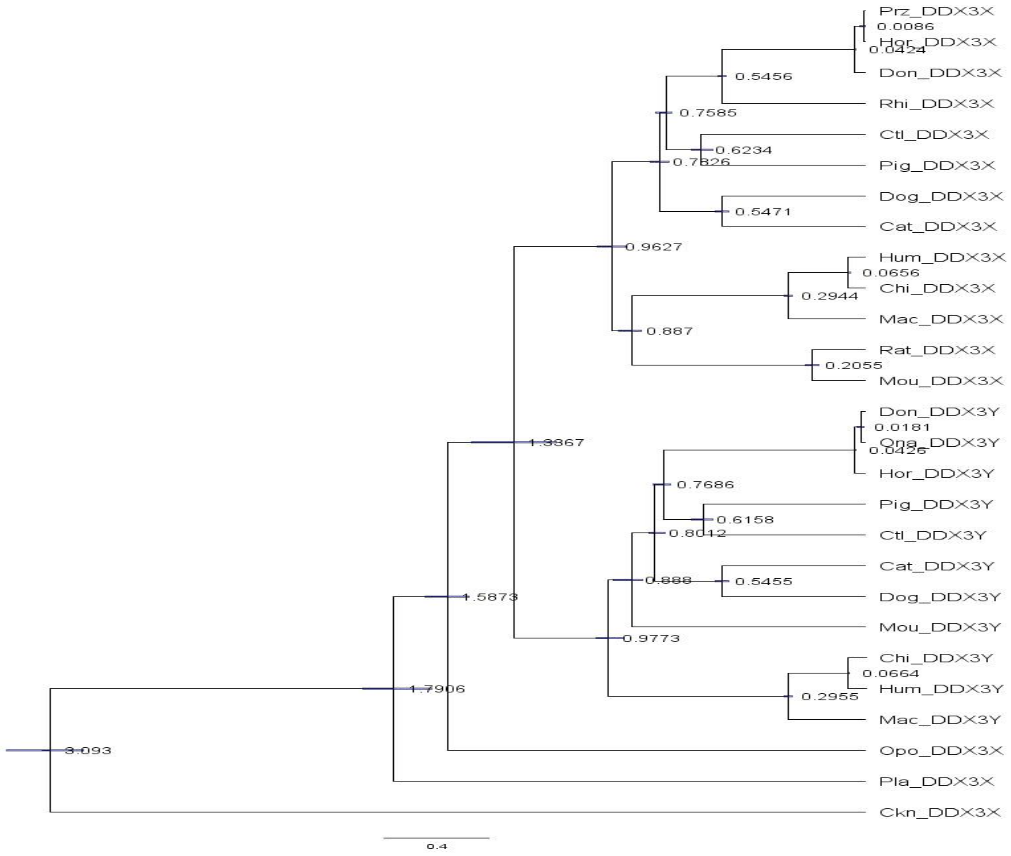

EIF1AY

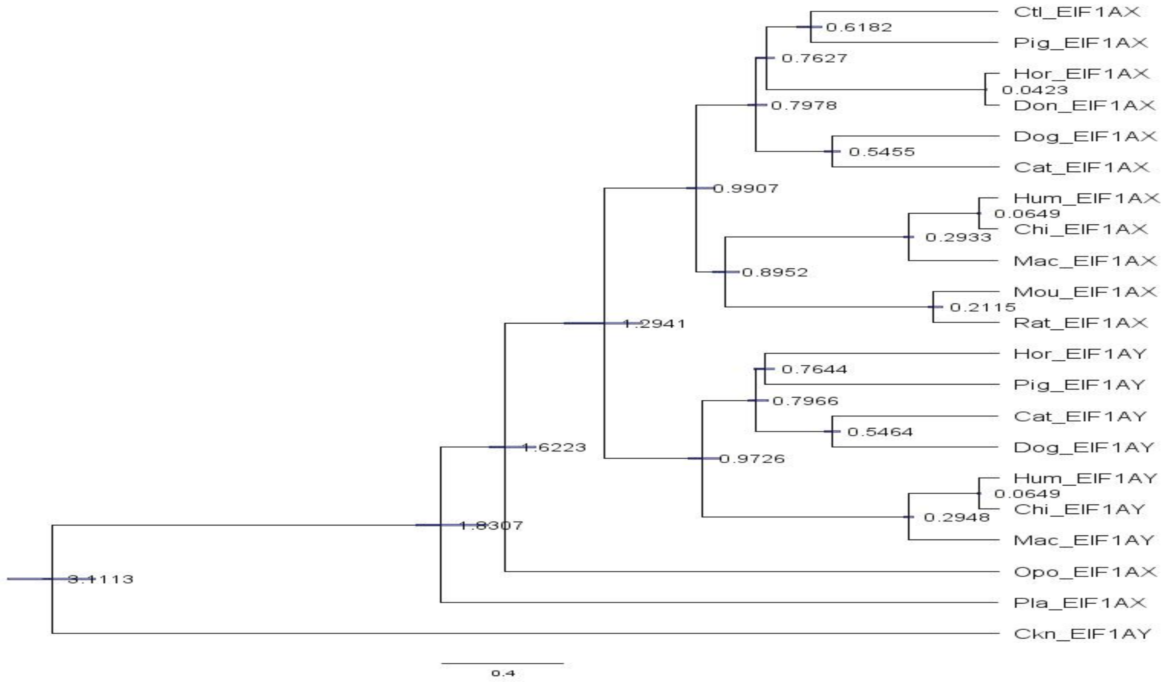

Supplementary Figure 9 continued

EIF2S3Y

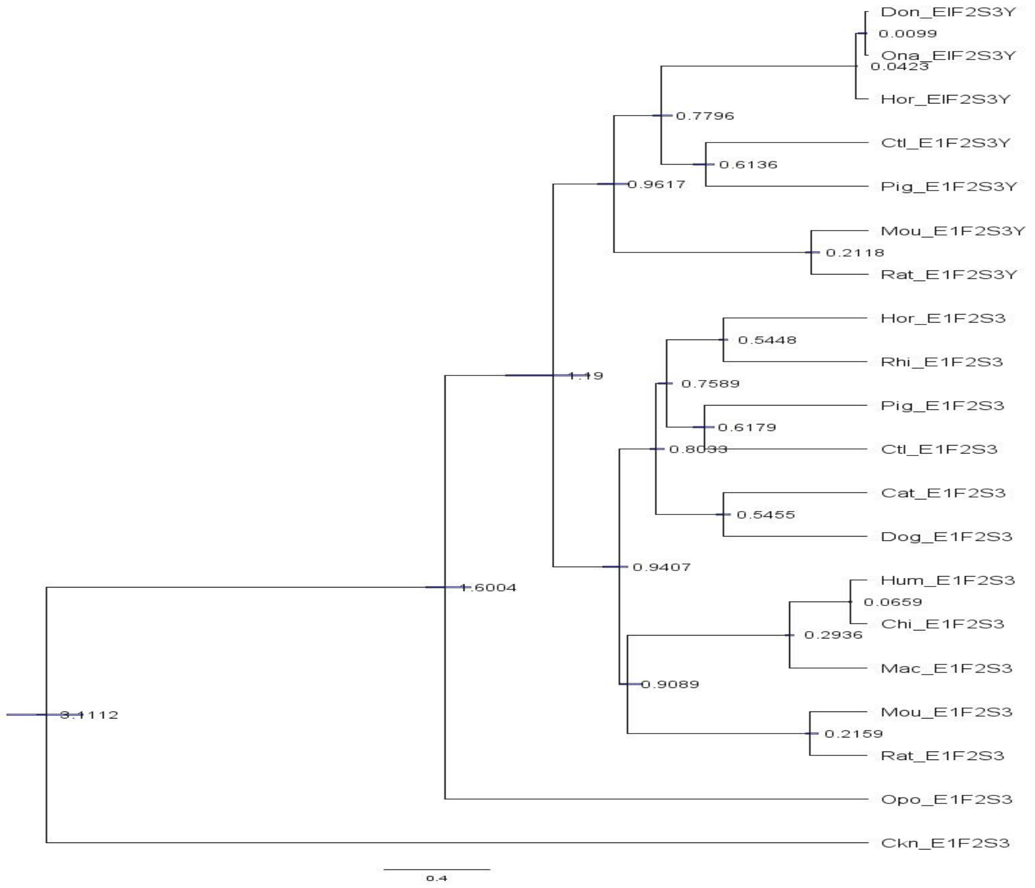

EIF3CY

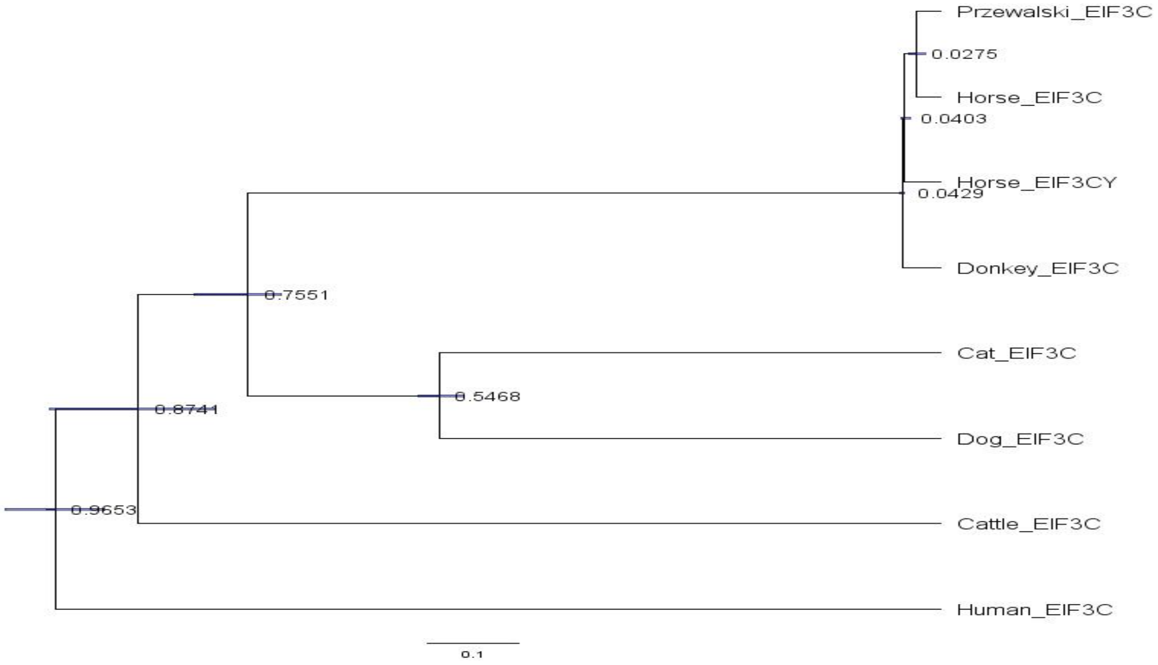

Supplementary Figure 9 continued

HSPA1LY

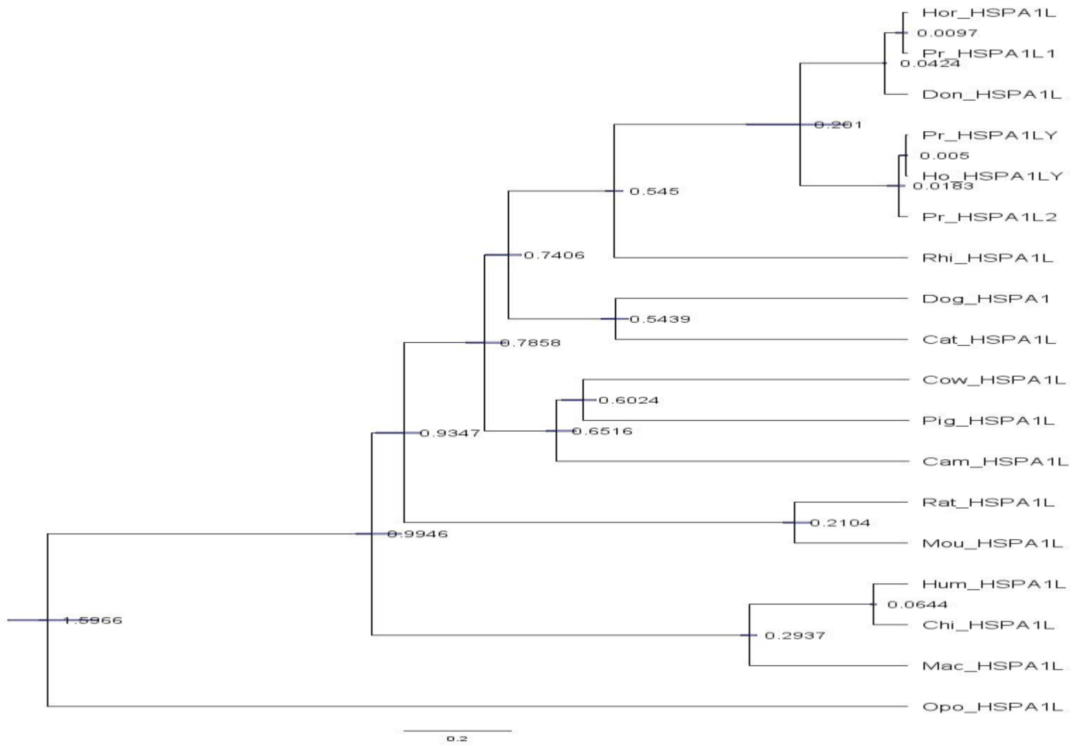

HTRA3Y

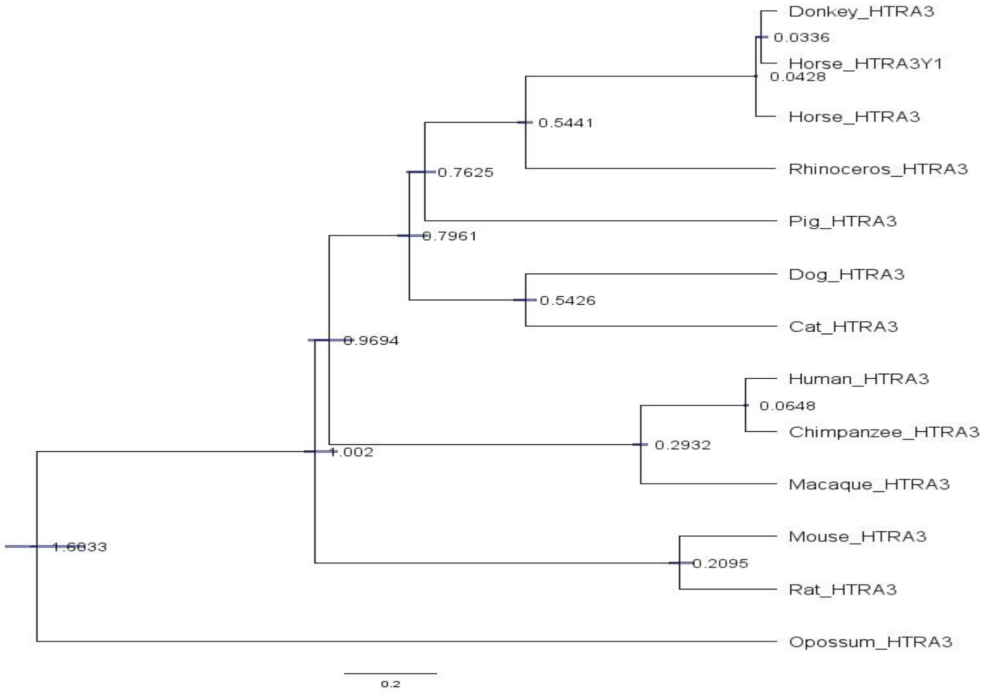

Supplementary Figure 9 continued

KDM5C

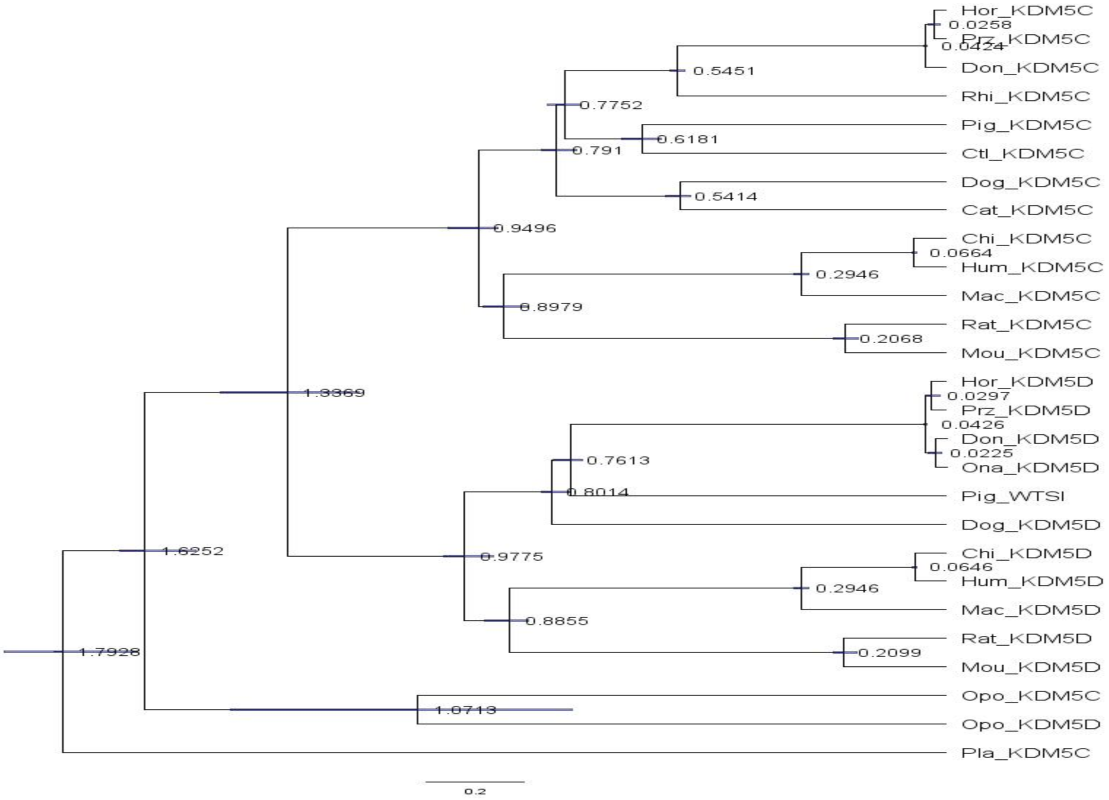

MYL9Y

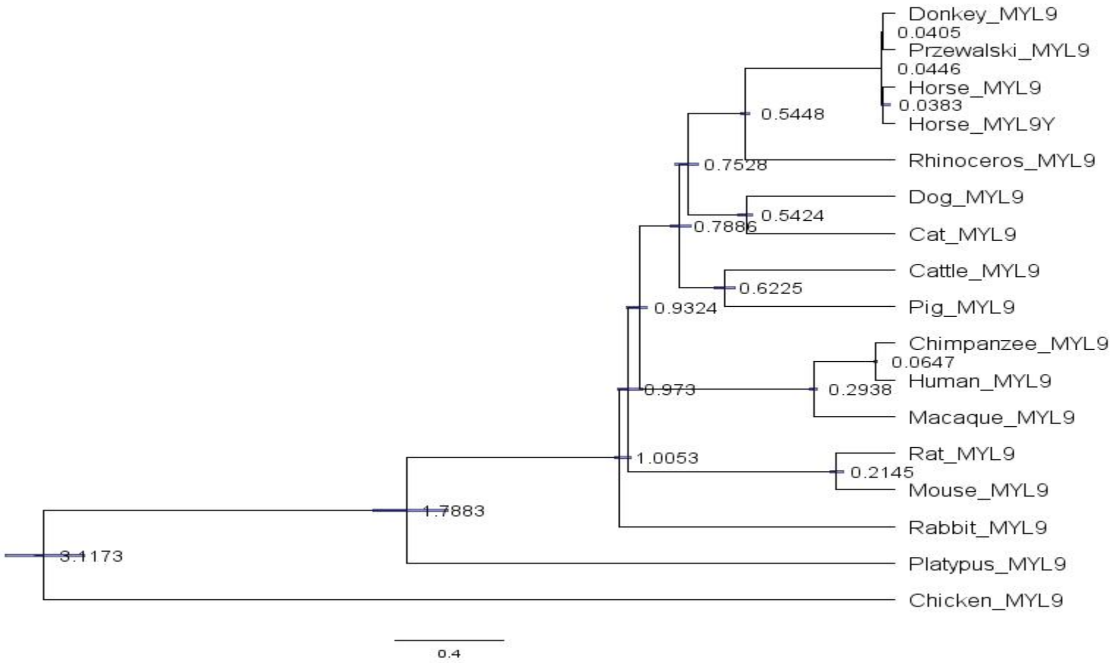

Supplementary Figure 9 continued

NLGN4Y

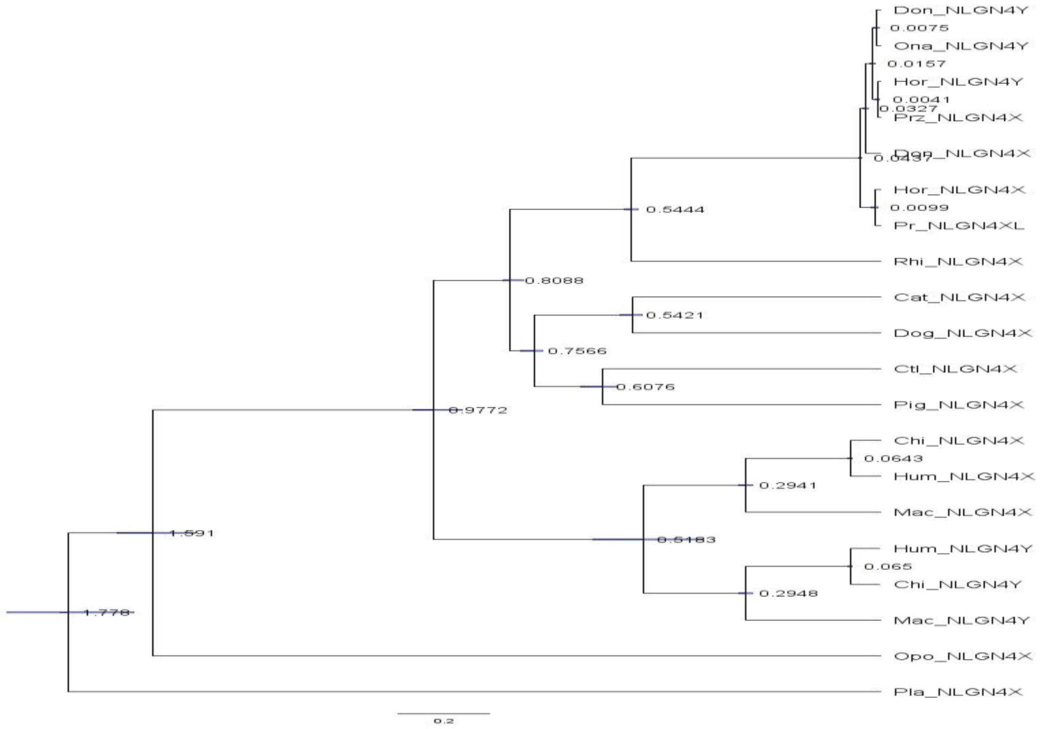

OFD1Y

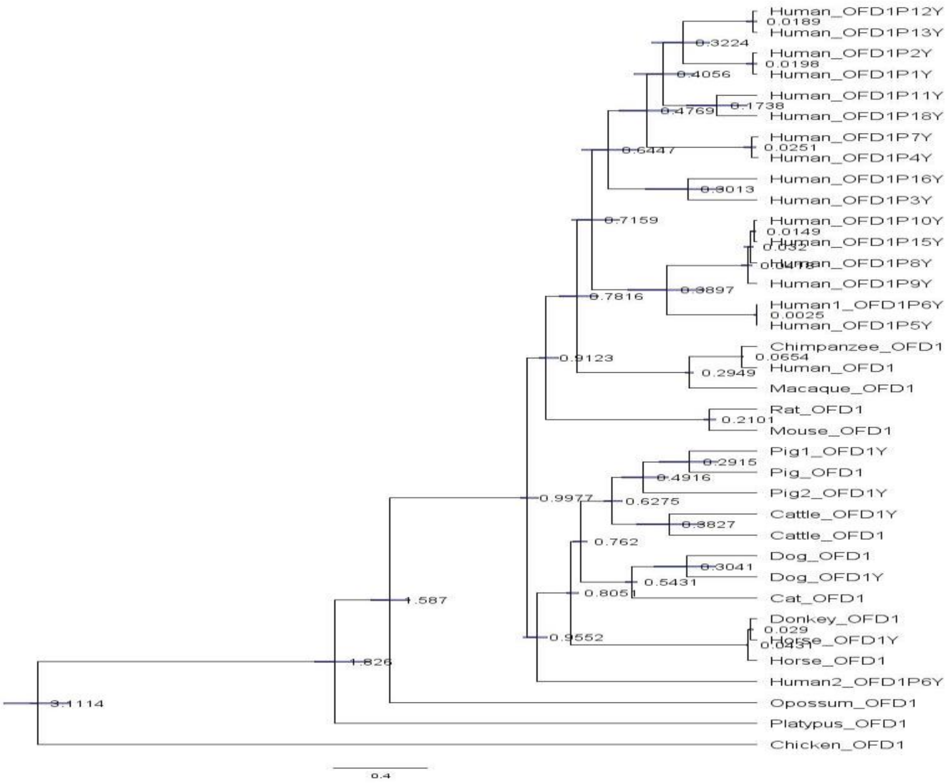

Supplementary Figure 9 continued

OR8J1Y

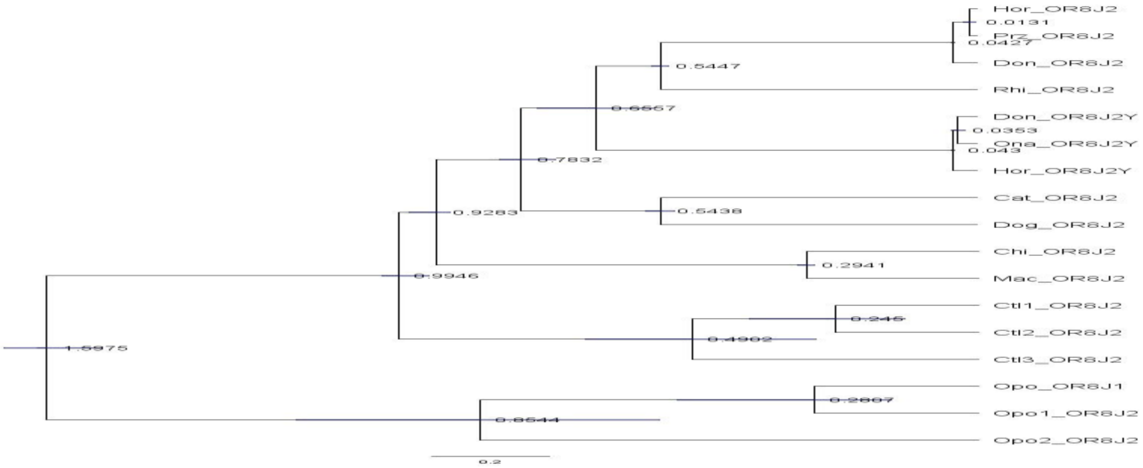

OR8K3Y

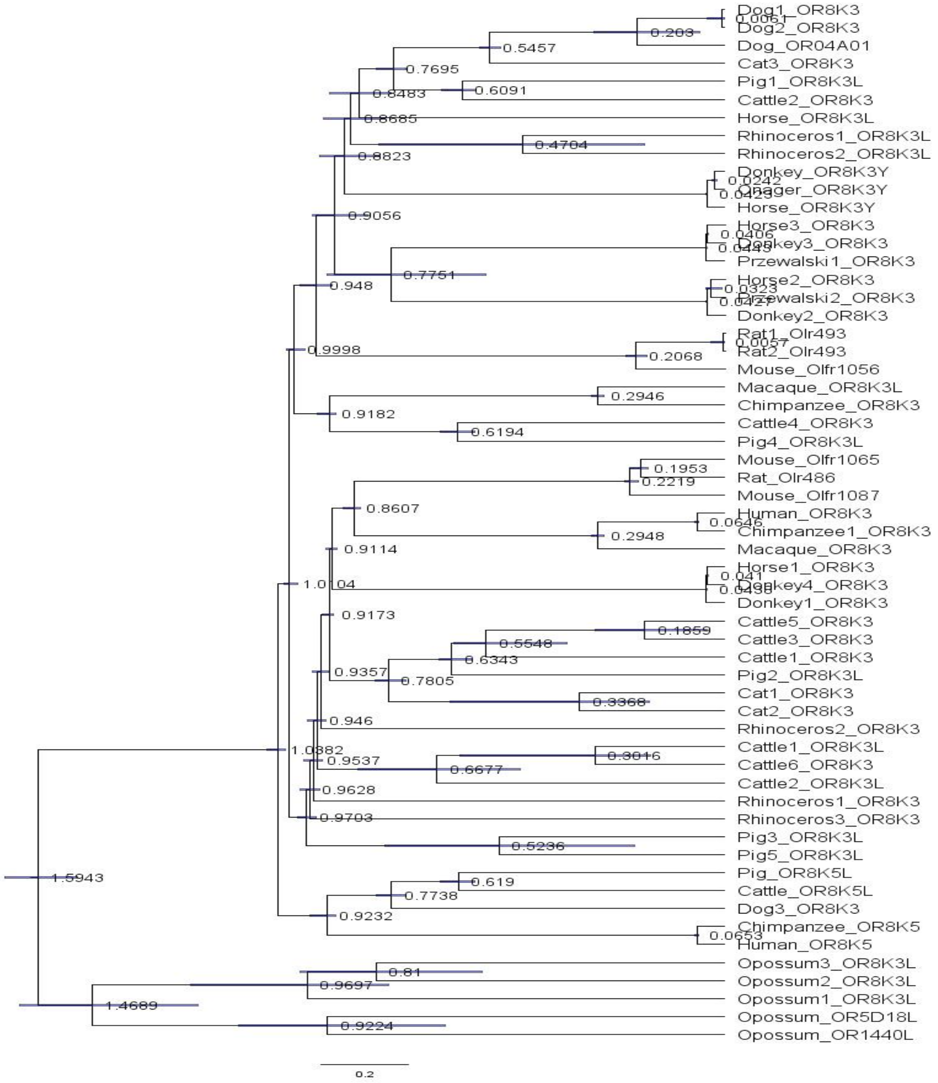

Supplementary Figure 9 continued

RPS3AY

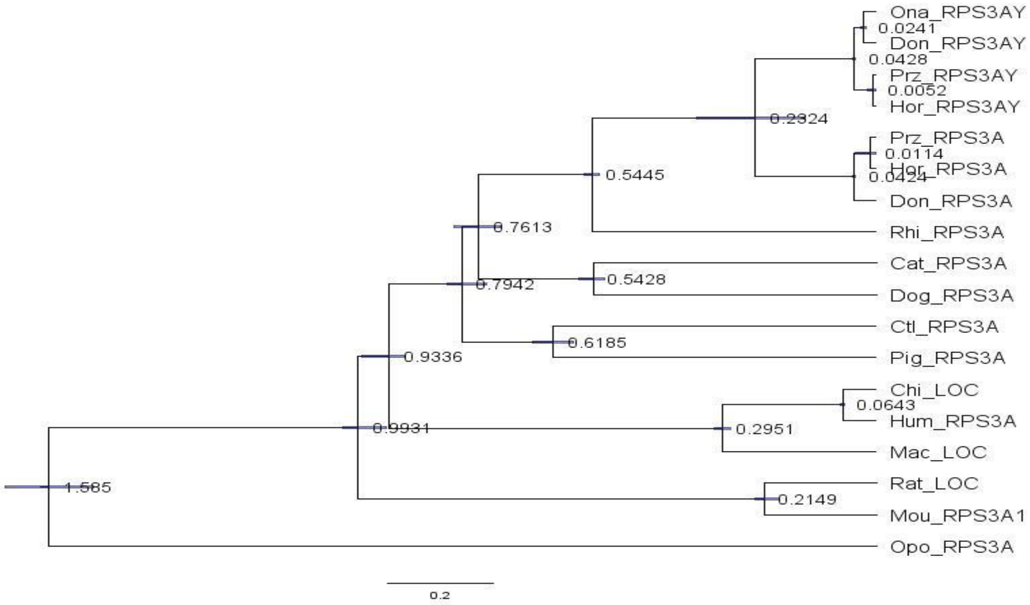

SH3TC1Y

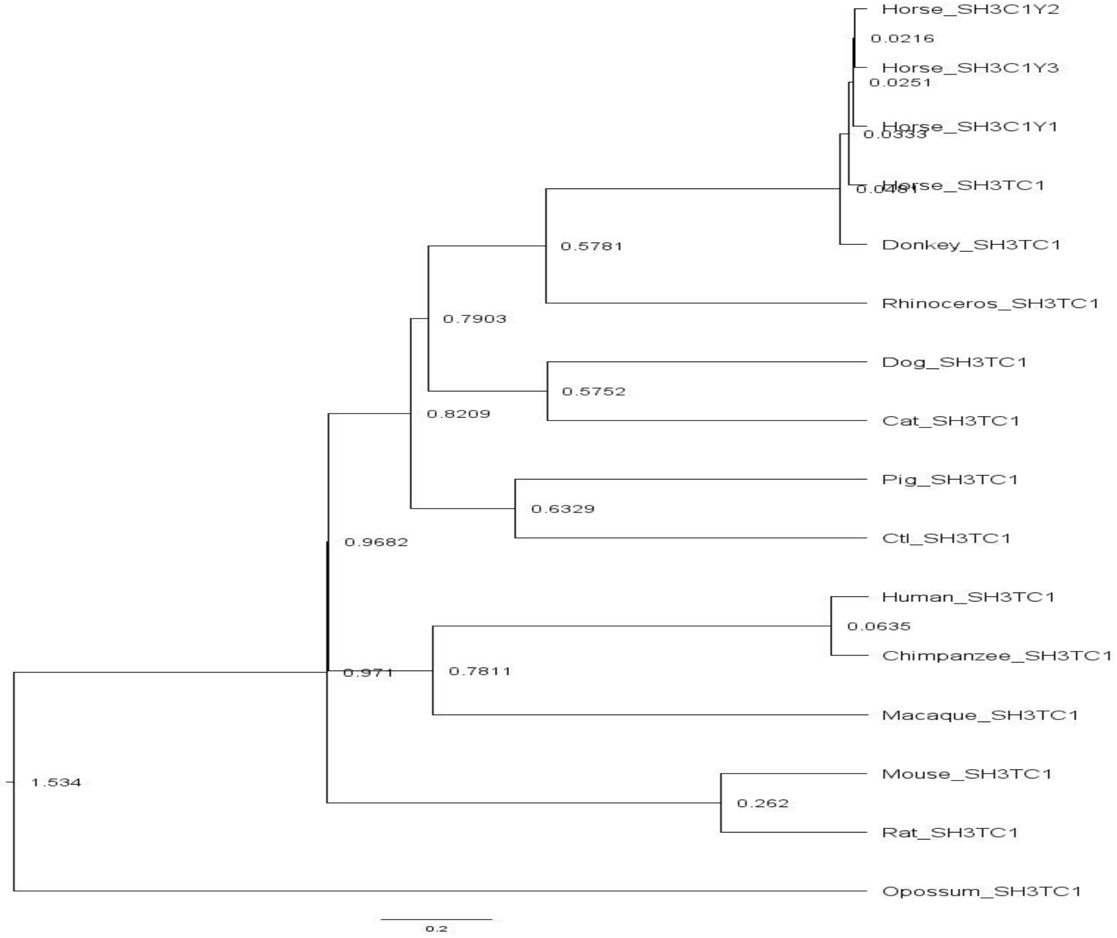

Supplementary Figure 9 continued

SHROOM2Y

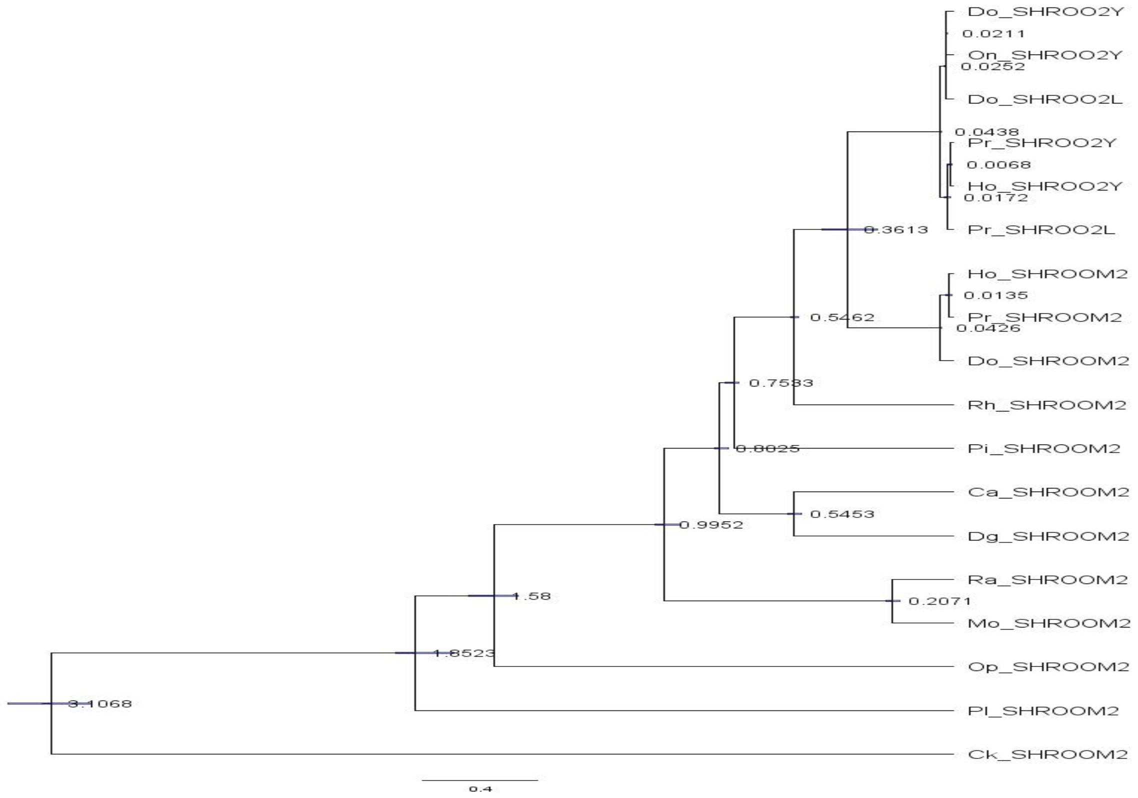

STSY

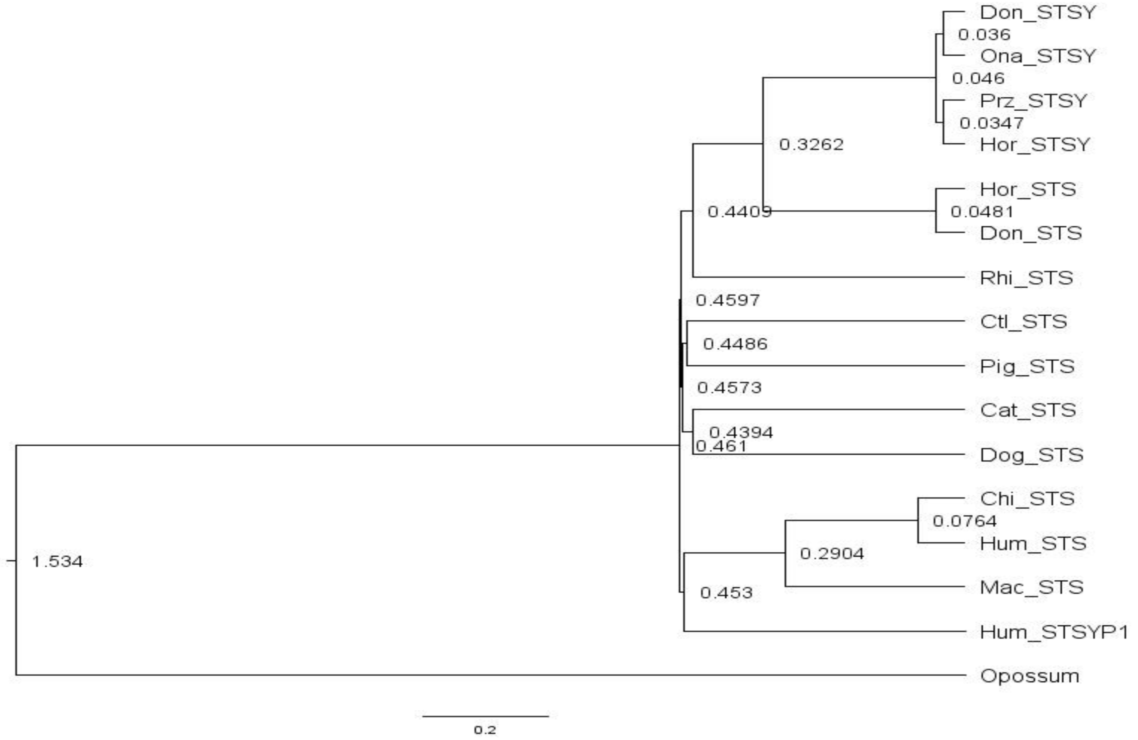

Supplementary Figure 9 continued

SYPY

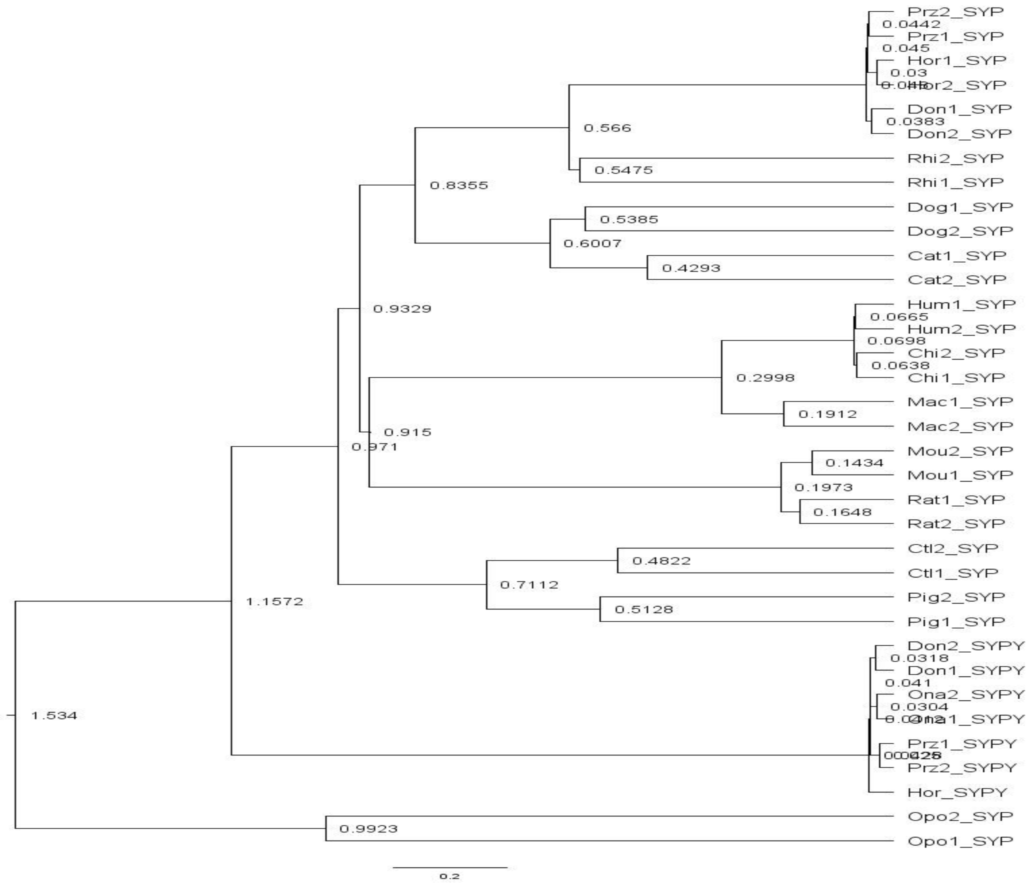

TAB3Y

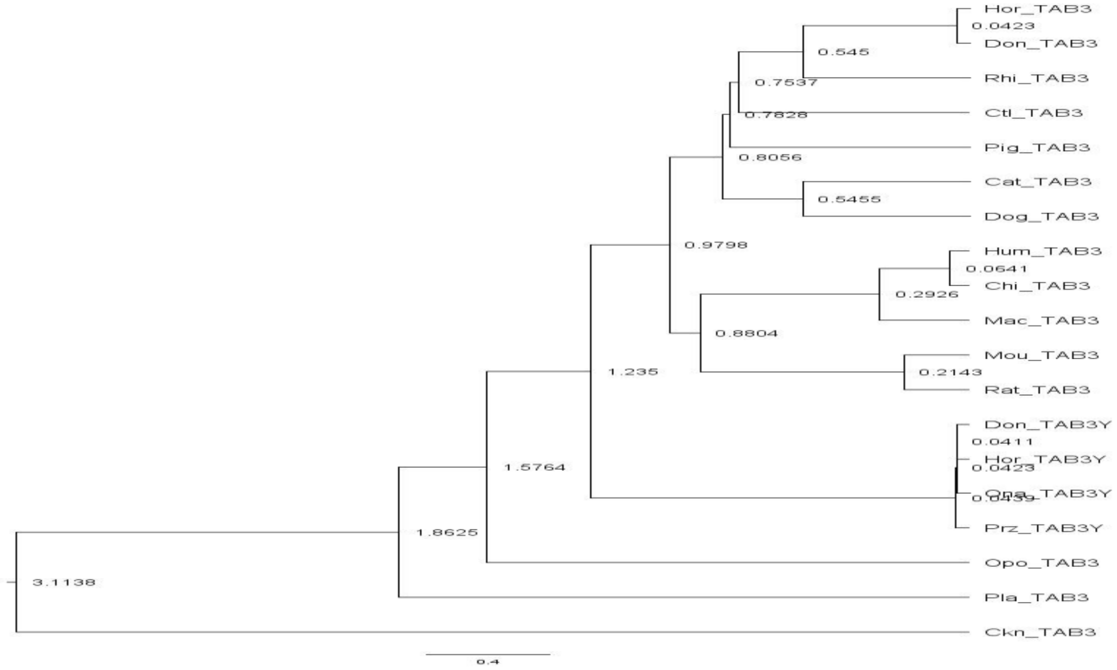

Supplementary Figure 9 continued

TBL1Y

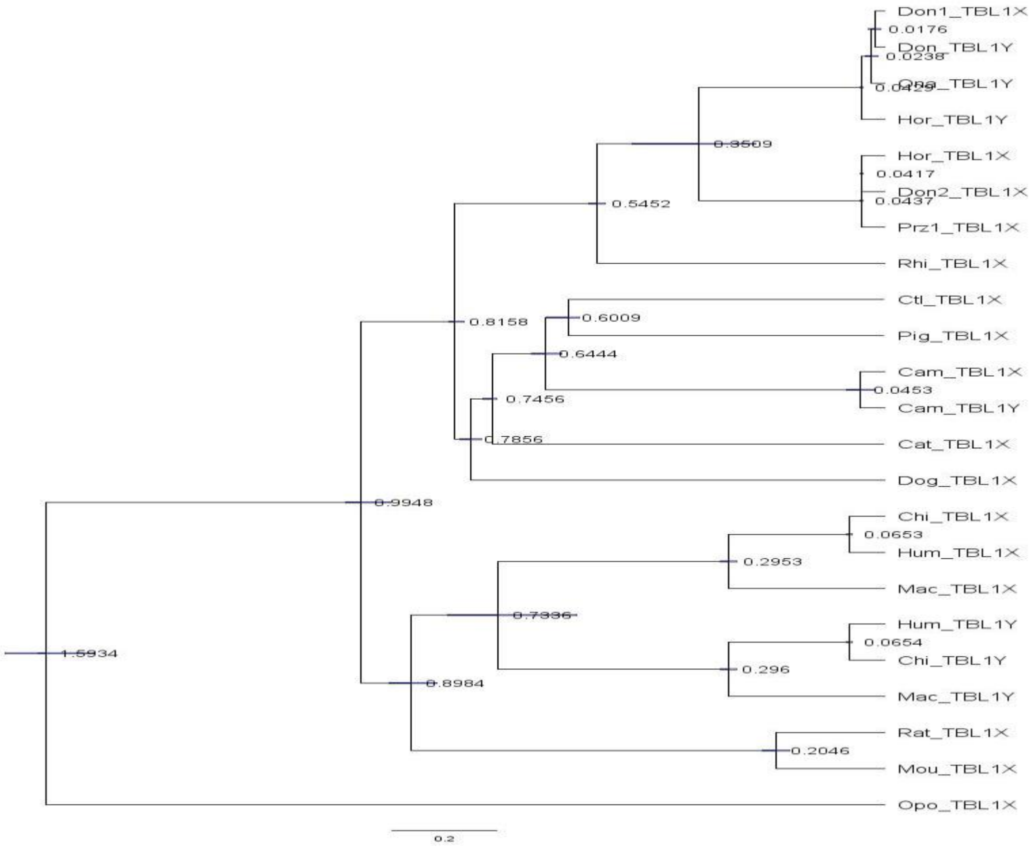

TIGD1Y

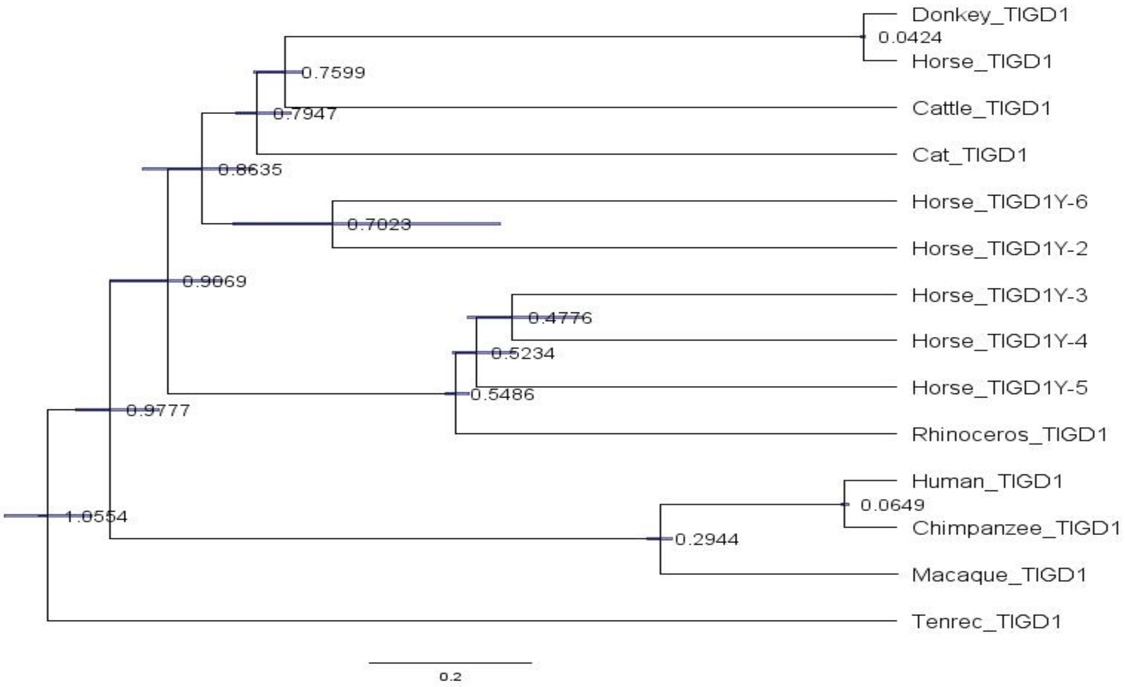

Supplementary Figure 9 continued

TMSB4Y

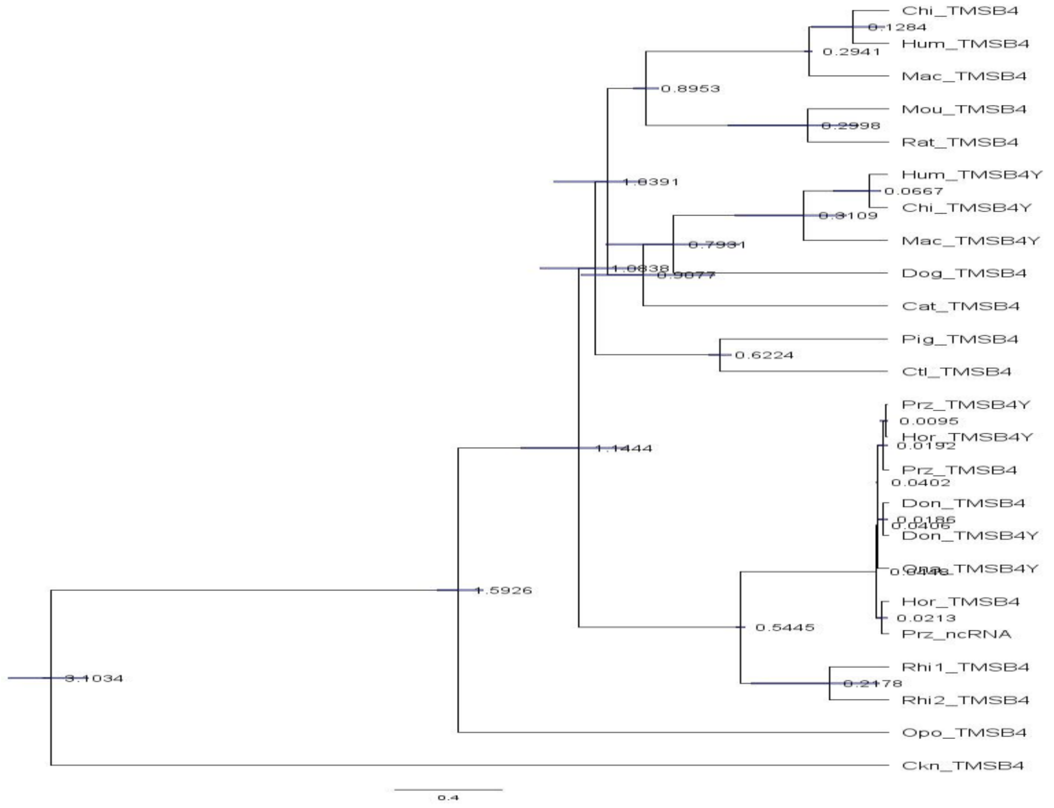

TXLNGY

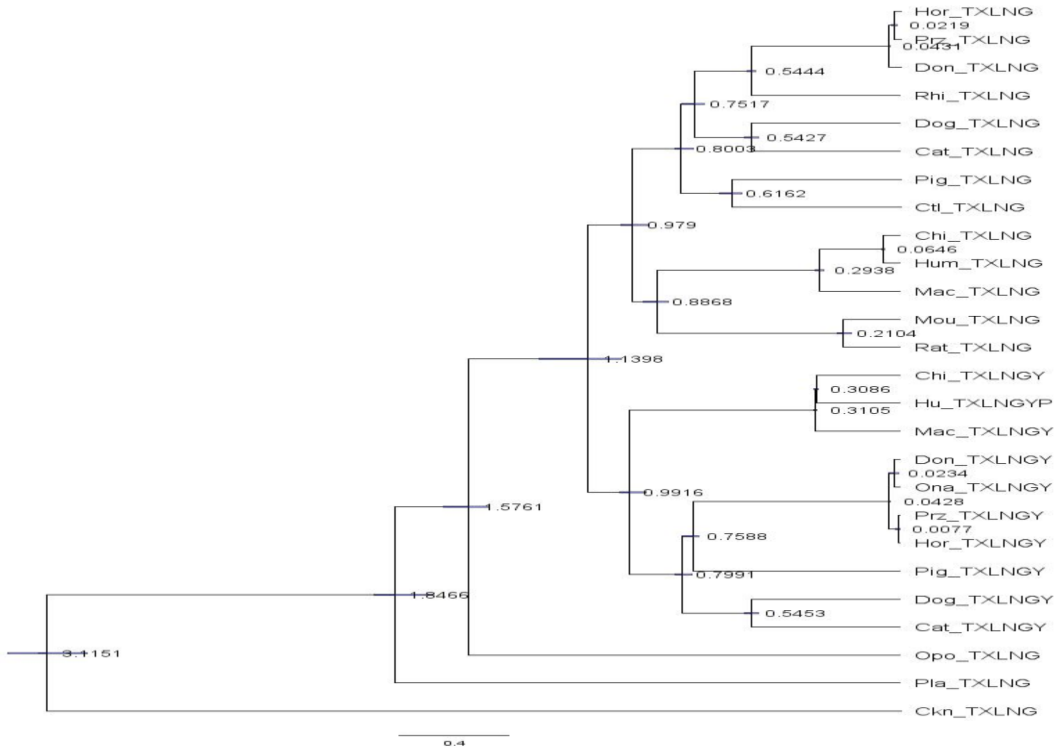

Supplementary Figure 9 continued

UBA1Y

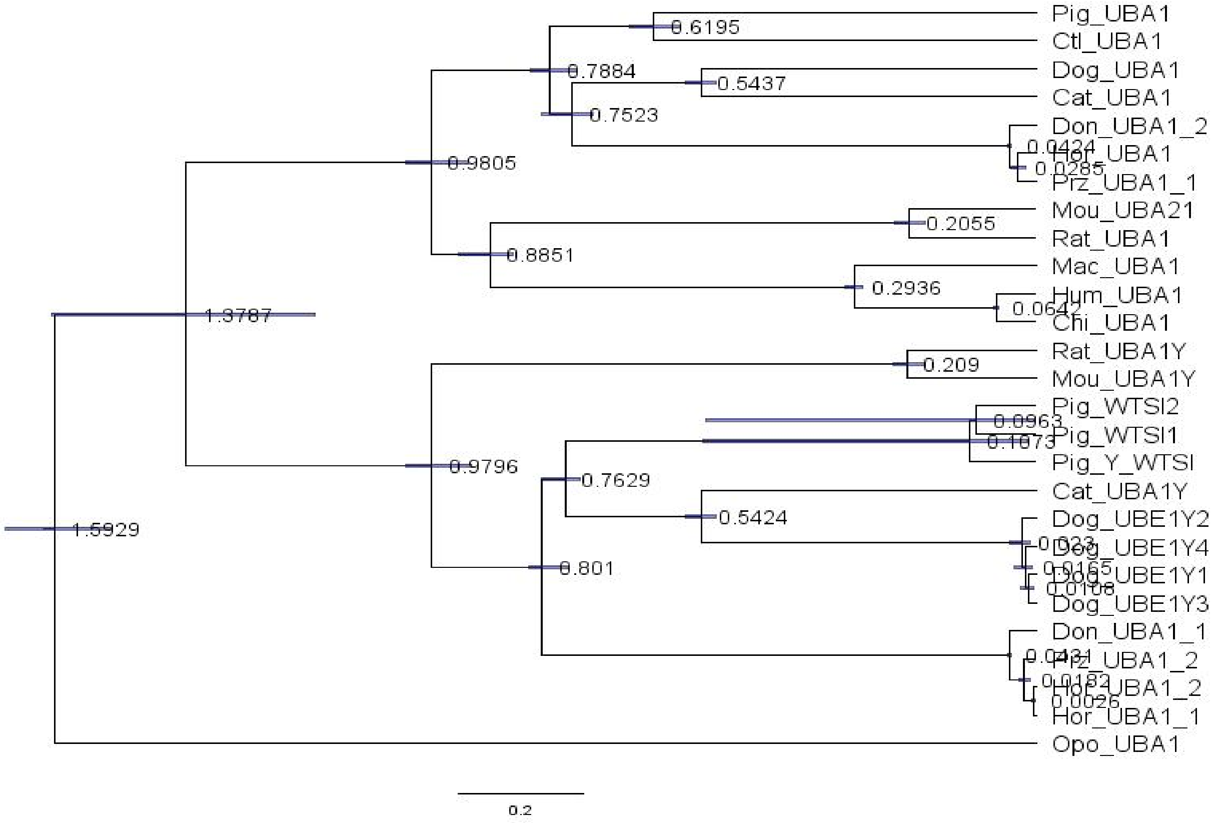

USP9Y

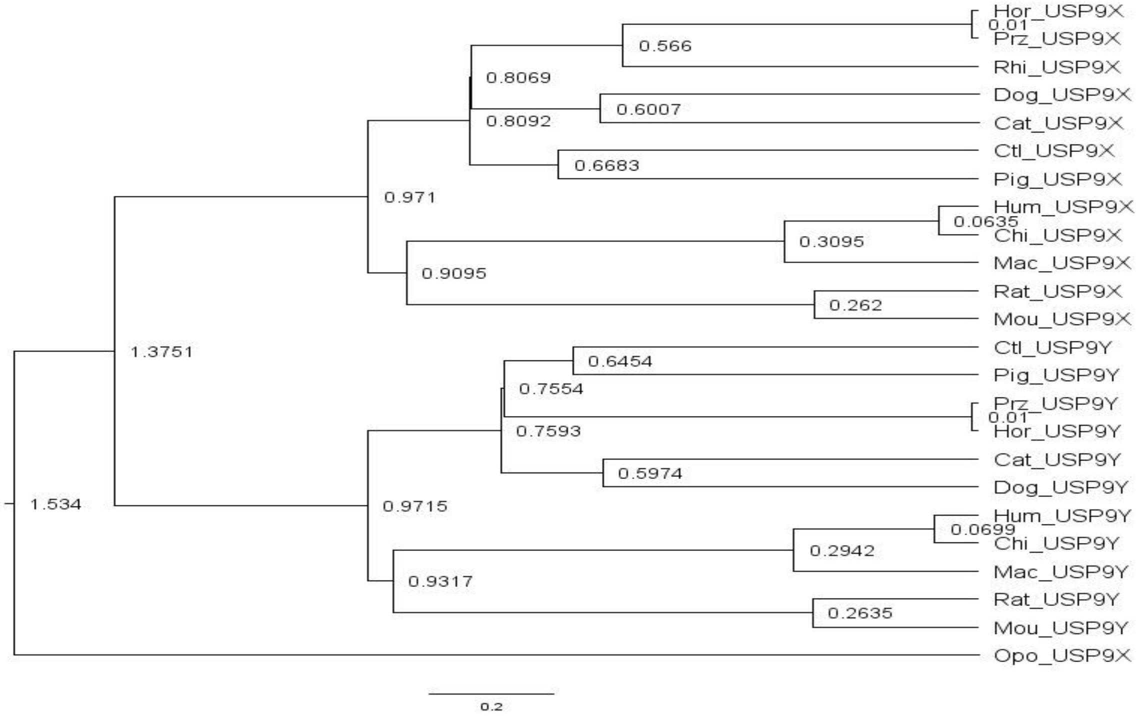

Supplementary Figure 9 continued

UTY

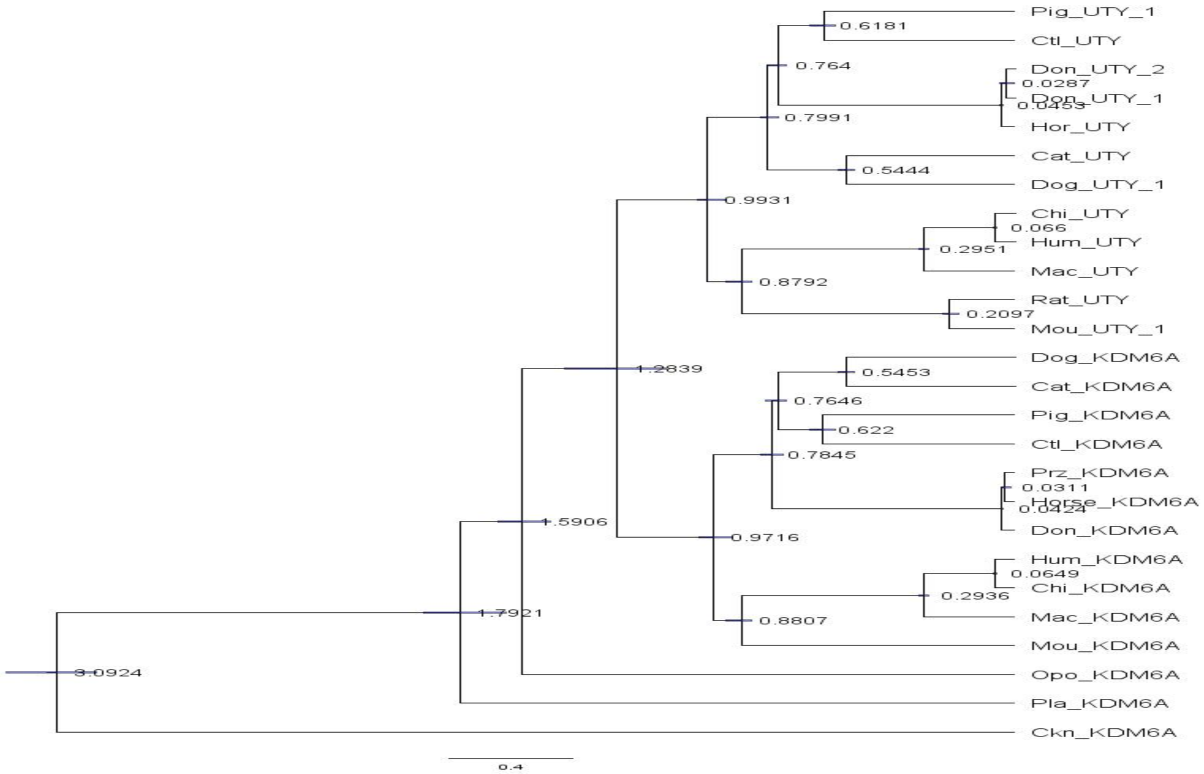

WWC3Y

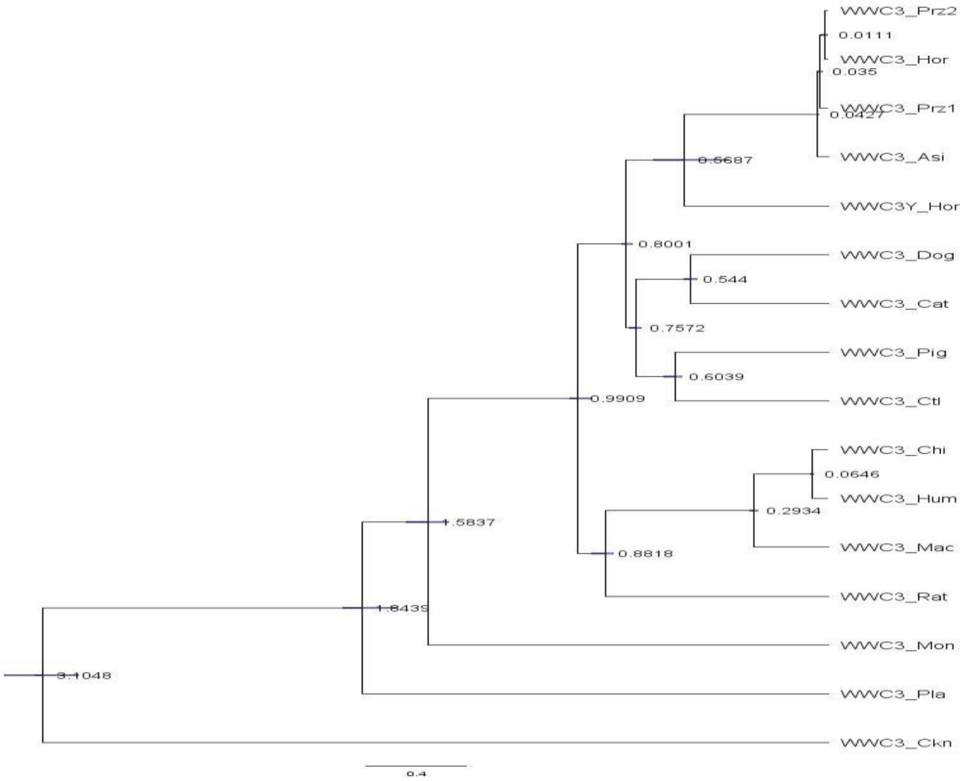

Supplementary Figure 9 continued

XKR3Y

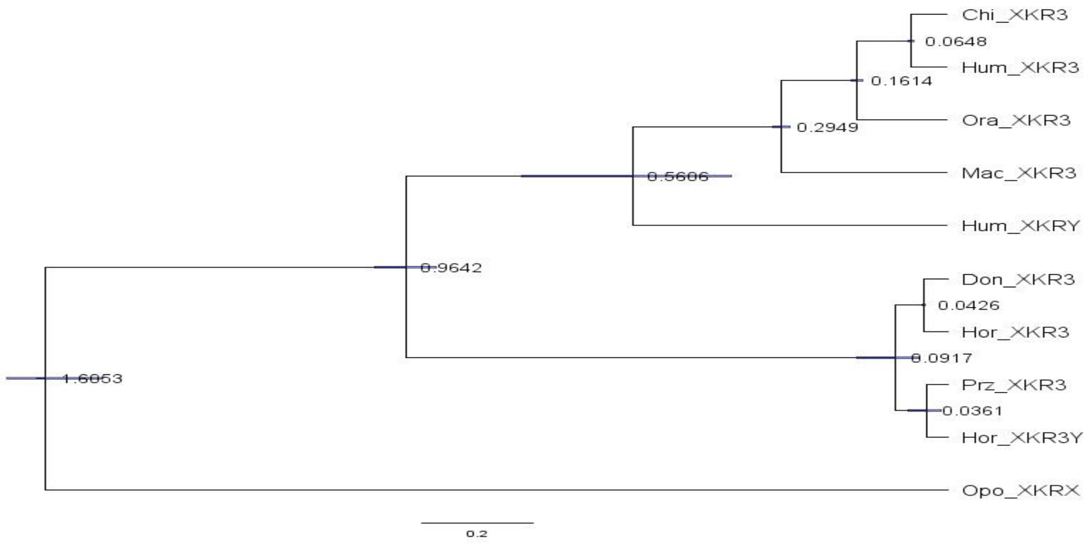

ZFY

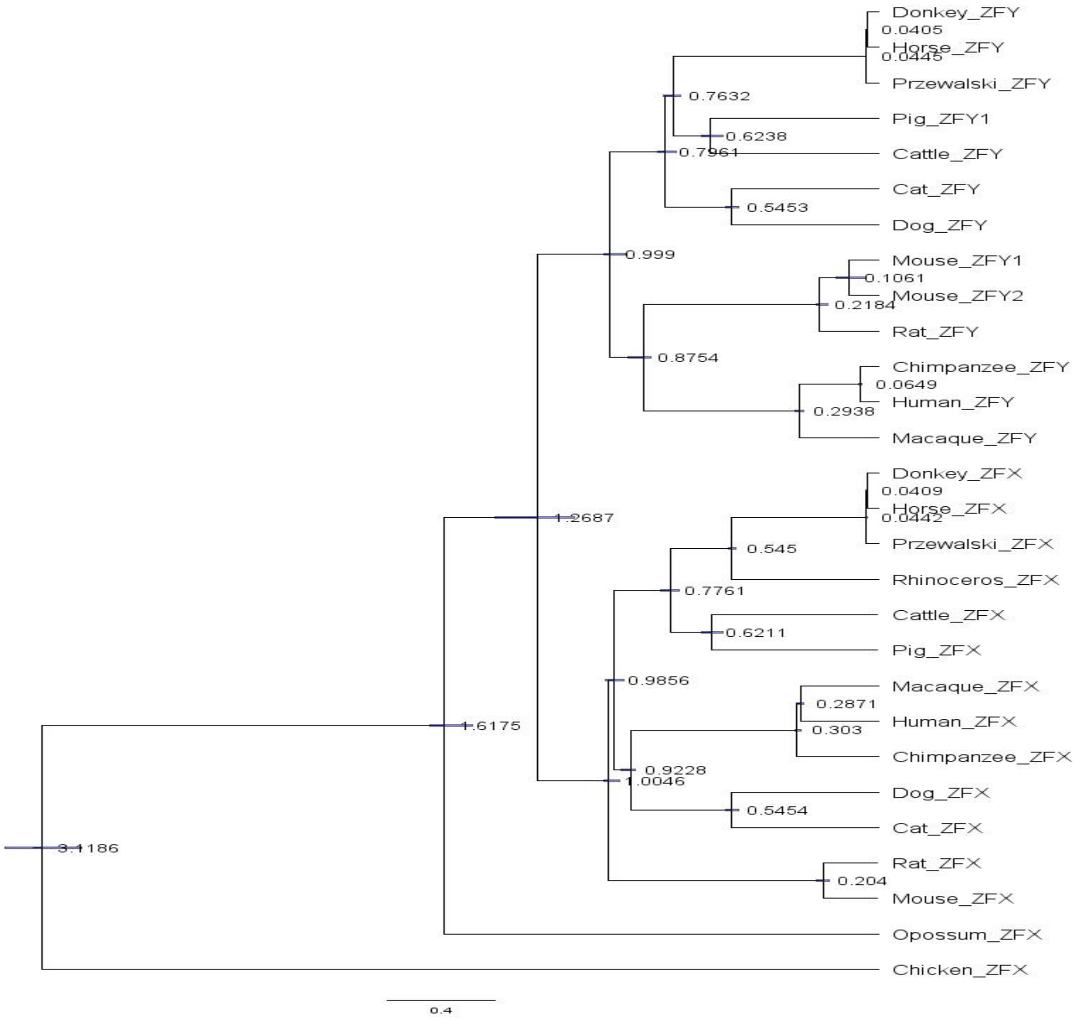

Supplementary Figure 9 continued

ZRSR2Y

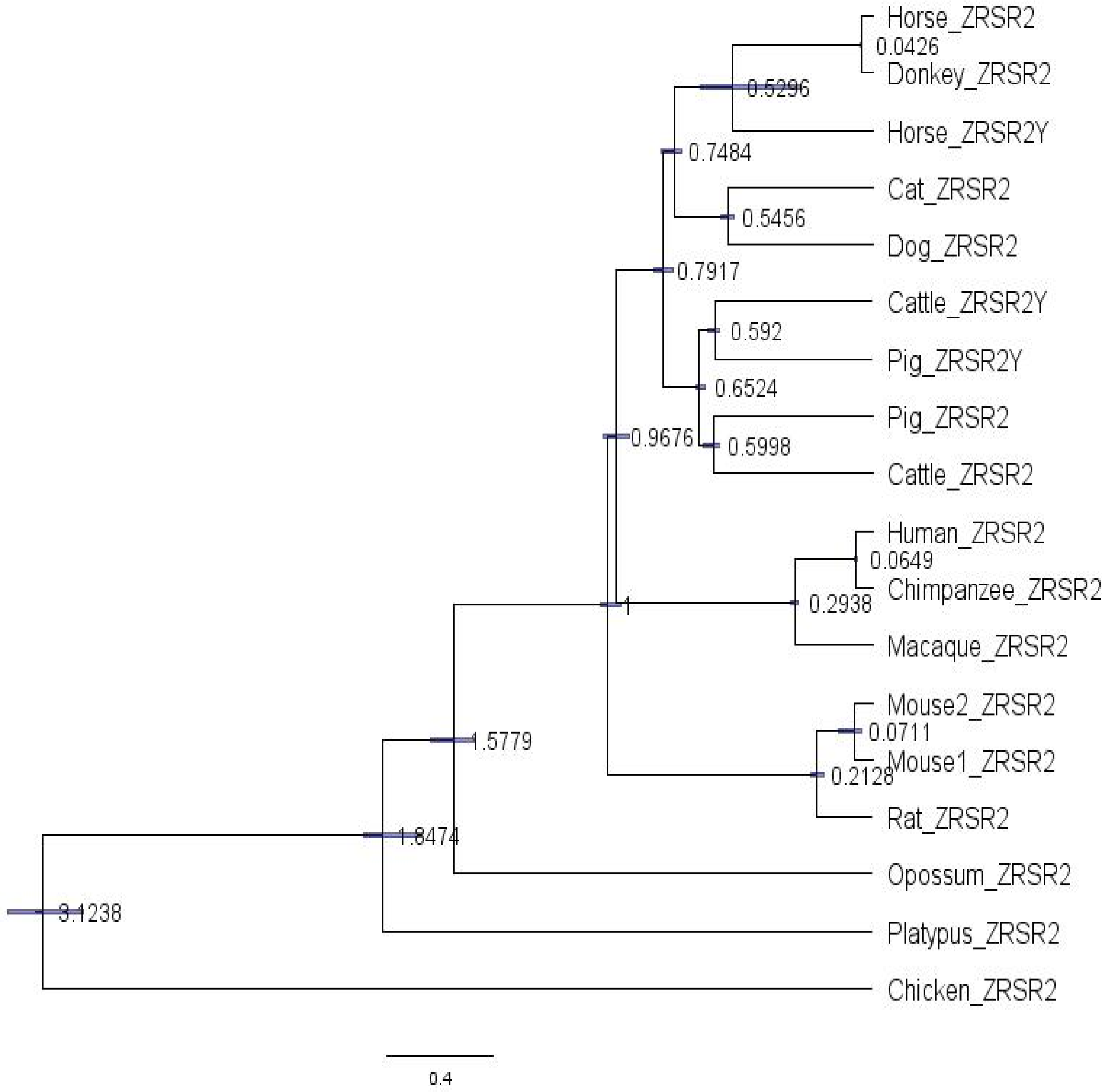

Supplementary Fig. 9. Time trees. Time divergence trees for 37 eMSY genes constructed with MCMCTREE (PAML v.4.9f) using soft fossil constraints (Supplementary Data 13). Information for individual genes is in Supplementary Data 7.

Supplementary Figure 10

AMELY

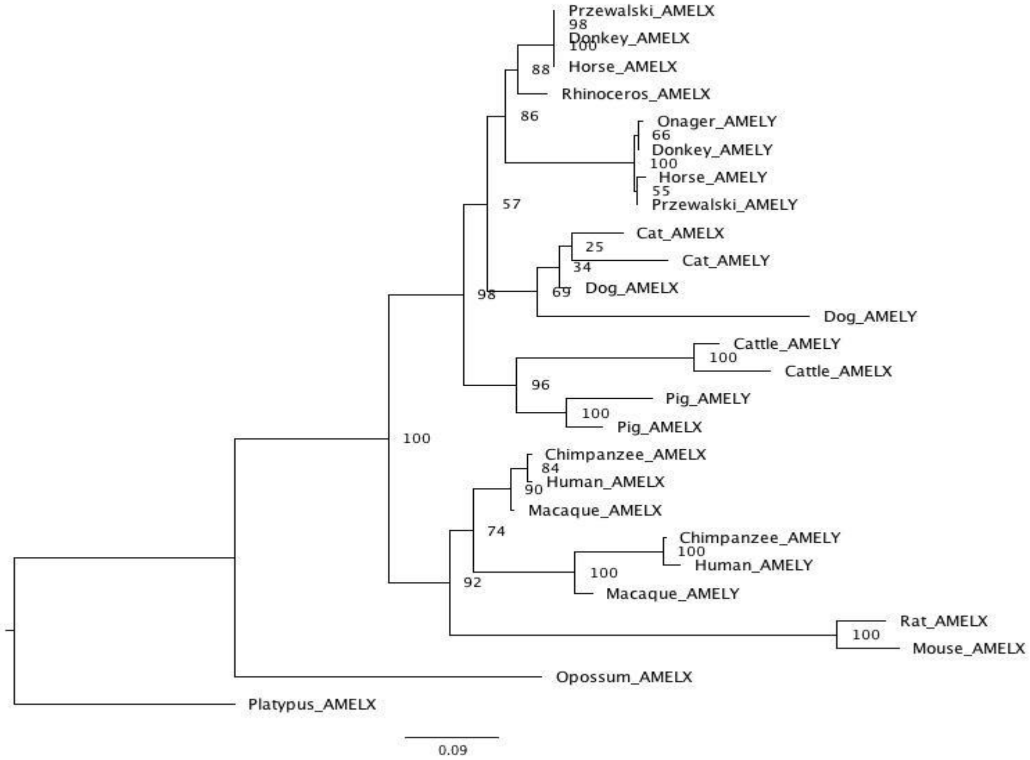

ANOS1Y

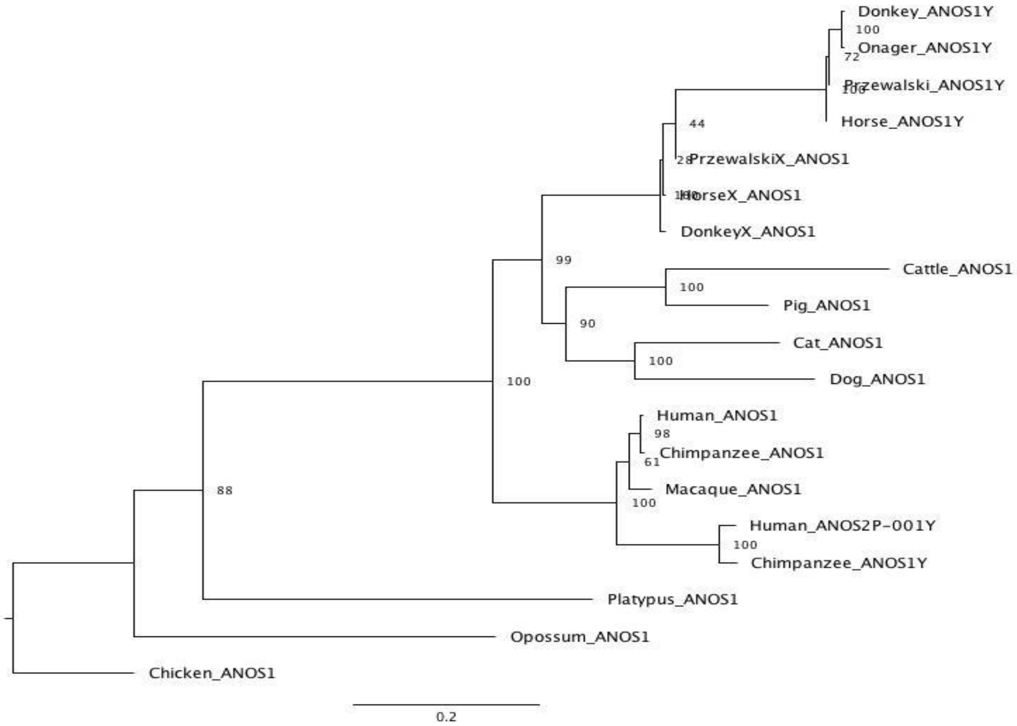

Supplementary Figure 10 continued
AP1S2Y

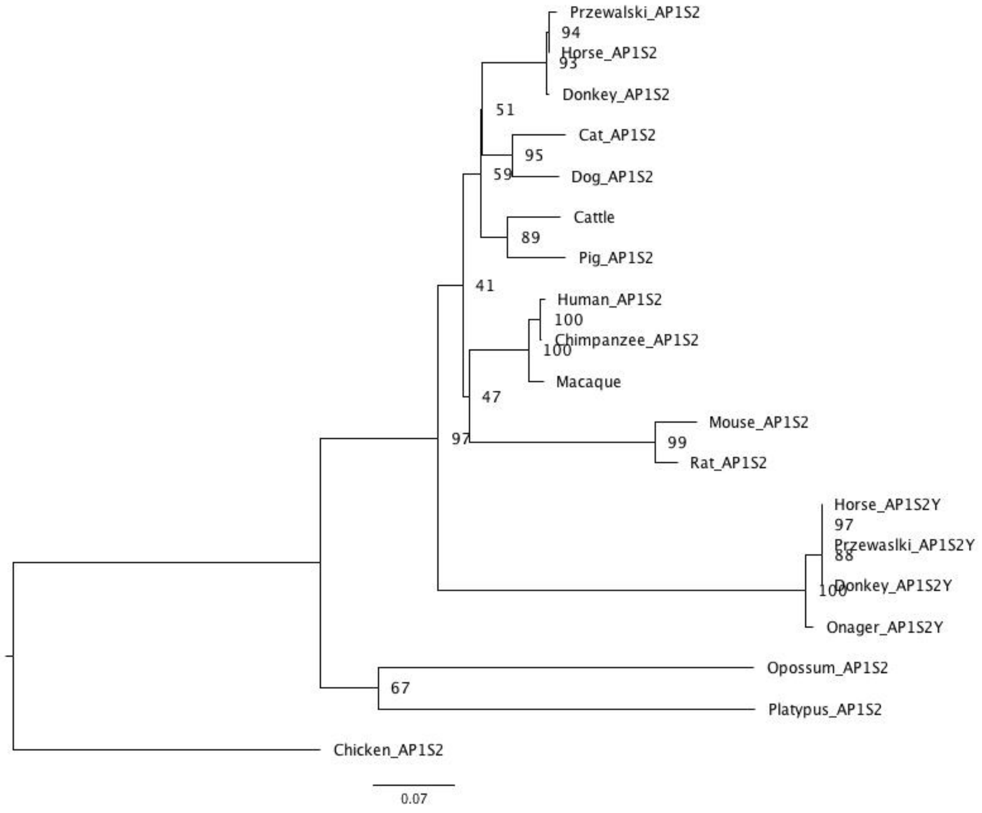

ARSFY

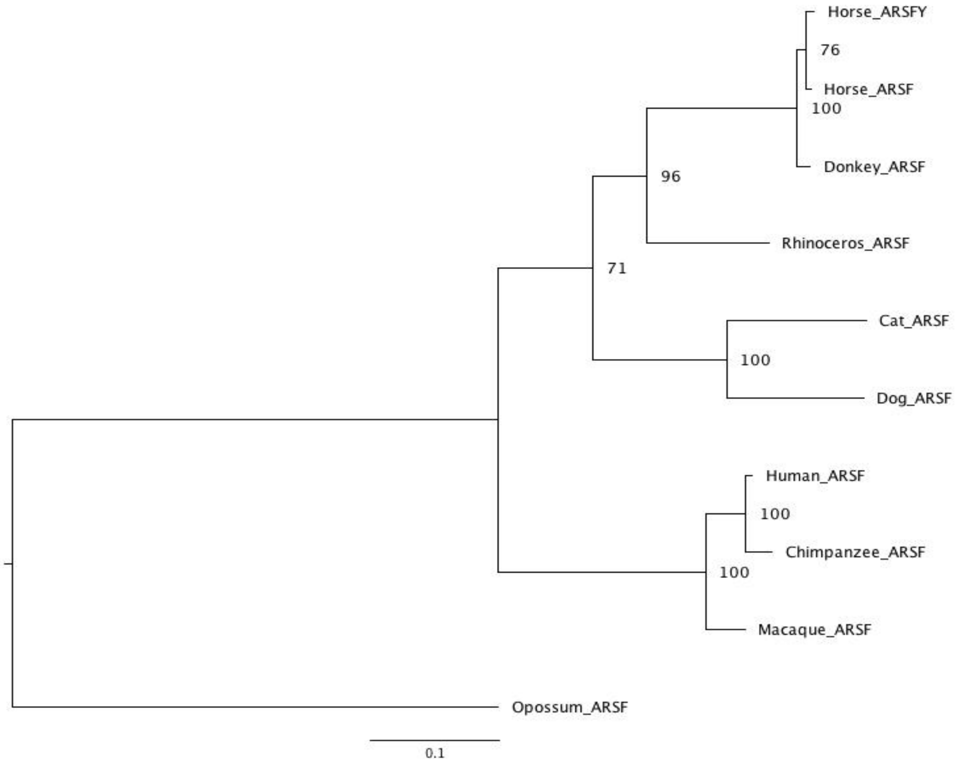

Supplementary Figure 10 continued
ARSHY

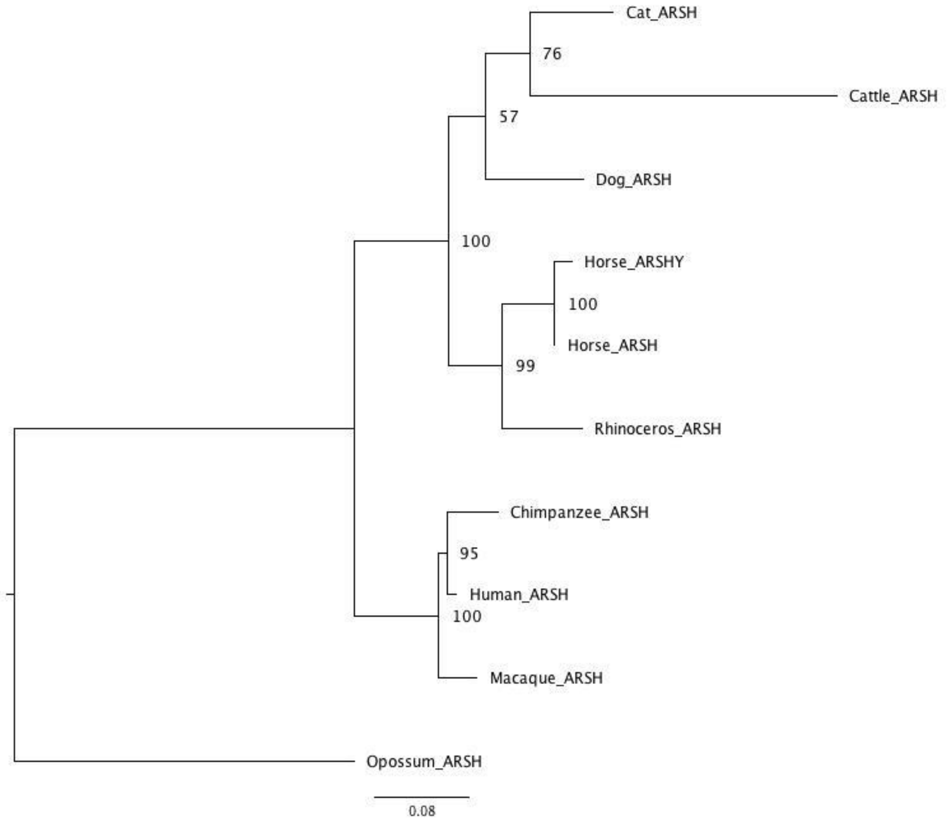

ATP6V0Y

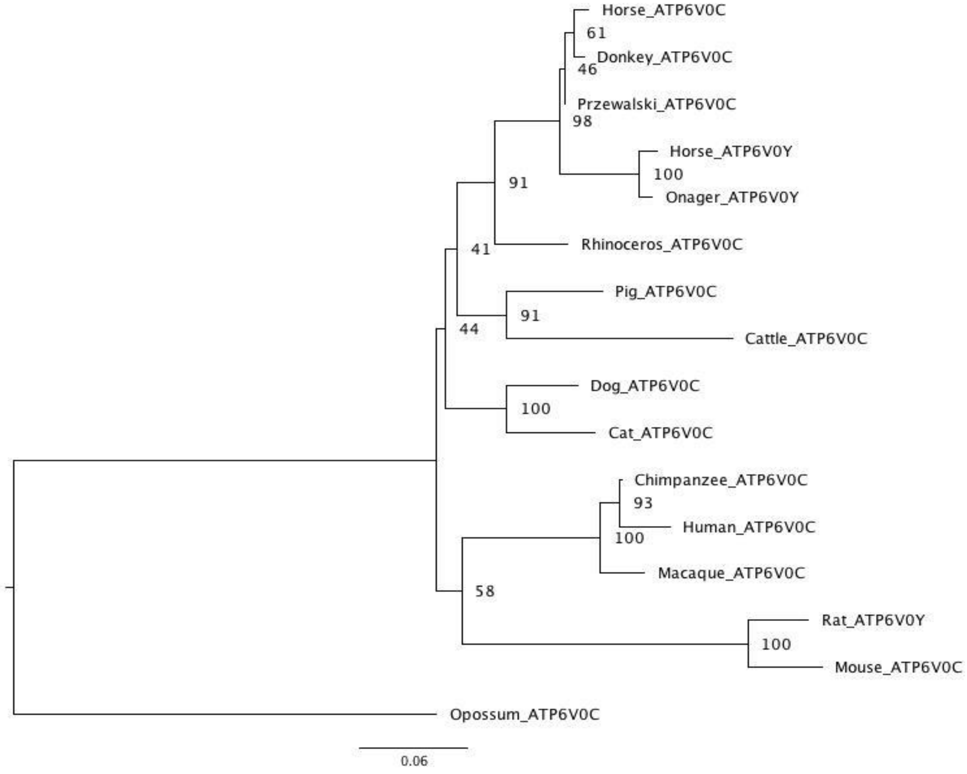

Supplementary Figure 10 continued
BCORY

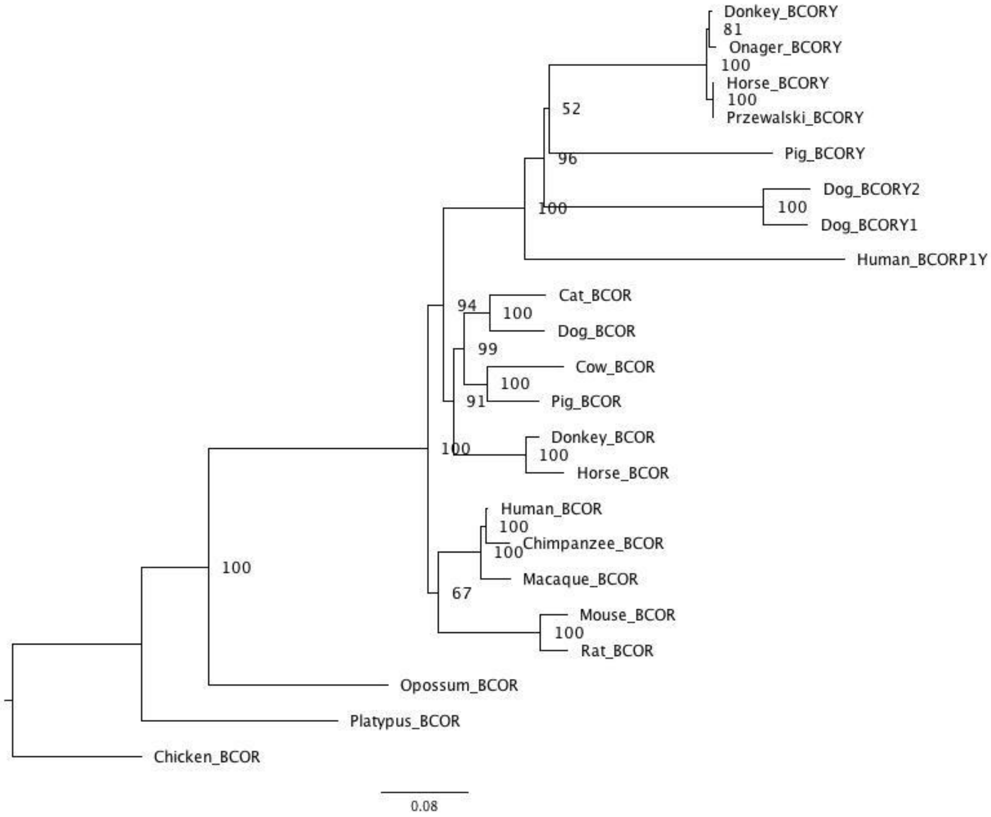

CUL4BY

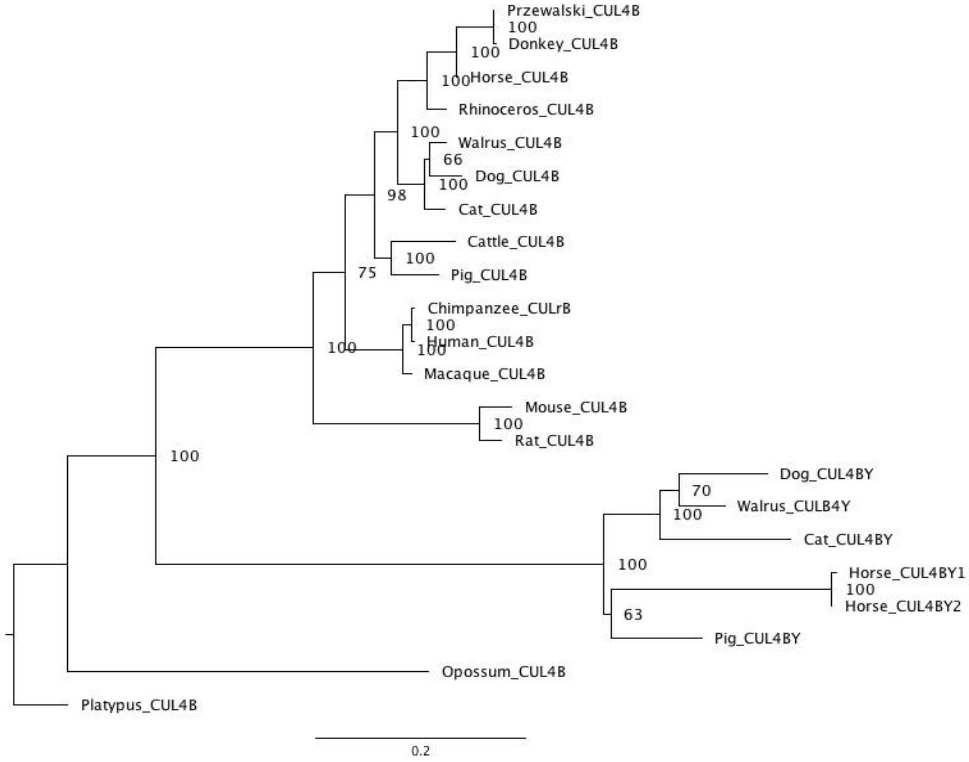

Supplementary Figure 10 continued
DDX3Y

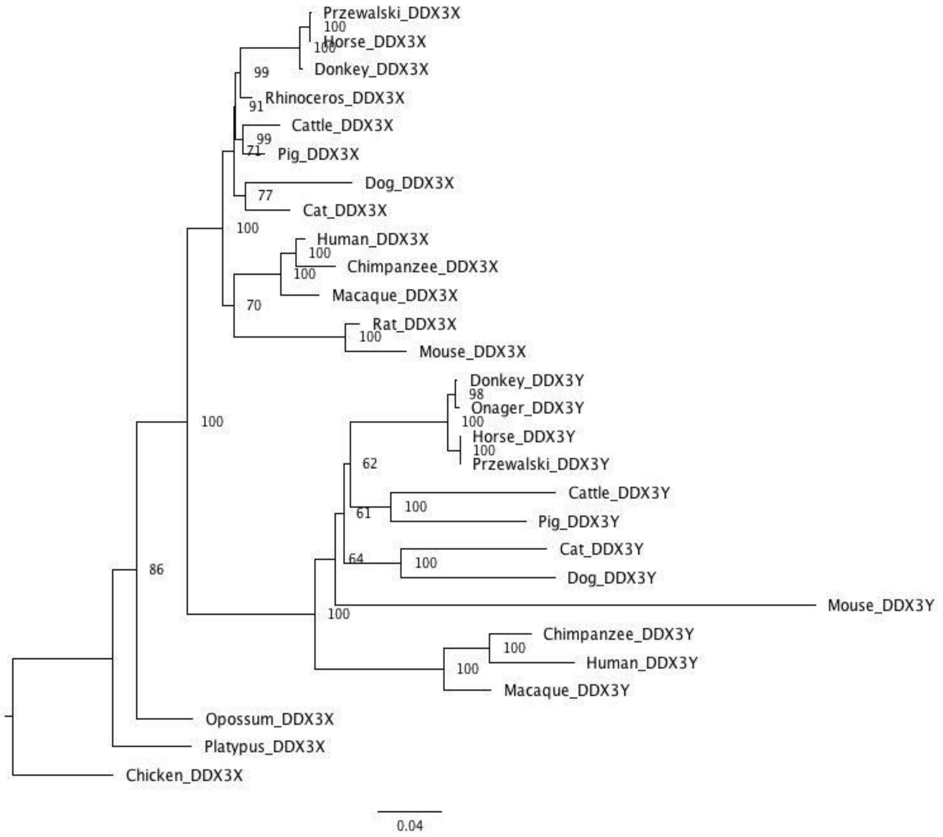

EIF1AY

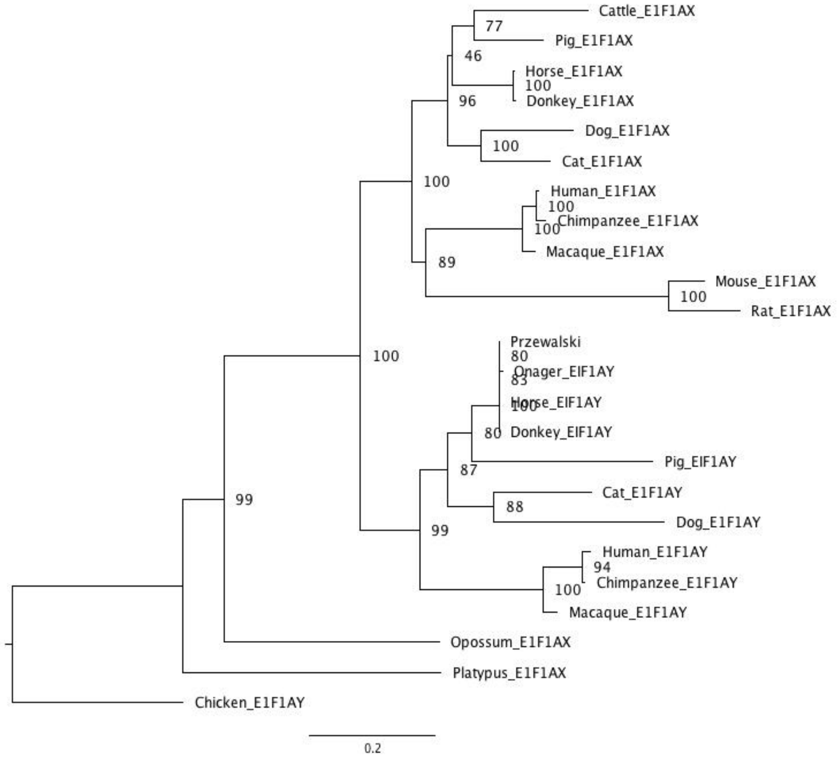

Supplementary Figure 10 continued
EIF2S3Y

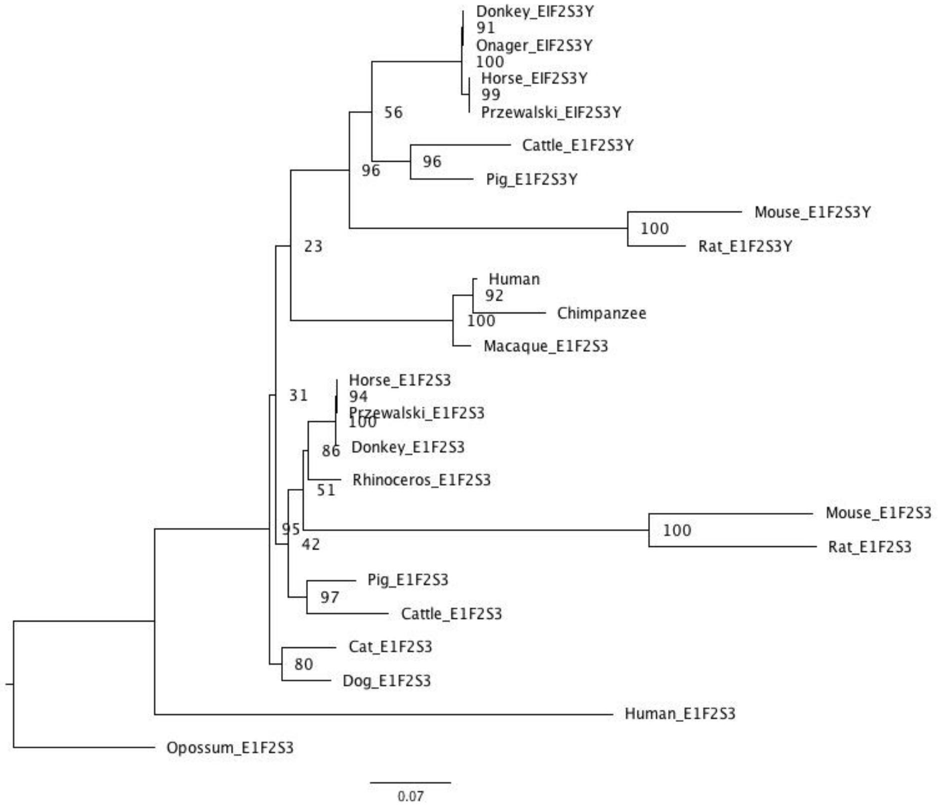

EIF3CY

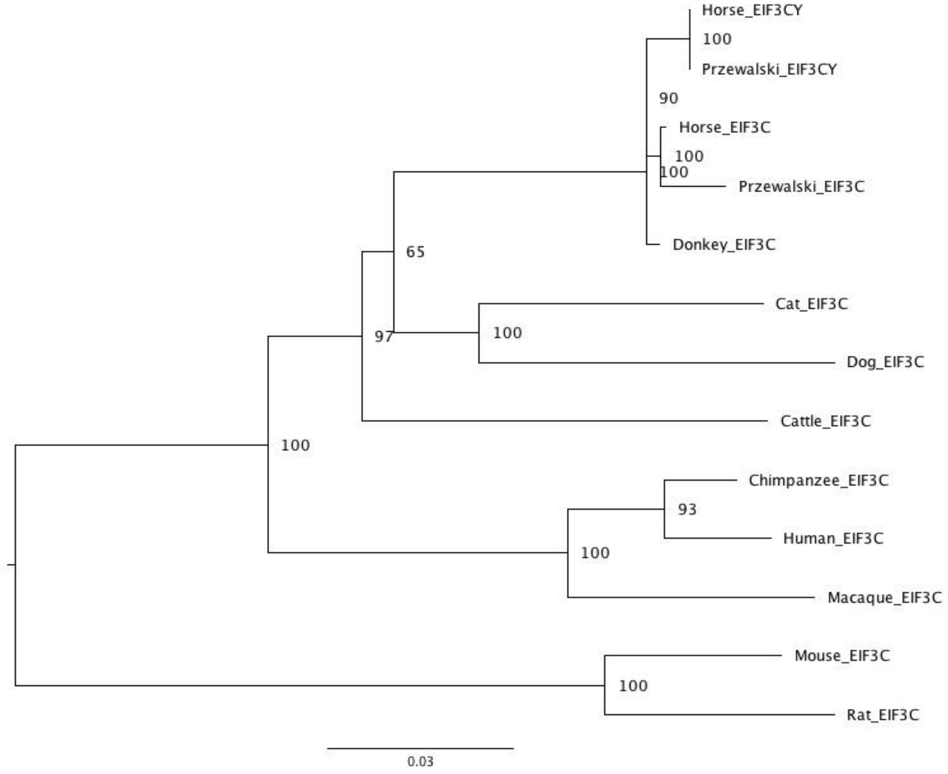

Supplementary Figure 10 continued
HSFY

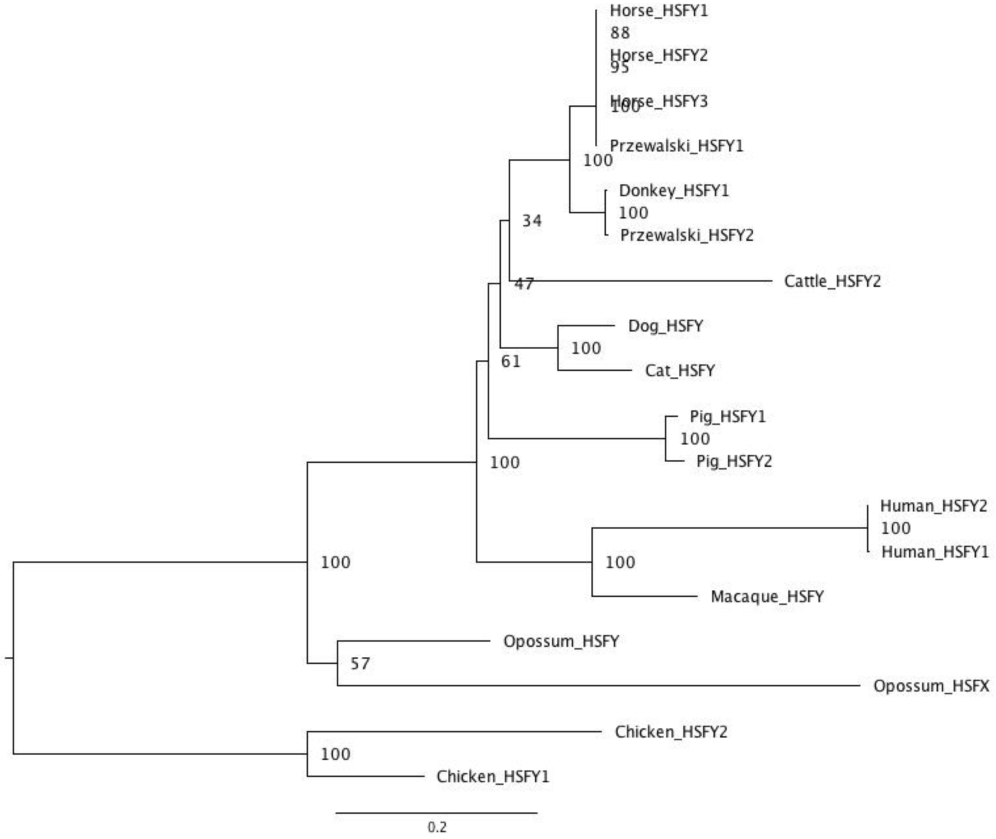

HSPA1LY

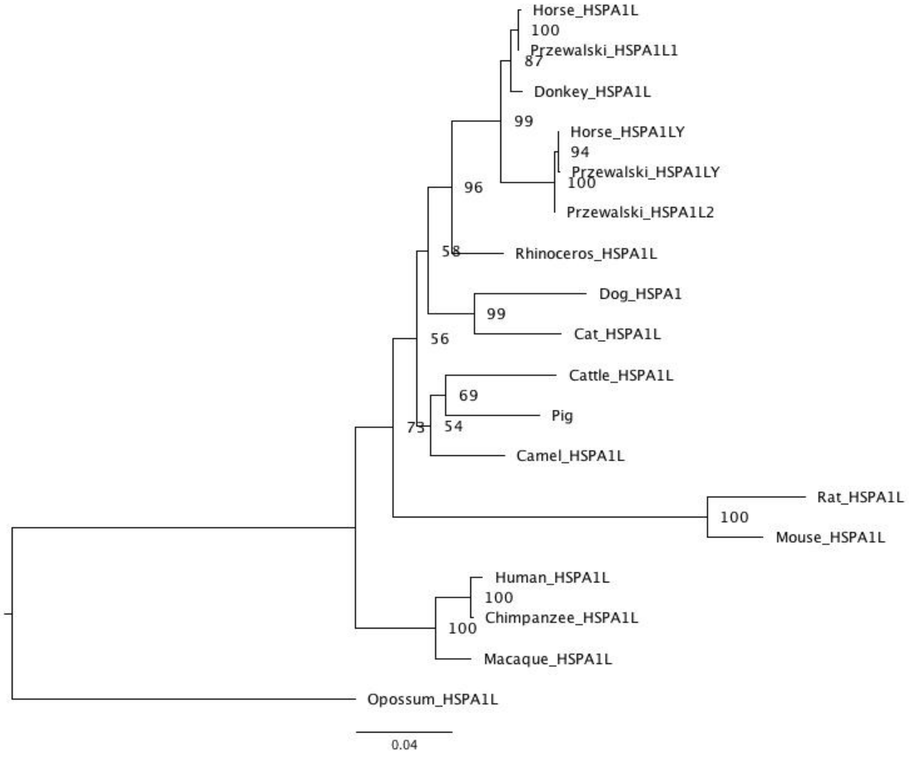

Supplementary Figure 10 continued

HTRA3Y

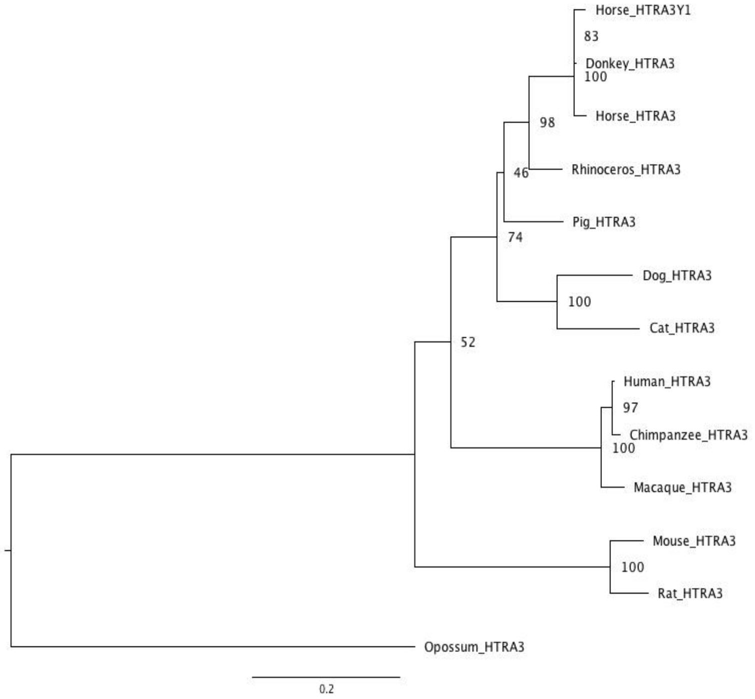

KDM5C

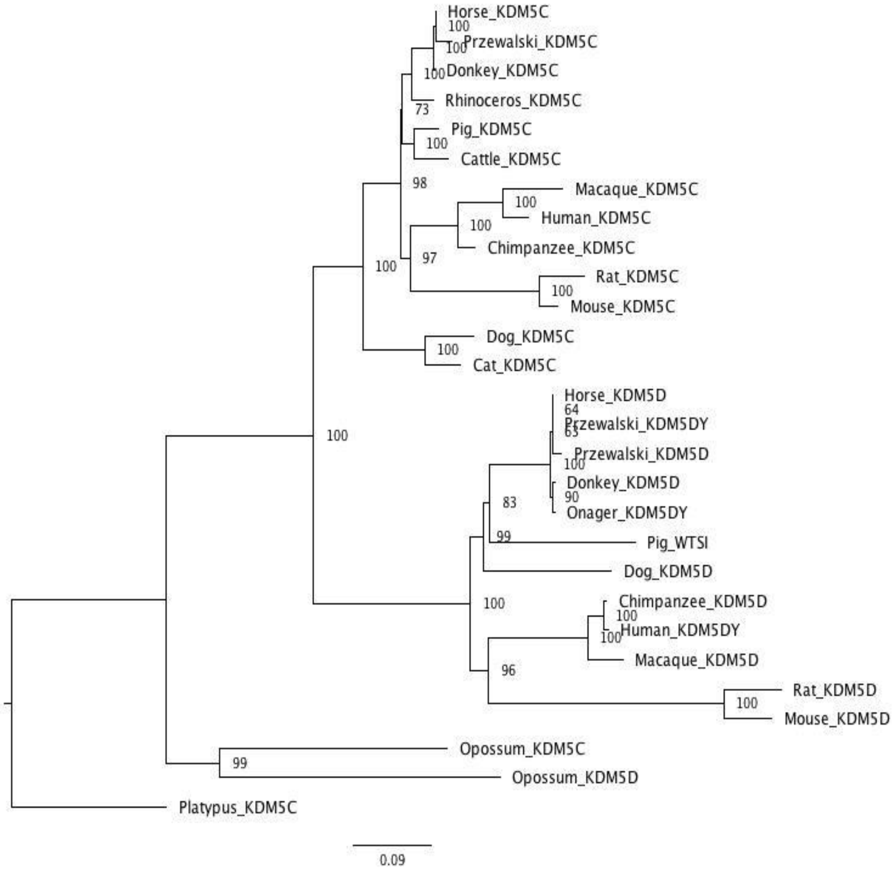

Supplementary Figure 10 continued
MYL9Y

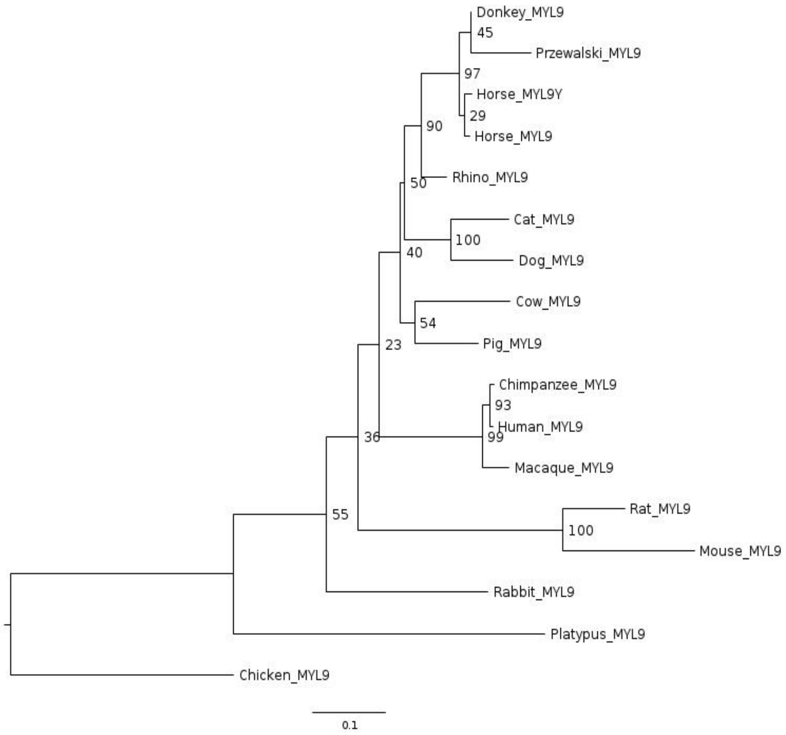

NLGN4Y

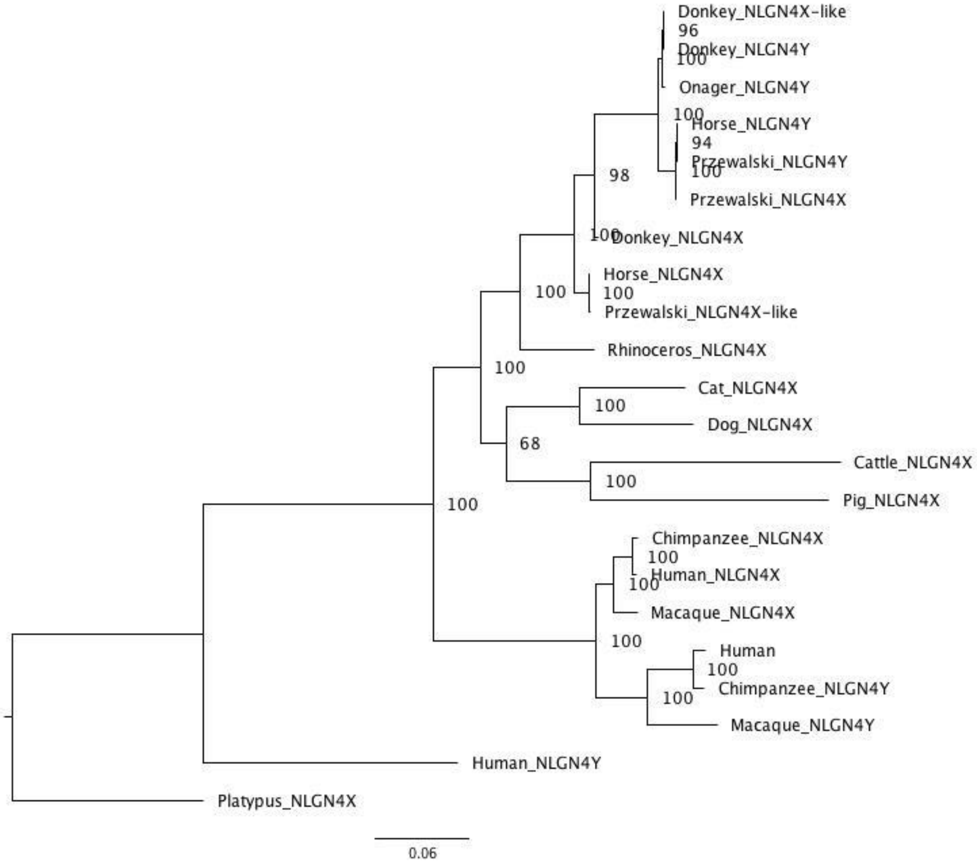

Supplementary Figure 10 continued

OFD1Y

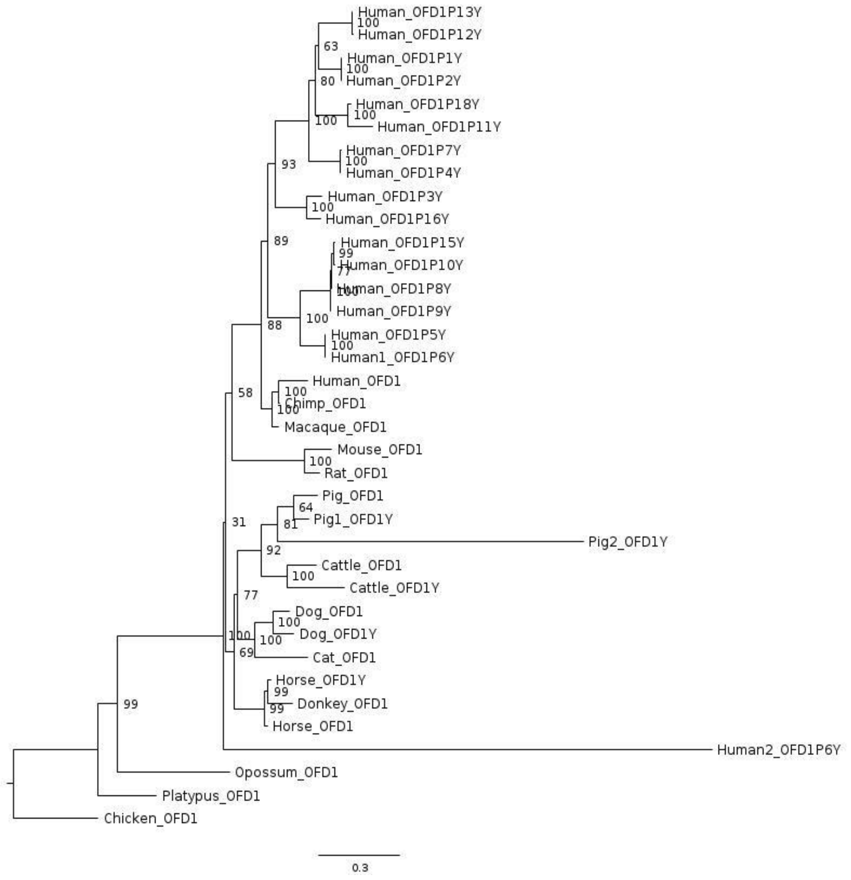

OR8JY

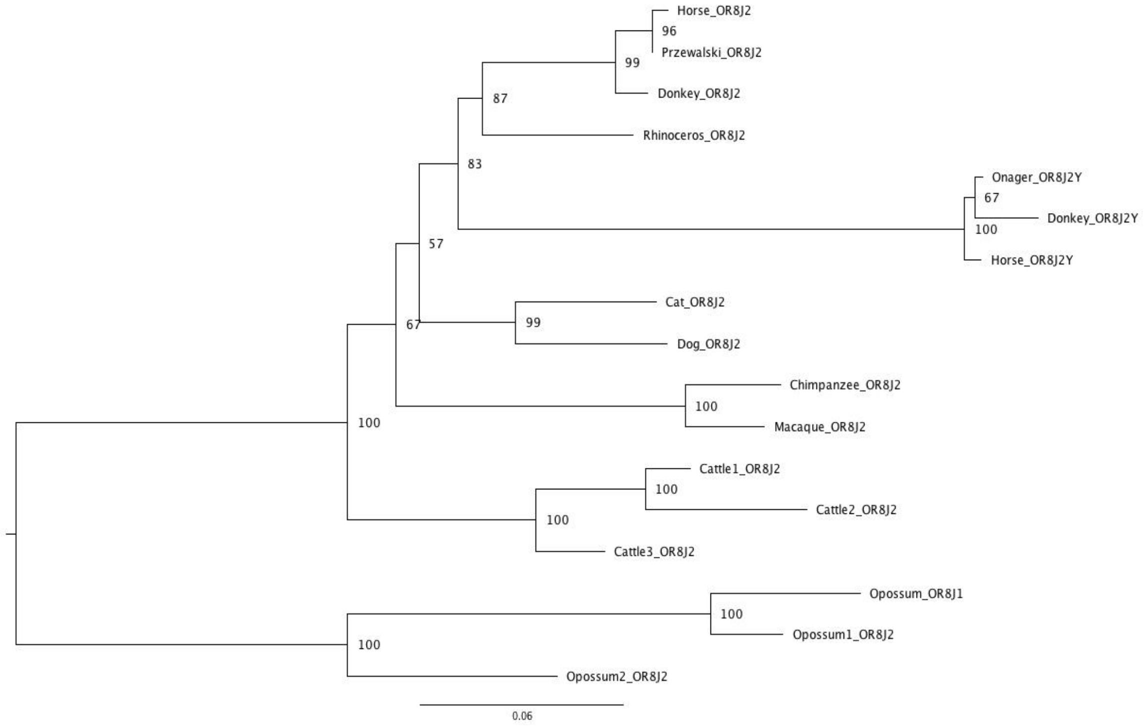

Supplementary Figure 10 continued
OR8K3Y

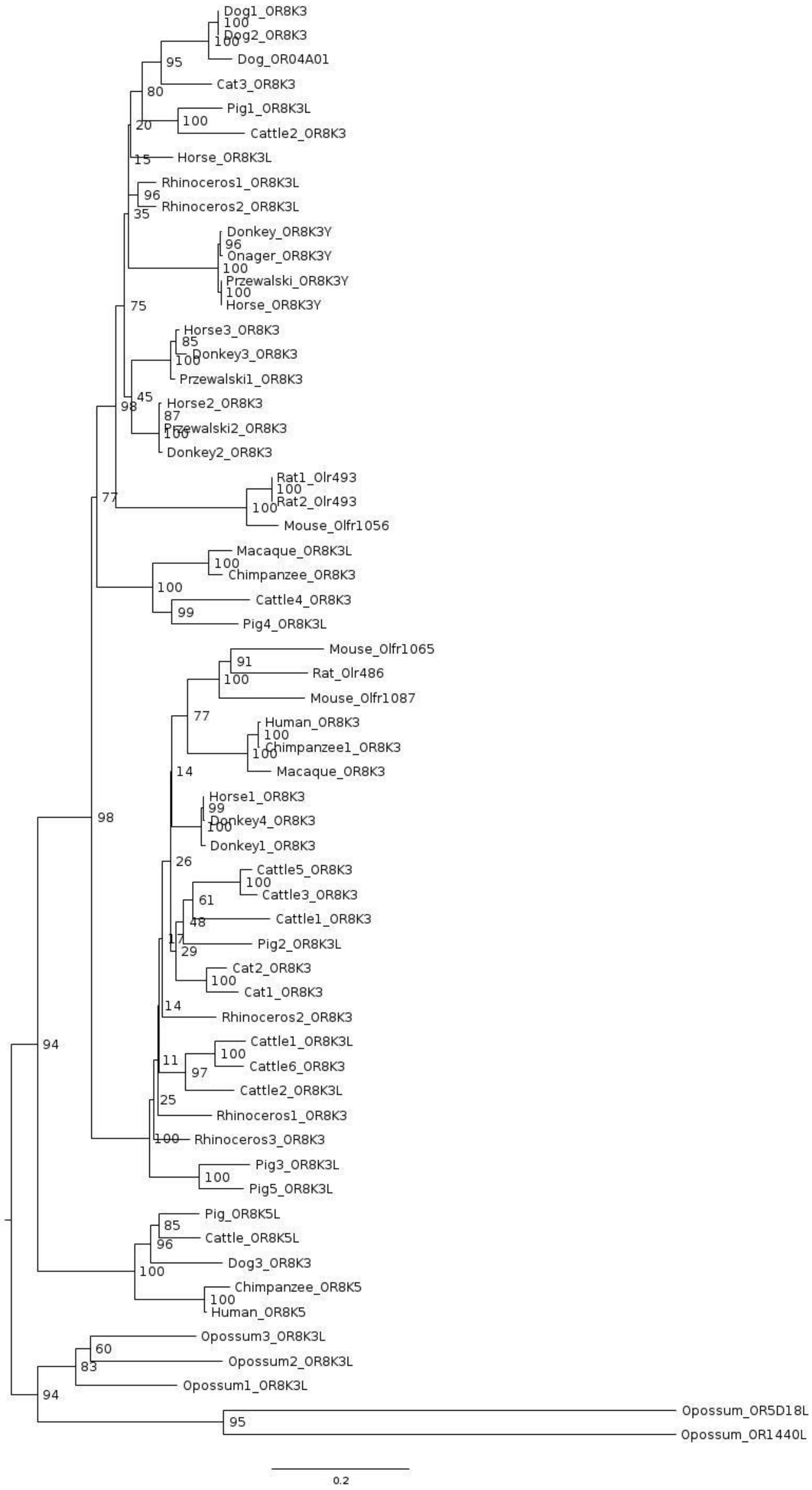

Supplementary Figure 10 continued
RBMY

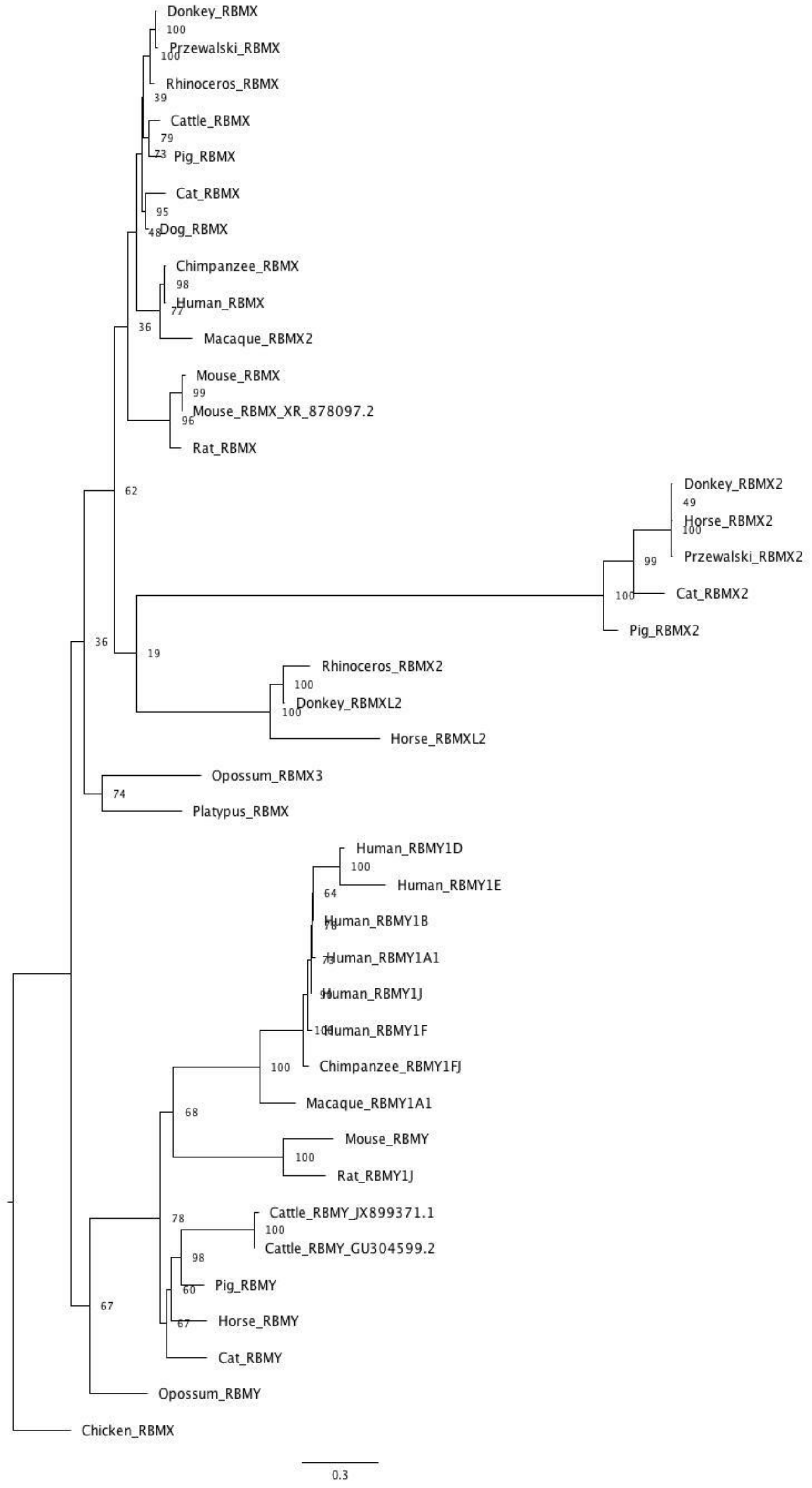

Supplementary Figure 10 continued
RPS3AY

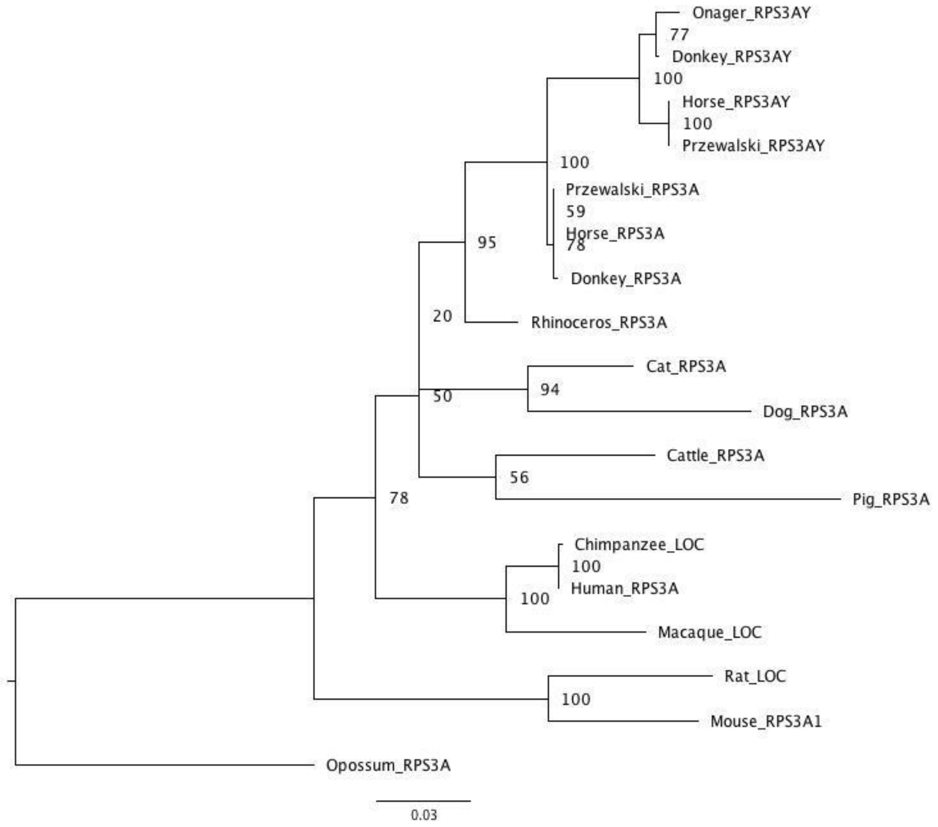

SH3TC1Y

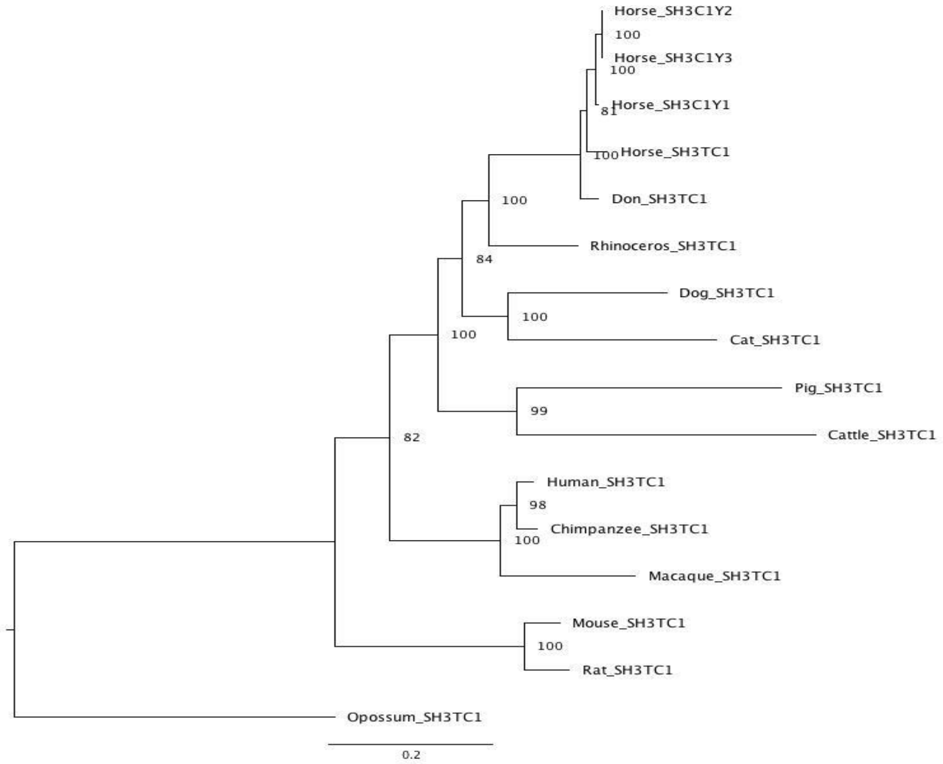

Supplementary Figure 10 continued
SHROOM2Y

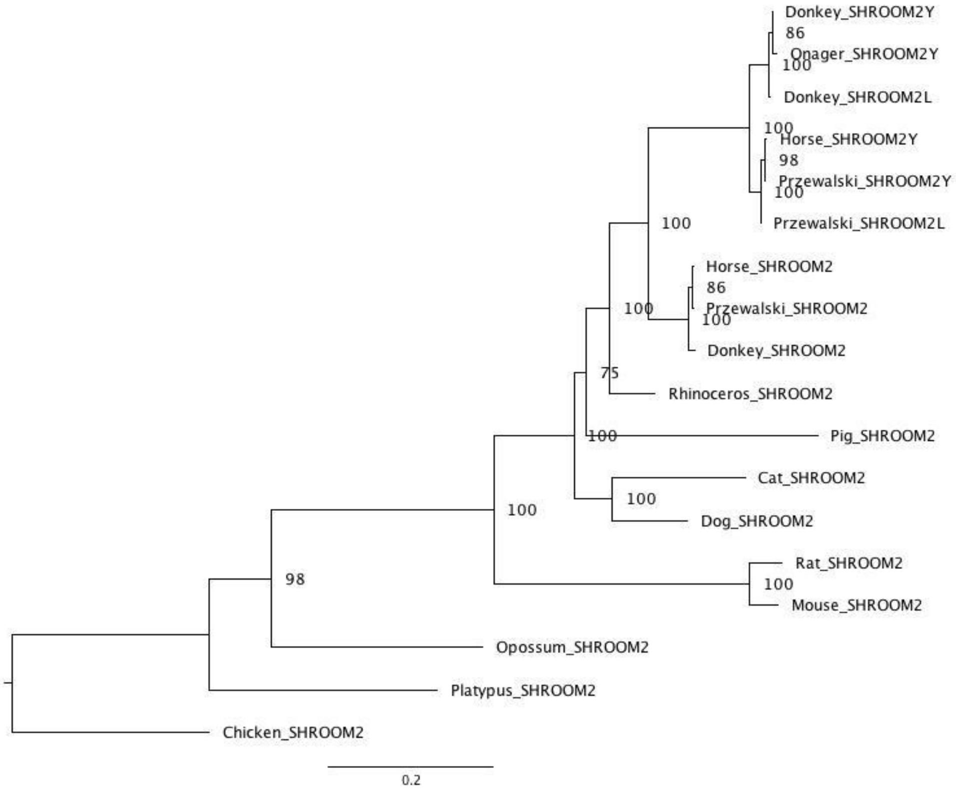

SRY

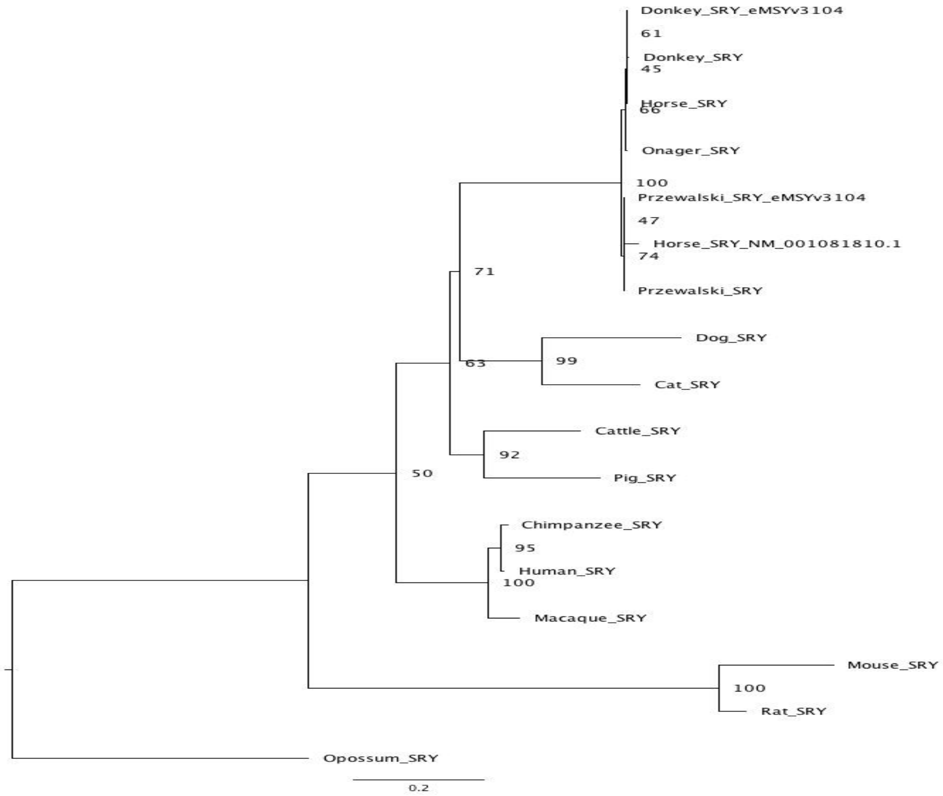

Supplementary Figure 10 continued

STSY

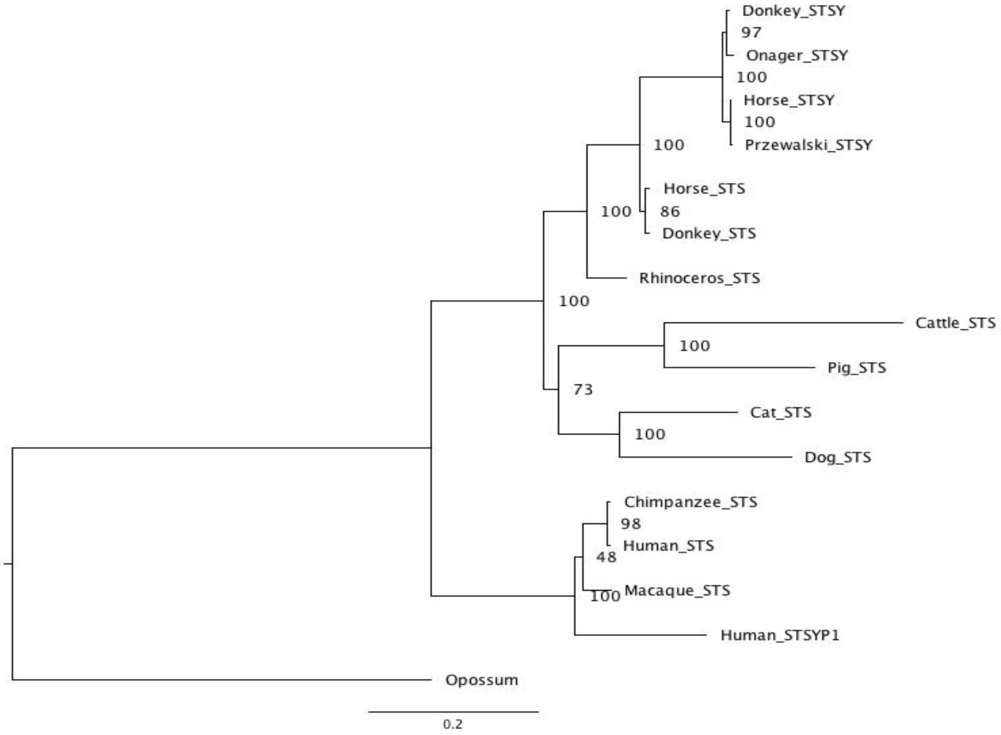

SYPY

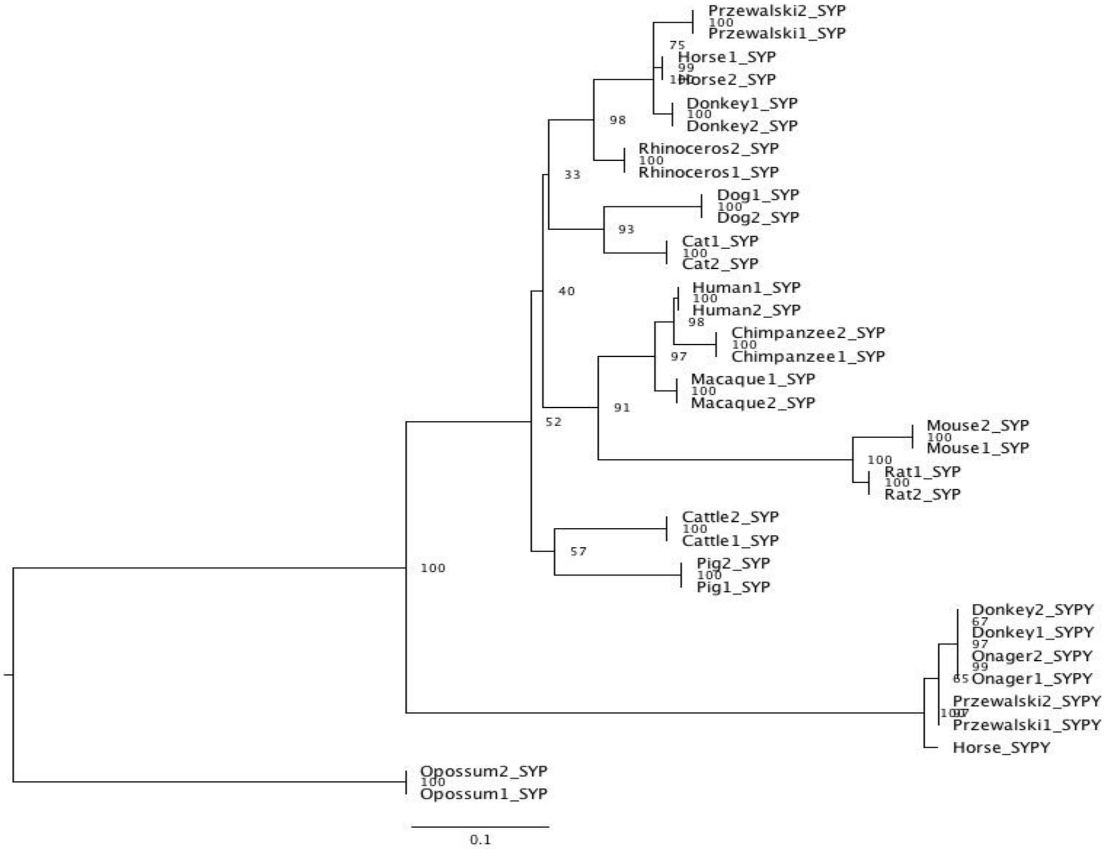

Supplementary Figure 10 continued

TAB3Y

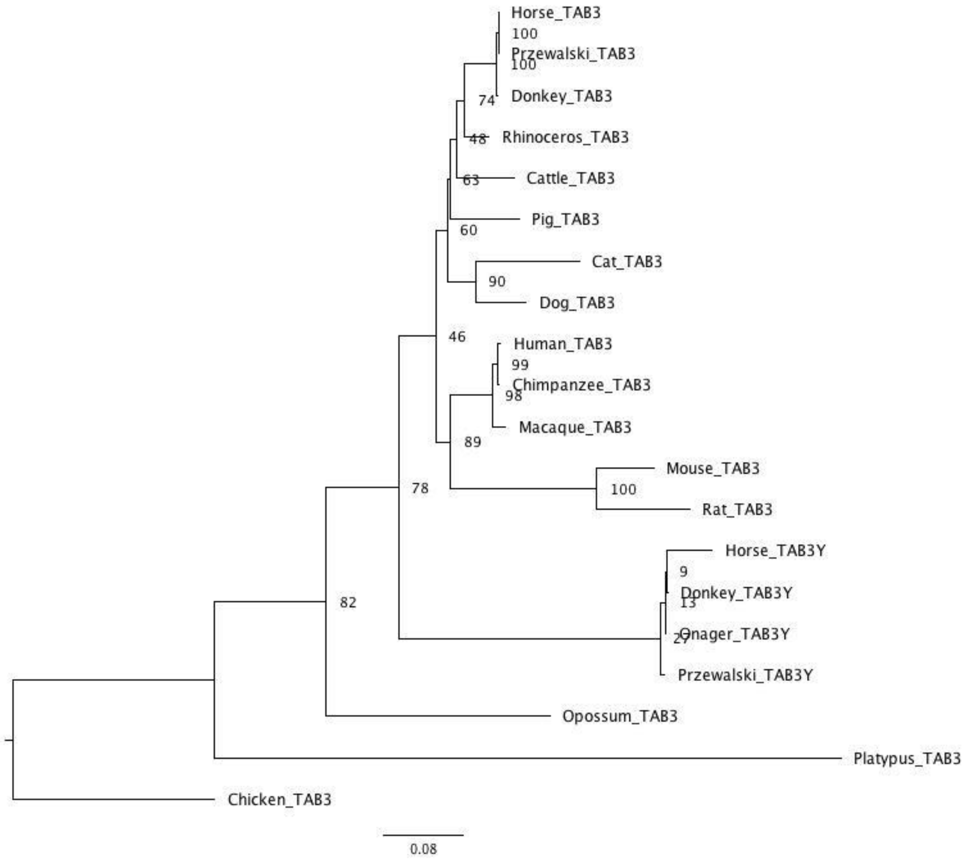

TBL1Y

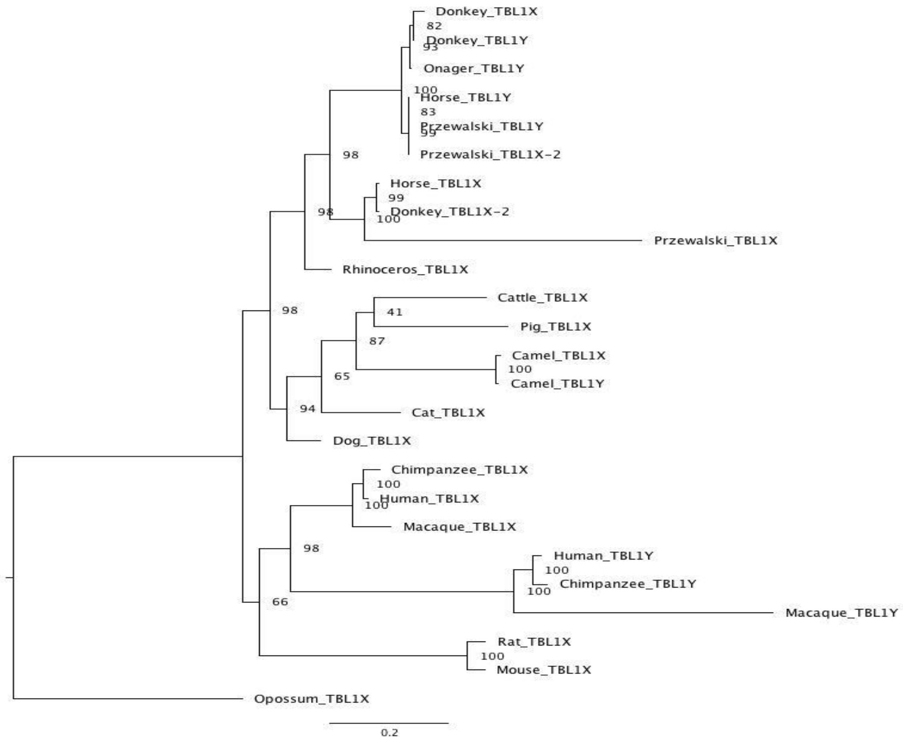

Supplementary Figure 10 continued
TIGD1Y

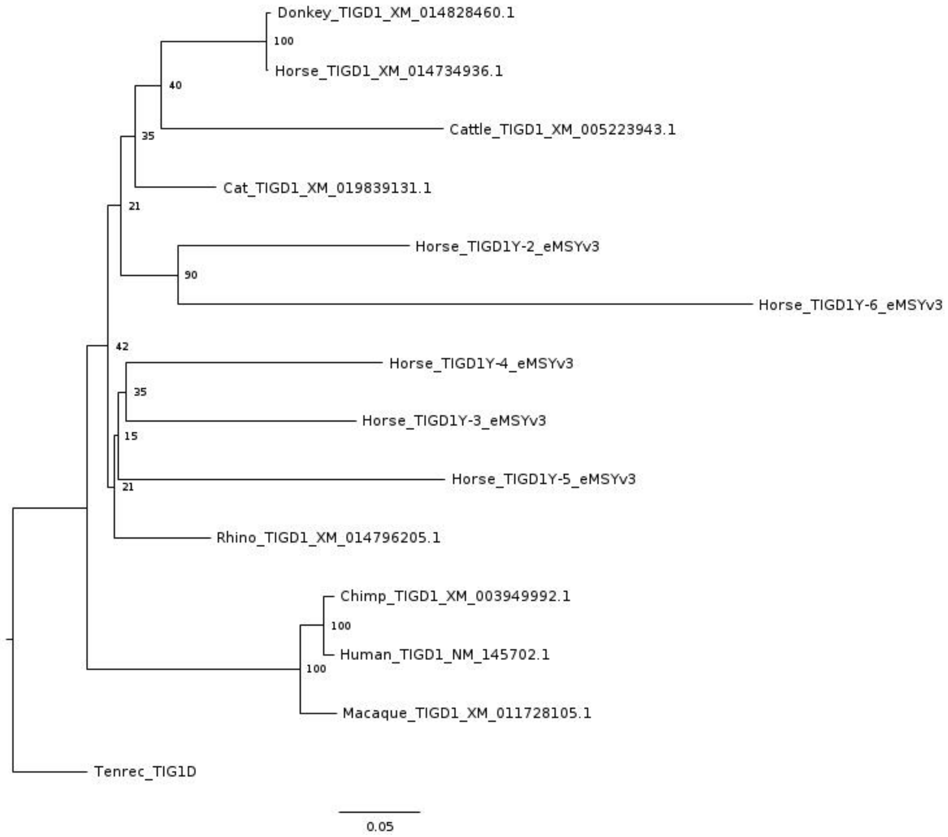

TMSB4Y

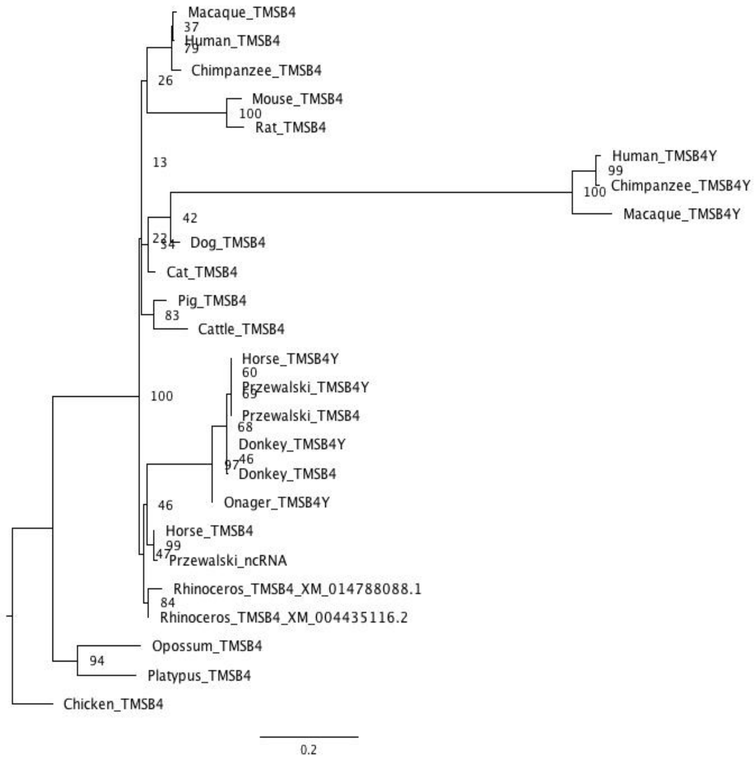

Supplementary Figure 10 continued
TSPY

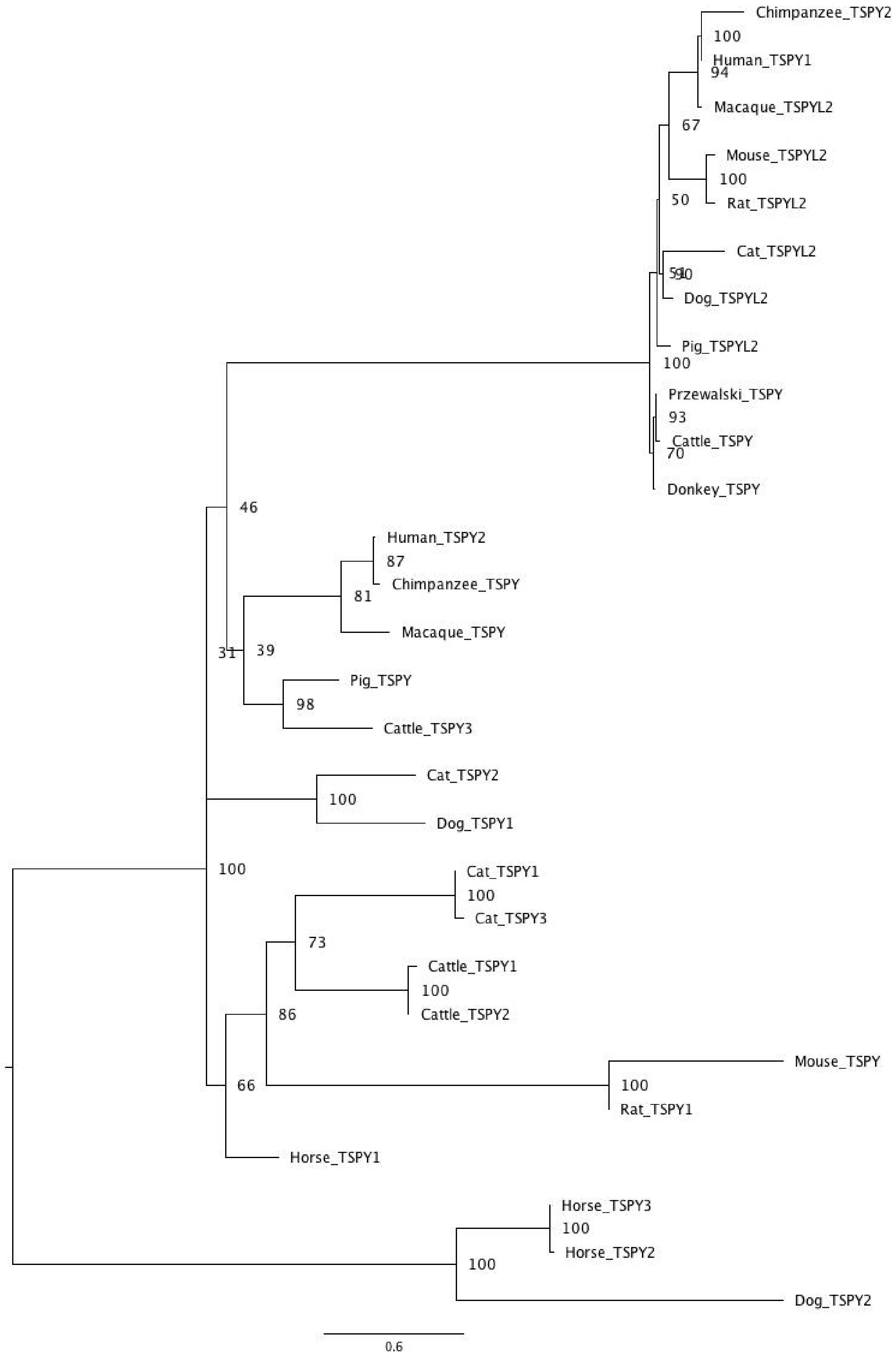

Supplementary Figure 10 continued

TXLNGY

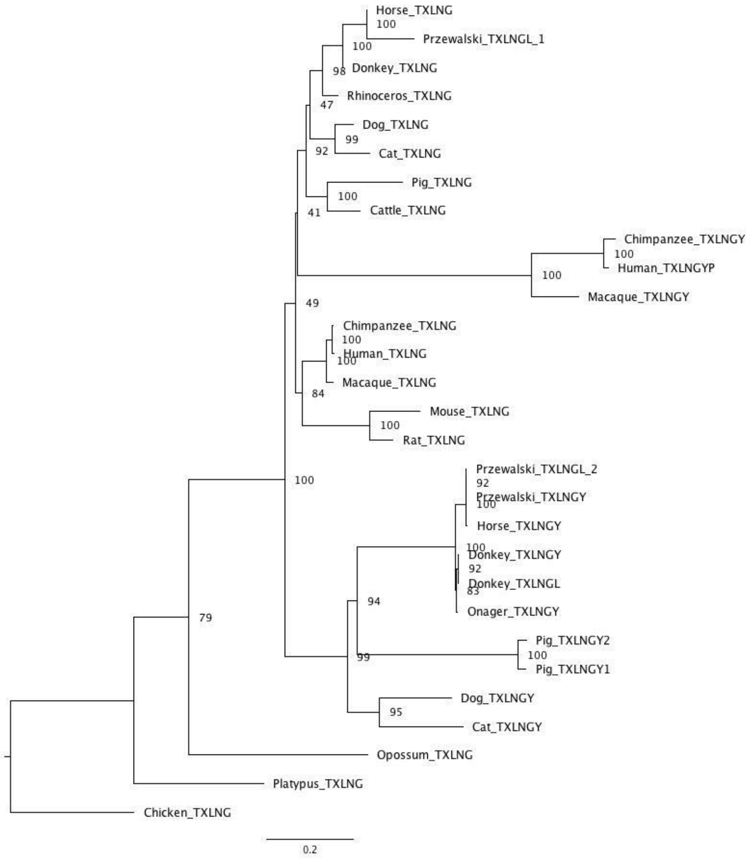

USP9Y

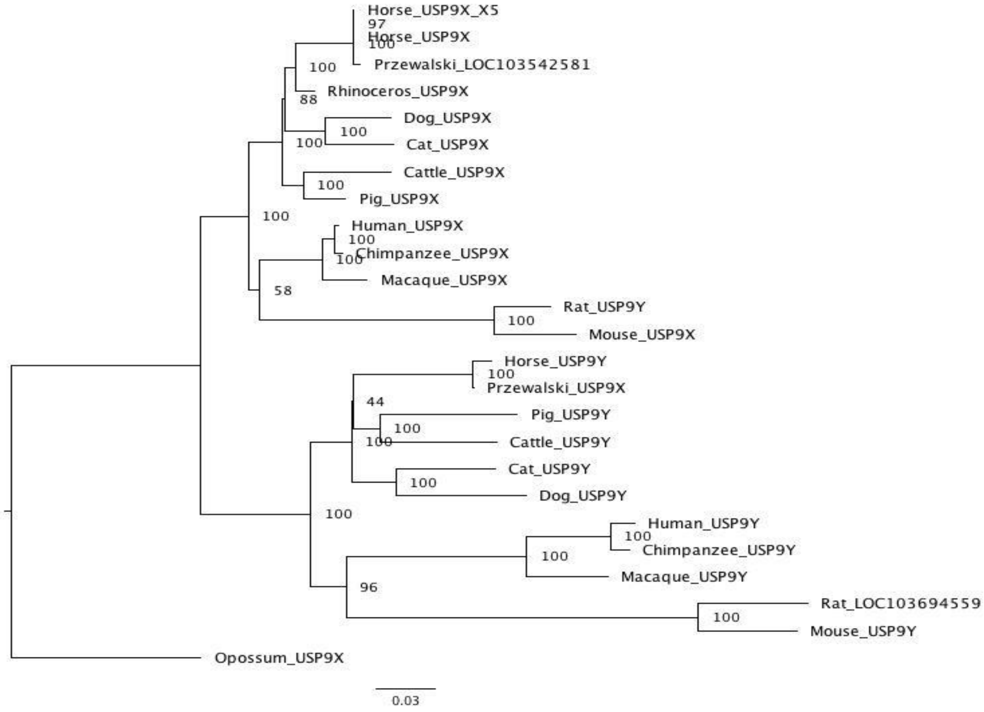

Supplementary Figure 10 continued
UBA1Y

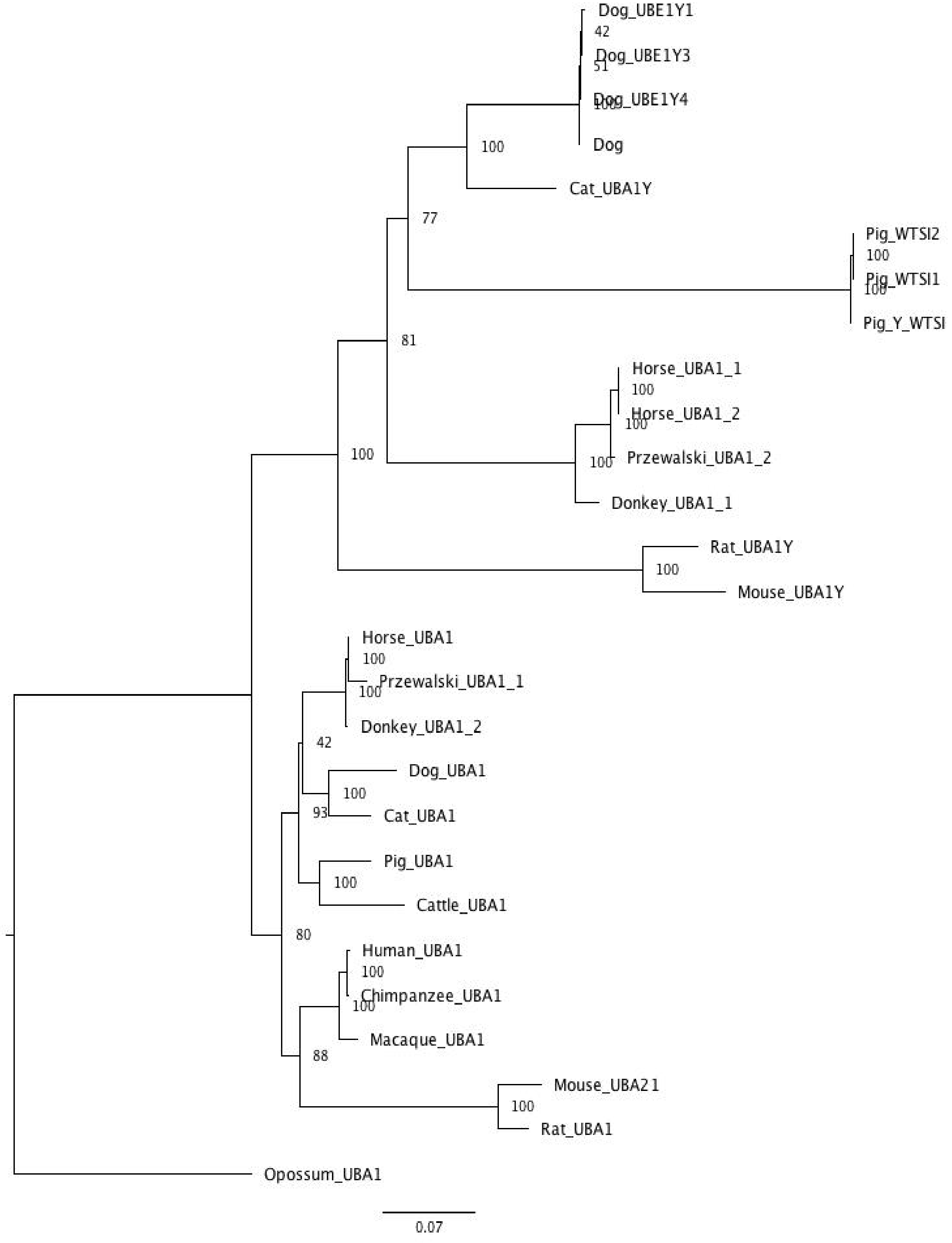

Supplementary Figure 10 continued
UTY

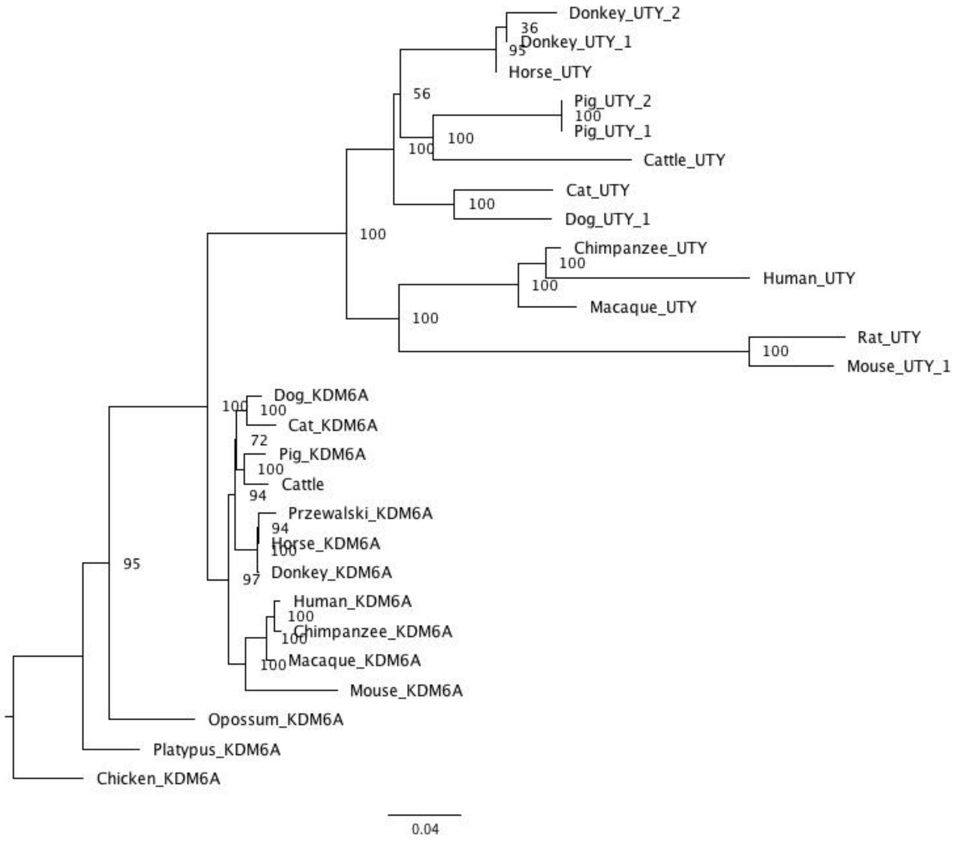

WWC3Y

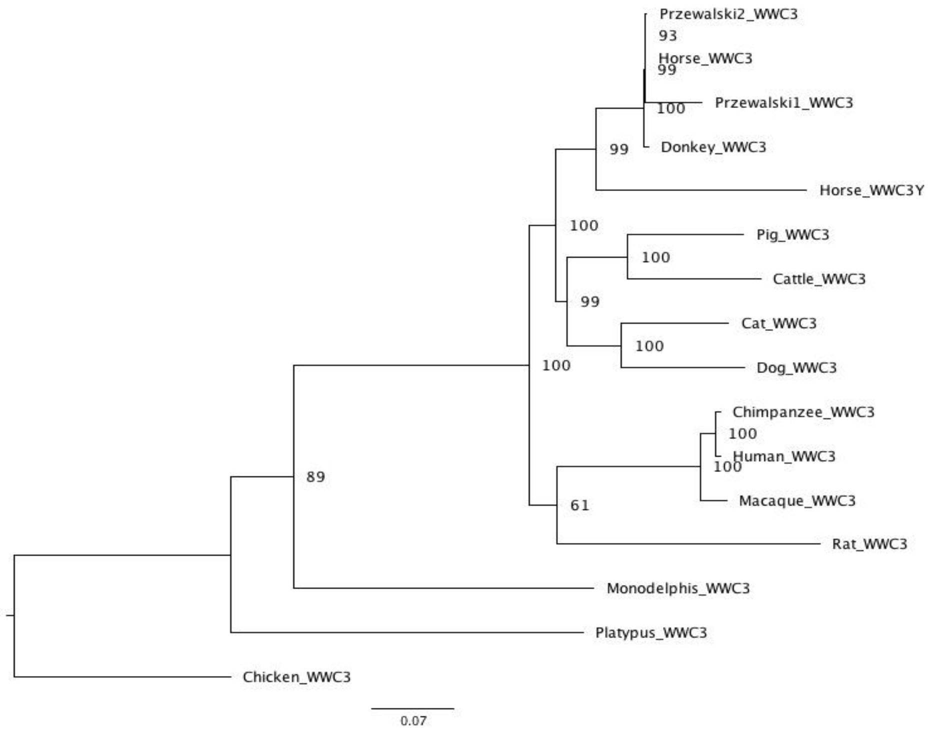

Supplementary Figure 10 continued
XKR3Y

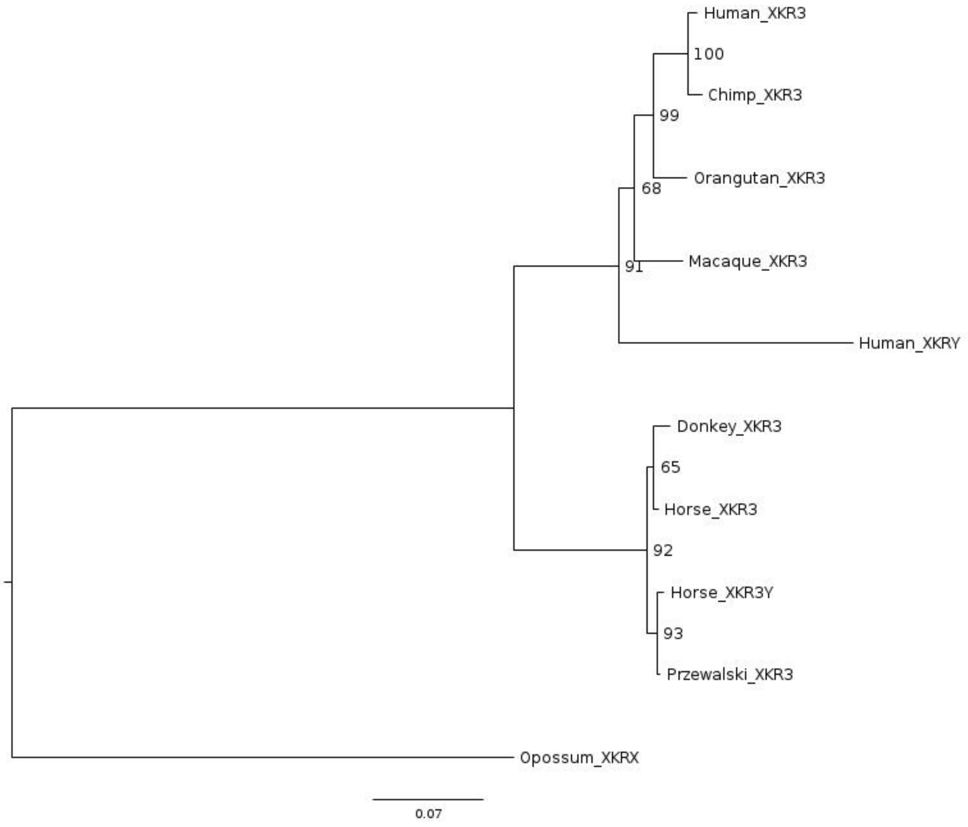

ZFY

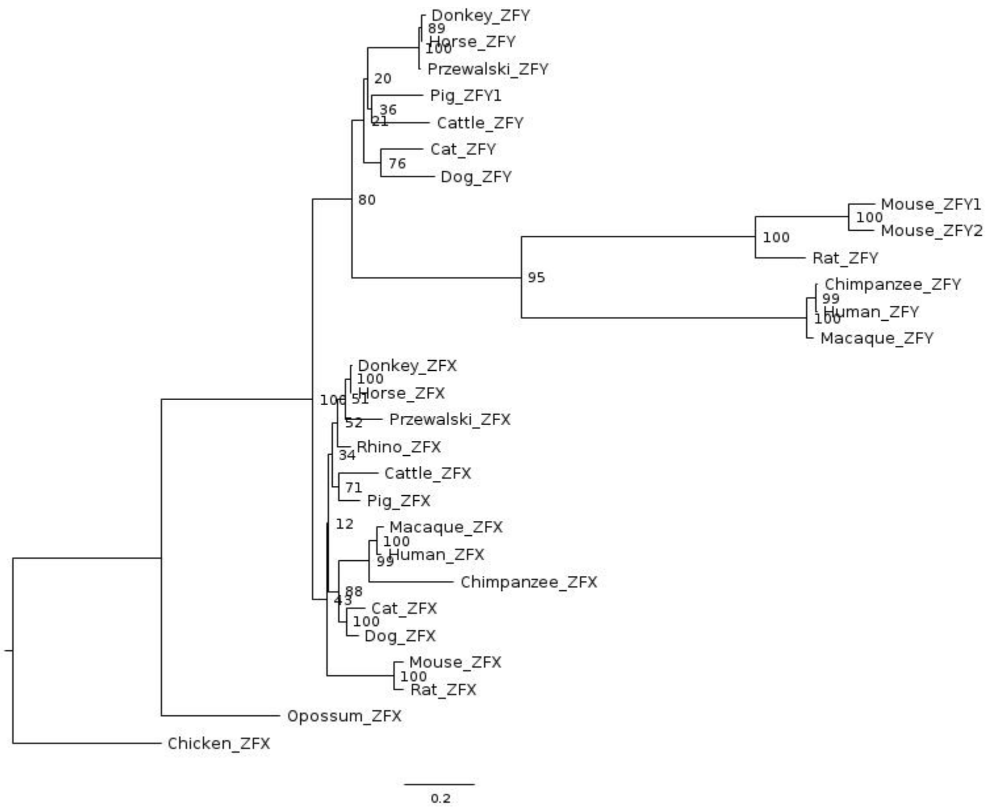

Supplementary Figure 10 continued
ZRSR2Y

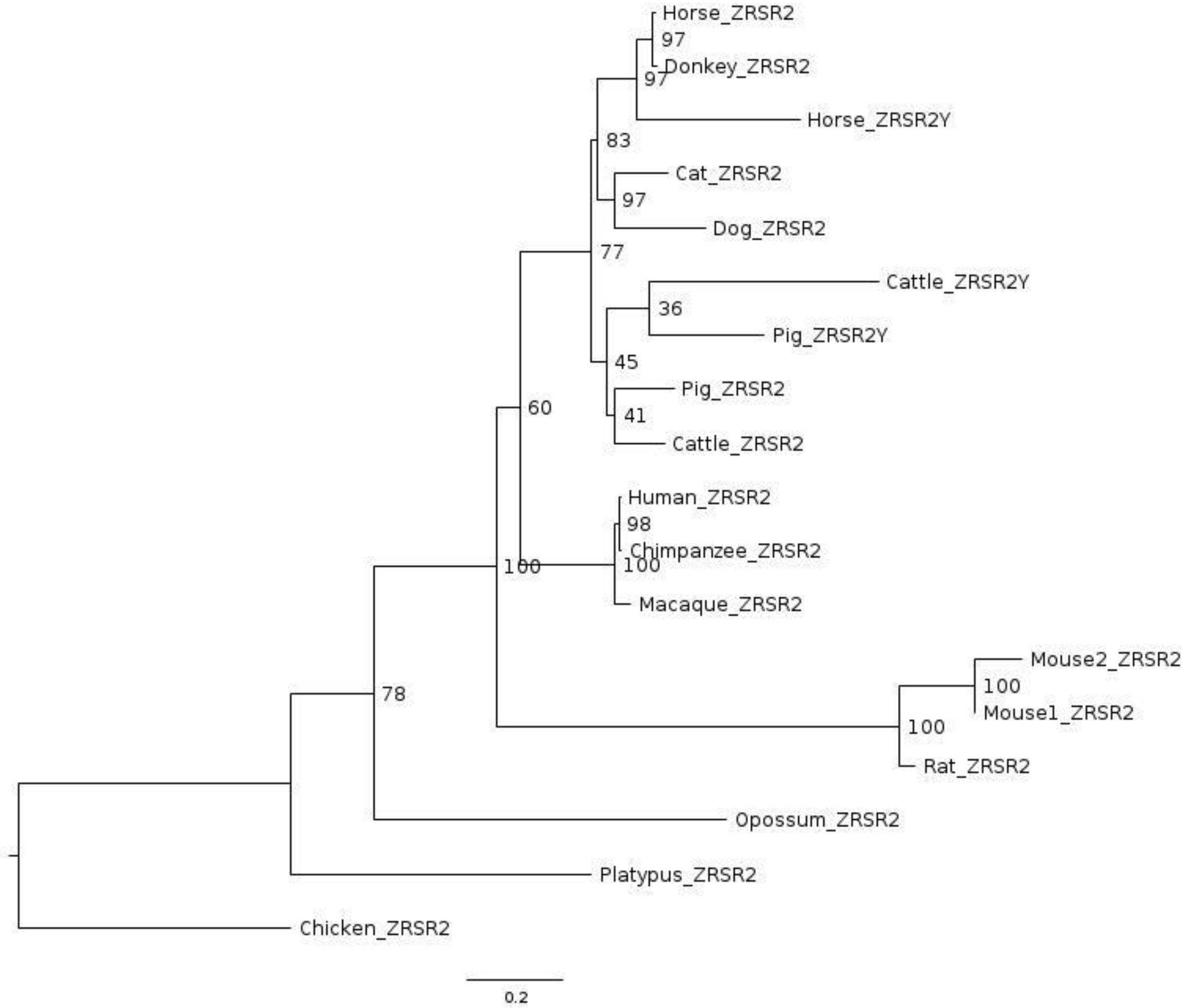

Supplementary Fig. 10. Maximum likelihood trees for 41 eMSY genes. Highest likelihood score reconstructed using RAxML version 8.2.4. Bootstrap value from 1000 replicates shown on nodes. Information for individual genes is in Supplementary Data 7.

Supplementary References

1. Raudsepp, T. et al. A detailed physical map of the horse Y chromosome. *Proc Natl Acad Sci U S A* **101**, 9321-9326 (2004).
2. Paria, N. et al. A gene catalogue of the euchromatic male-specific region of the horse Y chromosome: comparison with human and other mammals. *PLoS One* **6**, e21374 (2011).
3. Untergasser, A. et al. Primer3--new capabilities and interfaces. *Nucleic Acids Res* **40**, e115 (2012).
4. Milenkovic, D. et al. Cytogenetic localization of 136 genes in the horse: comparative mapping with the human genome. *Mamm Genome* **13**, 524-534 (2002).
5. Raudsepp, T. & Chowdhary, B.P. FISH for mapping single copy genes. *Methods Mol Biol* **422**, 31-49 (2008).
6. ISCNH International system for cytogenetic nomenclature of the domestic horse (Report of the Third International Committee for the Standardization of the domestic horse karyotype: Bowling, A. T., Breen, M., Chowdhary, B. P., Hirota, K., Lear, T., Millon, L. V., Ponce de Leon, F. A., Raudsepp, T., Stranzinger, G.) *Chromosome Res* **5**, 433-443 (1997).
7. Skaletsky, H. et al. The male-specific region of the human Y chromosome is a mosaic of discrete sequence classes. *Nature* **423**, 825-837 (2003).
8. Hughes, J.F. et al. Chimpanzee and human Y chromosomes are remarkably divergent in structure and gene content. *Nature* **463**, 536-539 (2010).
9. Soh, Y.Q. et al. Sequencing the mouse Y chromosome reveals convergent gene acquisition and amplification on both sex chromosomes. *Cell* **159**, 800-813 (2014).
10. Lange, J. et al. Isodicentric Y chromosomes and sex disorders as byproducts of homologous recombination that maintains palindromes. *Cell* **138**, 855-869 (2009).
11. Bellott, D.W. et al. Mammalian Y chromosomes retain widely expressed dosage-sensitive regulators. *Nature* **508**, 494-499 (2014).
12. Haas, B.J. et al. De novo transcript sequence reconstruction from RNA-seq using the Trinity platform for reference generation and analysis. *Nat Protoc* **8**, 1494-1512 (2013).
13. Hansen, K.D., Brenner, S.E. & Dudoit, S. Biases in Illumina transcriptome sequencing caused by random hexamer priming. *Nucleic Acids Res* **38**, e131 (2010).
14. Kozarewa, I. et al. Amplification-free Illumina sequencing-library preparation facilitates improved mapping and assembly of (G+C)-biased genomes. *Nat Methods* **6**, 291-295 (2009).
15. Li, B., Ruotti, V., Stewart, R.M., Thomson, J.A. & Dewey, C.N. RNA-Seq gene expression estimation with read mapping uncertainty. *Bioinformatics* **26**, 493-500 (2010).
16. Trapnell, C. et al. Transcript assembly and quantification by RNA-Seq reveals unannotated transcripts and isoform switching during cell differentiation. *Nat Biotechnol* **28**, 511-515 (2010).
17. Shcherbik, N., Wang, M., Lapik, Y.R., Srivastava, L. & Pestov, D.G. Polyadenylation and degradation of incomplete RNA polymerase I transcripts in mammalian cells. *EMBO Rep* **11**, 106-111 (2010).
18. Nielsen, M.K. et al. Parascaris univalens--a victim of large-scale misidentification? *Parasitol Res* **113**, 4485-4490 (2014).
19. Ghosh, S. et al. Copy number variation in the horse genome. *PLoS Genet* **10**, e1004712 (2014).

20. Jónsson, H. et al. Speciation with gene flow in equids despite extensive chromosomal plasticity. *Proc Natl Acad Sci U S A* **111**, 18655-18660 (2014).
