# Supplementary material for: Horse Y chromosome assembly displays unique evolutionary features and putative stallion fertility genes
Source: Nat Commun. 2018 Jul 27;9:2945. doi: 10.1038/s41467-018-05290-6 (PMC6063916; doi:10.1038/s41467-018-05290-6)
Supplement: Supplementary file 3 — Description of Additional Supplementary Files [file 41467_2018_5290_MOESM3_ESM.pdf]

Description of Additional Supplementary Files

File Name: Supplementary Data 1
Description: **a** Genome-wide testis RNAseq read coverage per gene per individual; **b** Genes significantly down-regulated in mules compared to both horse and donkey, and c GO enrichment for genes with 30X greater read depth in horse and donkey compared to mule.

File Name: Supplementary Data 2
Description: The eMSYv3.1 draft that is deposited in GenBank has two locations where two vector sequences were removed relative to eMSYv3.0 used for analysis in this manuscript. Therefore, in order for the coordinates in the GTF file to correspond to the correct positions, you must insert a 1,396-bp gap sequence (Ns) after position 8,048,430 and another 18,381-bp gap sequence (Ns) after position 2,506,950 into the eMSYv3.1 sequence. The total sequence length after making these two insertions should be 9,497,449 bp.

File Name: Supplementary Data 3
Description: SM file of all GENECONV results.

File Name: Supplementary Data 4
Description: Details for Sequence Tagged Sites (STSs) in eMSY tiling path.

File Name: Supplementary Data 5
Description: Details for BACs in eMSY tiling path and sequence assembly.

File Name: Supplementary Data 6
Description: Composite information on horse MSY genes and transcripts.

File Name: Supplementary Data 7
Description: Information on MSY gene paralogs and orthologs for time trees.

File Name: Supplementary Data 8
Description: Horse, donkey and mule Testis RNAseq read coverage per individual eMSY genes/transcripts.

File Name: Supplementary Data 9
Description: Samples for MSY comparison in equids and ancient and modern horses.

File Name: Supplementary Data 10
Description: Comparative data on MSY gene content in eutherian mammals (for Figure 8).

File Name: Supplementary Data 11
Description: Primers for RT-PCR and to validate eMSY assembly and HT.

File Name: Supplementary Data 12
Description: RNAseq GFF.

File Name: Supplementary Data 13
Description: Time tree constraints. Node constraints used to estimate divergence time for Y homologs are shown for each constrained node in the format used by the mcmctree program. For each gene, the best scoring maximum likelihood tree was obtained using RAxML. The time estimates are the confidence interval from TimeTree (www.timetree.org).

File Name: Supplementary Data 14
Description: MSY sequence coverage in equids and ancient and modern horses.
